# Clinical predictors for efficacy of erenumab for migraine: a Registry for Migraine (REFORM) study

**DOI:** 10.1093/braincomms/fcaf147

**Published:** 2025-04-15

**Authors:** William Kristian Karlsson, Messoud Ashina, Rune Häckert Christensen, Haidar Muhsen Al-Khazali, Håkan Ashina

**Affiliations:** Department of Neurology, Danish Headache Center, Copenhagen University Hospital—Rigshospitalet, Copenhagen 2600, Denmark; Department of Clinical Medicine, Faculty of Health and Medical Sciences, University of Copenhagen, Copenhagen 2100, Denmark; Department of Neurology, Danish Headache Center, Copenhagen University Hospital—Rigshospitalet, Copenhagen 2600, Denmark; Department of Clinical Medicine, Faculty of Health and Medical Sciences, University of Copenhagen, Copenhagen 2100, Denmark; Department of Neurology, Danish Headache Center, Copenhagen University Hospital—Rigshospitalet, Copenhagen 2600, Denmark; Department of Clinical Medicine, Faculty of Health and Medical Sciences, University of Copenhagen, Copenhagen 2100, Denmark; Translational Research Center, Copenhagen University Hospital—Rigshospitalet, Copenhagen 2600, Denmark; Department of Neurology, Danish Headache Center, Copenhagen University Hospital—Rigshospitalet, Copenhagen 2600, Denmark; Department of Clinical Medicine, Faculty of Health and Medical Sciences, University of Copenhagen, Copenhagen 2100, Denmark; Translational Research Center, Copenhagen University Hospital—Rigshospitalet, Copenhagen 2600, Denmark; Department of Neurology, Danish Headache Center, Copenhagen University Hospital—Rigshospitalet, Copenhagen 2600, Denmark; Translational Research Center, Copenhagen University Hospital—Rigshospitalet, Copenhagen 2600, Denmark

**Keywords:** headache, CGRP, prediction, preventive treatment, response

## Abstract

Erenumab has proven effective for migraine prevention; however, a substantial proportion of people with migraine do not benefit from treatment, and among those who do, there is considerable variability in response. This study aimed to identify clinical predictors of therapeutic response to erenumab and evaluate their predictive value in a large cohort of people with migraine. We conducted a single-centre, prospective, longitudinal cohort study of adults with migraine, experiencing ≥4 monthly migraine days. All participants received erenumab 140 mg monthly for 24 weeks and recorded their response in a headache diary with daily entries. A semi-structured interview was conducted at enrolment, and patient-reported outcome measures were collected before and after treatment. Treatment responders were classified as participants achieving a reduction from baseline of ≥50% in average monthly migraine days across weeks 13 through 24. Clinical predictors were analysed using logistic regression analysis. In total, 570 participants with migraine provided data eligible for analysis. Of these, 298 (52.3%) participants were classified as treatment responders, and the remaining 272 (47.7%) were non-responders. Independent predictors associated with a lower likelihood of response to erenumab were chronic migraine (odds ratio 0.63, 95% confidence interval 0.43–0.91; *P* = 0.030), daily headache (odds ratio 0.41, 95% confidence interval 0.24–0.67; *P* = 0.003) and previous failure of ≥3 preventive migraine medications (odds ratio 0.54, 95% confidence interval 0.37–0.77; *P* = 0.005). Conversely, better outcomes were observed with higher age (10-year increase: odds ratio 1.22, 95% confidence interval 1.06–1.41; *P* = 0.017). Multivariate model area under curve was 64.6% (60.0–69.2%). Participants with an early response to erenumab (≥50% reduction within weeks 1–12) were less likely than late responders to have chronic migraine [119/217 (57.1%) versus 61/79 (77.2%); *P* < 0.001], had lower Migraine Disability Assessment Scores [median (IQR): 52 (30–85) versus 65 (35–120); *P* = 0.029], more often had unilateral headache [193 (88.9%) versus 63/79 (79.7%); *P* = 0.041], and experienced less ictal allodynia measured by Allodynia Symptom Checklist-12 scores [median (IQR): 4 (0–8) versus 6 (2–8) versus; *P* = 0.024]. In conclusion, chronic migraine, experiencing daily headache, and having ≥3 preventive medication failures were independently associated with a lower likelihood of response to erenumab. Moreover, patients with a more severe clinical phenotype were more likely to respond later. Prediction of treatment responses might be improved by incorporating machine learning models and multimodal biomarkers, facilitating a shift towards personalized medicine.

## Teaser

Karlsson *et al.* report that in 570 patients with migraine from a tertiary headache centre, chronic migraine, daily headache and multiple prior preventive drug failures were associated with reduced efficacy of erenumab—a calcitonin-gene related peptide-antagonist—while older age predicted better outcomes. Severe phenotypes were linked to delayed responses.

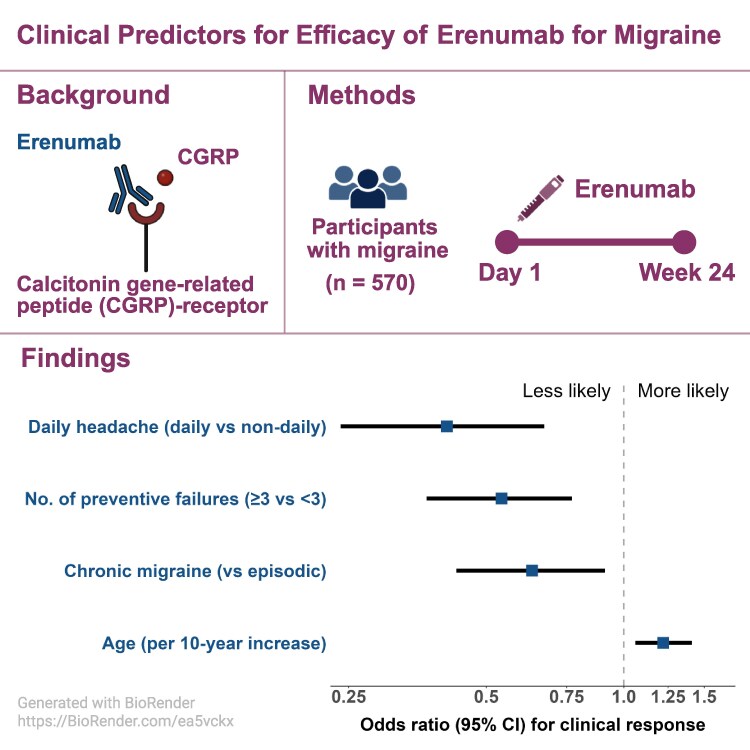

## Introduction

Migraine is a prevalent, disabling neurologic disorder, characterized by recurrent headache attacks and accompanying photophobia, phonophobia, nausea and vomiting.^1^ Its multifaceted pathogenesis provides basis for heterogeneous manifestations such as aura, cranial autonomic features and cutaneous allodynia.^2^ Since 2018, several medications targeting calcitonin gene-related peptide (CGRP) signaling have become available for migraine prevention.^3^ Erenumab, a monoclonal antibody targeting the CGRP-receptor, has been proven effective in clinical trials and real-world studies,^4^ with about half of the patients experiencing a ≥ 50% reduction in monthly migraine days (MMDs).^4,5^ However, the reasons for non-response and variable effectiveness remain unclear,^6^ leaving clinicians reliant on trial-and-error.

Real-world data suggest that response to CGRP-targeted mAbs, such as erenumab, might be influenced by certain clinical characteristics,^7-14^ including unilateral cranial autonomic features,^8,15^ responsiveness to triptans^9,10,12,16^ and specific headache characteristics.^8-10,17^ However, attempts to replicate these findings have provided inconsistent results,^18^ likely due to small sample sizes and differences in study design. Moreover, many potential clinical predictors remain unexplored. The high costs and restricted accessibility of CGRP-targeted mAbs further emphasize the need for reliable clinical predictors of treatment response.^19^ Identifying such predictors could enhance clinical decision-making and provide insights into the neurobiologic underpinnings of migraine.

In this study, we collected comprehensive clinical data and patient-reported outcome measures (PROMs) to investigate predictors for the effectiveness of erenumab in a large cohort of people with migraine.

## Materials and methods

### Study oversight

The study was approved by the relevant Health Research Ethics Committee and conducted in accordance with the Declaration of Helsinki, with written informed consent from all participants. The study was registered with ClinicalTrials.gov (NCT04603976) and reported following STROBE guidelines.^20^

### Study design and participants

Data presented herein is from the parental Registry for  Migraine (REFORM) study, a longitudinal, observational study conducted at a single tertiary headache centre. Details of the study design and eligibility criteria has been published elsewhere.^21^ In brief, eligible participants were adults (≥18 years) with a ≥1-year diagnosis of migraine per the International Classification of Headache Disorders, 3rd edition (ICHD-3).^22^ Participants had to report ≥4 MMDs at screening and during the 4-week baseline before initiating erenumab. Key exclusion criteria were any personal history of a secondary headache disorder (except for medication-overuse headache) and use of a CGRP-targeted monoclonal antibody in the 3 months preceding enrolment. Participants were permitted to use concomitant preventive headache medication(s), if administered at stable dose for ≥2 months before enrolment.

### Clinical data

Study data were collected from enrollment through completion of the 24-week erenumab treatment period.^21^ This included a 2-week screening period (Week −6 to −5), 4-week baseline period (Week −4 to Day 1) and 24-week treatment period (Day 1 to Week 24), with 140-mg erenumab injected subcutaneously every 4 weeks as part of a separate investigation (NCT04265755).

At the screening visit, a semi-structured interview was conducted to collect sociodemographic and clinical data, medical and medication history. A panel of PROMs was collected at Day 1 (i.e. first dosing of erenumab) and Week 24. These PROMs included the Allodynia Symptom Checklist-12 (ASC-12),^23^ Headache Impact Test-6 (HIT-6),^24^ Hospital Anxiety and Depression Scale,^25^ Migraine Disability Assessment (MIDAS)^26^ and World Health Organization Disability Assessment Schedule 2.0 (WHODAS 2.0).^27^ Participants completed a paper headache diary with daily entries from the start of the 4-week baseline period to the end of the 24-week treatment period (Supplementary Material 1).

### Outcomes

Efficacy of erenumab was assessed using headache diaries, comparing the baseline period to weeks 13–24. We assessed reductions in average MMDs—defined as the average number of days per month with a headache fulfilling migraine criteria—along with monthly headache days (MHDs) of any pain intensity and moderate-to-severe MHDs.^28^ PROMs were assessed by comparing MIDAS and HIT-6 scores at baseline and Week 24.

For each outcome, response was defined as either treatment response (favourable) or non-response (unfavourable). For the primary outcome, treatment responders were participants achieving a ≥ 50% reduction in average MMDs. Secondary outcomes were the following: (i) ≥ 50% reduction in average MHDs and (ii) ≥50% reduction in average MMDs or moderate-to-severe MHDs. Exploratory outcomes included the following: (i) ≥ 50% reduction in moderate-to-severe MHDs, (ii) ≥ 5-point reduction in MIDAS scores for participants with baseline scores between 11 and 20 points or a ≥ 30% reduction for those with baseline scores ≥21 and (iii) ≥ 5-point reduction in HIT-6. Cut-off values for MIDAS and HIT-6 were selected based on previous reports, suggesting that these reflect a clinical meaningful improvement.^29,30^

### Statistical analysis

The sample size was based on enrolment in the REFORM study.^21^ We excluded from the analyses participants who (i) did not receive all six erenumab injections or (ii) had poor compliance with the headache diary (<21 days completed per month during baseline or weeks 13–24). To address the impact of discontinuations due to adverse events, sensitivity analyses were conducted in which we included these participants as non-responders, reflecting treatment failure with erenumab.

Continuous data are reported as means ± standard deviations or medians with interquartile ranges. Data distribution was assessed with histograms and quartile-to-quartile plots. Baseline demographic and clinical data were compared between responders and non-responders using *t* tests or Mann–Whitney U-tests, while categorical data were analysed with Fisher’s exact or Chi-square tests.

Logistic regression was used to assess clinical predictors of therapeutic response. Details on classification of variables are available in the Supplementary Material 1. Covariates that yielded a *P*-value <0.1 in univariate analysis were included in a multivariable logistic regression model. Odds ratios (ORs) with 95% confidence intervals (CIs) were presented. We used chronic migraine for our main model and, due to multicollinearity, ran separate models in which we replaced chronic migraine with (i) daily headache and (ii) MHD and MMD (Supplementary Material 1).

Discriminative ability was measured using receiver operating characteristic curves to compute the area under the curve, with optimal thresholds determined by Youden's *J* statistic. Key diagnostic measures, including accuracy, sensitivity and specificity, were derived with 95% CIs using bootstrapping (2000 resamples). Calibration was assessed via calibration plots and the Hosmer–Lemeshow test (*P* > 0.05 indicated adequate calibration). Internal validation was performed using bootstrap resampling (2000 resamples), adjusting area under the curve for optimism (Supplementary Material 1).

*Post hoc* analyses explored partial responders (30–49% reduction) and compared early (≥50% MMD reduction across weeks 1 through 24) versus late responders (≥50% MMD reduction only in weeks 13–24). A mixed model for repeated measures was used to compare the change in mean MMDs between early and late responders. Further, McNemar's test was used to compare the classification of treatment responses at different timepoints (Supplementary Material 1).

Missing data from covariates in participants eligible for analysis was <5. Complete case analysis was used instead of multiple imputation, as we considered missing outcome data not missing at random.^31^ Statistical significance was set at *P* < 0.05, using the Benjamini–Hochberg procedure to control for multiple testing.^32^ All statistical analyses were conducted in R (version 4.4.1).^33^

## Results

**Figure 1:**
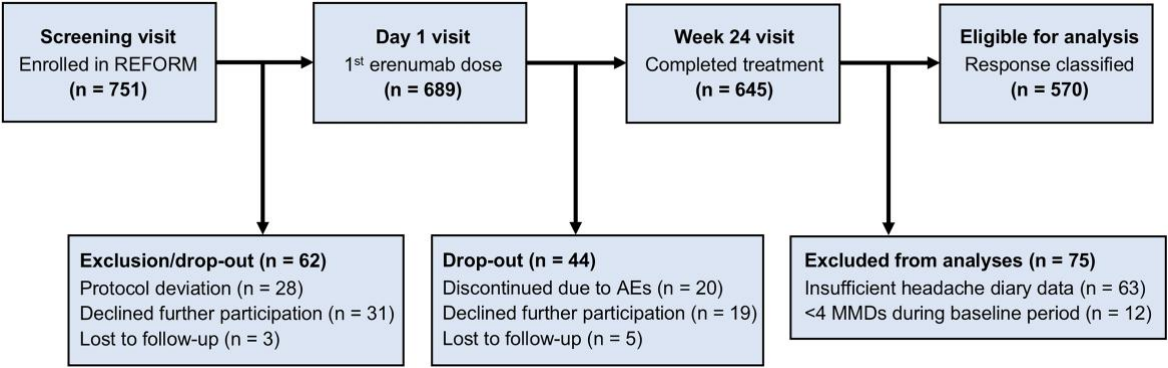
**Study flow.** Flow chart showing number of participants who were enroled in Registry for Migraine (REFORM) study, received first injection at Day 1, completed treatment with erenumab (Week 24) and provided sufficient data for classification of treatment response. Erenumab (140 mg subcutaneous injection every 28th day) was administered as part of a separate, open-label, single-arm study. AEs, adverse events; REFORM, Registry for Migraine.

Of 751 participants enroled from September 2020 to June 2022, 689 completed the baseline period and received their first injection of 140-mg erenumab. Among these, 570 (82.7%) completed the 24-week treatment and provided efficacy data eligible for analysis. Figure 1 details study flow and reasons for exclusion or dropout.

From baseline to weeks 13–24, 298 participants (52.3%) achieved a ≥ 50% reduction in average MMDs (responders), while 272 (47.7%) had <50% reduction (non-responders). The mean change in MMDs (comparing baseline with weeks 13–24) was −9.4 ± 4.1 MMDs in responders and −2.0 ± 3.9 MMDs in non-responders *(P* < 0.001; Mann–Whitney U-test).

### Baseline demographics and clinical characteristics

Table 1 summarizes the baseline demographic and clinical characteristics of the study population, comparing treatment responders and non-responders. The mean age was 44.7 ± 12.2 years, with 90.4% (*n* = 515) being female. Chronic migraine was reported by 65.4% (*n* = 373), daily headache by 15.8% (*n* = 90) and 29.5% (*n* = 168) had a history of migraine with aura. Low-frequency episodic migraine (≤9 MHDs) was reported by 48 (8.4%) participants (clinical characteristics available in Supplementary Tables 1 and 2). Preventive migraine medication was used by 48.9% (*n* = 279), and 33.2% (*n* = 189) had ≥3 preventive medication failures.

**Table 1:** Baseline demographics and characteristics of the study population

	Total (*n* = 570)	Responders^a^ (*n* = 298)	Non-responders^a^ (*n* = 272)	*P* ^b^
**Demographic characteristics**				
Age, mean ± SD, years	44.7 ± 12.2	46.1 ± 12.1	43.2 ± 12.2	0.005^c^
Female sex, *n* (%)	515 (90.4%)	265 (88.9%)	250 (91.9%)	0.23^d^
Racial identity, White, *n* (%)	564 (98.9%)	295 (99.0%)	269 (98.9%)	>0.99^e^
Overweight (BMI ≥ 25 kg/m^2^), *n* (%)	246 (43.2%)	120 (40.3%)	126 (46.3%)	0.15^d^
Obesity (BMI ≥ 30 kg/m^2^), *n* (%)	93 (16.3%)	40 (13.4%)	53 (19.5%)	0.050^d^
**Migraine history**				
Age at onset, median (IQR), years	18 (12–25)	17 (12–25)	18 (13–26)	0.25^f^
Migraine disease duration, median (IQR), years	23 (15–33)	25 (17–35)	21 (13–29)	<0.001^f^
First degree relative with migraine, *n* (%)	399 (70.0%)	206 (69.1%)	193 (71.0%)	0.63^d^
Migraine with aura, *n* (%)	168 (29.5%)	87 (29.2%)	81 (29.8%)	0.88^d^
Chronic migraine, *n* (%)	373 (65.4%)	181 (60.7%)	192 (70.6%)	0.014^d^
Daily headache (28-day baseline), *n* (%)	90 (15.8%)	30 (10.1%)	60 (22.1%)	<0.001^d^
Medication-overuse, *n* (%)	324 (56.8%)	173 (58.1%)	151 (55.5%)	0.54^d^
Headache frequency (28-day baseline), median (IQR)				
MHDs	17 (13–25)	16 (12–23)	18 (13–26)	0.006^f^
MMDs	12 (9–16)	12 (9–16)	12 (8–16)	0.31^f^
Monthly acute medication days	10 (8–15)	9 (7–12)	9 (7–13)	0.67^f^
Use of preventive migraine medication, *n* (%)	279 (48.9%)	151 (50.7%)	128 (47.1%)	0.39^d^
Preventive medications failures (lack of efficacy), *n* (%)				<0.001^d^
<3	381 (66.8%)	219 (73.5%)	162 (59.6%)	
≥3	189 (33.2%)	79 (26.5%)	110 (40.4%)	
Triptan resistance (lack of efficacy), *n* (%)	118 (21.7%)	54 (19.1%)	64 (24.5%)	0.13^d^
**Disability**				
MIDAS score, median (IQR)	54 (30–96)	55 (31–95)	51 (29–100)	0.98^f^
HIT-6 score, median (IQR)	63 (61–66)	63 (61–66)	63 (60–65)	0.077^f^
WHODAS 2.0, median (IQR)	21 (17–28)	21 (17–28)	22 (17–27)	0.61^f^
**Comorbidities**				
Somatic comorbidities, *n* (%)				
Autoimmune disorders	72 (12.6%)	42 (14.1%)	30 (11.0%)	0.27^d^
Asthma	58 (10.2%)	33 (11.1%)	25 (9.2%)	0.46^d^
Constipation	101 (17.7%)	57 (19.1%)	44 (16.2%)	0.36^d^
Daily low back pain	53 (9.3%)	31 (10.4%)	22 (8.1%)	0.34^d^
Daily neck pain	83 (14.6%)	50 (16.8%)	33 (12.1%)	0.12^d^
Hypertension	61 (10.7%)	27 (9.1%)	34 (12.5%)	0.18^d^
Psychiatric comorbidities, *n* (%)				
HADS anxiety score ≥8	157 (28.4%)	83 (28.9%)	74 (27.9%)	0.80^d^
HADS depression score ≥8	131 (23.7%)	63 (22.0%)	68 (25.7%)	0.31^d^

^a^Participants were classified according to their reduction in MMDs as responders (≥50%) or non-responders (<50%).

^b^ *P*-values reported for the comparison between responders and non-responders.

^c^Unpaired t test.

^d^Pearson Chi-squared test.

^e^Fisher’s exact test.

^f^Mann–Whitney U-test.

BMI, body mass index; HADS, Hospital Anxiety and Depression Scale; HIT-6, Headache Impact Test-6; IQR, interquartile range; MIDAS, Migraine Disability Assessment Test; MHDs, monthly headache days; MMDs, monthly migraine days; WHODAS, World Health Organization Disability Assessment Schedule.

Responders were older than non-responders (mean ± SD: 46.1 ± 12.1 versus 43.2 ± 12.2; *P* = 0.005; unpaired *t* test), had longer disease duration [median (IQR): 25 (17–35) versus 21 (13–29); *P* < 0.001; Mann–Whitey U-test], fewer MHDs [median (IQR): 16 (12–23) versus 18 (13–26); *P* = 0.006; Mann–Whitey U-test], and were less likely to have chronic migraine [181/298 (60.7%) versus 192/272 (70.6%); *P* = 0.014; Pearson’s Chi-squared] and daily headache [30/298 (10.1%) versus 60/272 (22.1%); *P* < 0.001; Pearson’s Chi-squared]. Failure of ≥3 preventive medications due to lack of efficacy was also less frequent in responders compared with non-responders [79/272 (26.5%) versus 110/298 (40.4%); *P* < 0.001; Pearson’s Chi-squared]. No differences were identified the groups in terms of failures to particular preventive drug classes (Supplementary Table 3). Other demographic and clinical characteristics did not differ significantly between responders and non-responders (Table 1).

Table 2 summarizes migraine attack features for the total study population and for responders and non-responders. There were no differences between groups in terms of headache location, side-locked headache, pulsating pain, pain intensity, physical activity aggravation, associated symptoms, unilateral cranial autonomic features or baseline allodynia (ASC-12) scores.

**Table 2:** Migraine attack features of the study population

	Total (*n* = 570)	Responders^a^ (*n* = 298)	Non-responders^a^ (*n* = 272)	*P* ^b^
**Migraine attack features**				
Unilateral headache, *n* (%)	487 (85.4%)	258 (86.6%)	229 (84.2%)	0.42^c^
Side-locked	83 (14.6%)	42 (14.1%)	41 (15.1%)	0.74^c^
Pulsating pain quality, *n* (%)	418 (73.3%)	219 (73.5%)	199 (73.2%)	0.93^c^
Pain intensity (4-point scale), *n* (%)				0.62^d^
Mild	3 (0.5%)	1 (0.3%)	2 (0.7%)	
Moderate	181 (31.1%)	91 (30.5%)	89 (32.7%)	
Severe	398 (68.4%)	206 (69.1%)	181 (66.5%)	
Headache aggravated by physical activity, *n* (%)	530 (91.1%)	277 (93.0%)	243 (89.3%)	0.13^c^
Associated symptoms, *n* (%)				
Photophobia	551 (95.5%)	283 (95.9%)	257 (95.2%)	0.81^c^
Phonophobia	525 (91.0%)	270 (91.5%)	246 (91.1%)	0.97^c^
Nausea	528 (91.5%)	275 (93.2%)	243 (90.0%)	0.12^c^
Vomiting	333 (57.7%)	176 (59.7%)	149 (55.2%)	0.27^c^
Unilateral cranial autonomic symptoms, *n* (%)	123 (21.2%)	60 (20.4%)	60 (22.1%)	0.63^c^
Ictal allodynia; ASC-12, median (IQR)	3 (0–7)	4 (0–8)	3 (0–6)	0.079^e^

^a^Participants were classified according to their reduction in MMDs as responders (≥50%) or non-responders (<50%).

^b^ *P*-values reported for the comparison between responders and non-responders.

^c^Pearson Chi-squared test.

^d^Fisher’s exact test.

^e^Mann–Whitney U-test.

ASC-12, Allodynia Symptom Checklist-12; IQR, interquartile range.

### Predictors of clinical response to erenumab

In the following, we report the results of the multivariable logistic regression analyses. The Supplementary material contains the full results of the univariate and multivariable logistic regression analyses (Supplementary Materials 2 and 3), diagnostic metrics (Supplementary Table 4), receiver operating characteristic (Supplementary Figs. 1–6) and calibration curves (Supplementary Figs. 7–12). The number of participants who were classified as treatment responders had outcome data and were analysed in each multivariate model are presented in Supplementary Table 5. Information on missing covariate data is available in Supplementary Tables 6 and 7.

#### Primary outcomes

Table 3 presents the findings from the multivariable logistic regression analysis, including data from 549 participants with complete data. For achieving a ≥50% reduction in MMDs from baseline to weeks 13–24, independent predictors for a lower likelihood of responding to erenumab were chronic migraine (OR 0.63; 95% CI 0.43–0.91; *P* = 0.030), daily headache (OR 0.41; 95% CI 0.24–0.67; *P* = 0.003) and ≥3 failed preventive migraine (versus <3 failed: OR 0.54; 95% CI 0.37–0.77; *P* = 0.005). Conversely, increasing age was associated with greater likelihood of response to erenumab (10-year increase: OR 1.22; 95% CI 1.06–1.41; *P* = 0.017). There was no association between ≥50% reduction in MMDs with baseline MHDs (1-day increase: 0.97; 95% CI 0.95–1.00; *P* = 0.067) or MMDs (univariate analysis; 1-day increase: OR 1.00; 95% CI 0.97–1.03; *P* = 0.96).

**Table 3:** Multivariable logistic regression

Outcome: ≥ 50% reduction in MMDs (*n* = 549)			
Covariates	OR (95% CI)	*P*	*P_adj_* ^b^
Age (10-year increase)	1.22 (1.06–1.41)	0.006	0.017
Obesity (BMI ≥30 kg/m^2^)	0.63 (0.39–1.02)	0.060	0.085
No. of preventive medication failures (≥3 versus <3)	0.54 (0.37–0.77)	<0.001	0.005
HIT-6 score (1-point increase)	1.04 (1.00–1.09)	0.071	0.085
Ictal allodynia; ASC-12 (1-point increase)	1.03 (0.99–1.08)	0.125	0.125
Chronic migraine (CM versus EM)	0.63 (0.43–0.91)	0.015	0.030
Daily headache (28-day baseline)^a^	0.41 (0.24–0.67)	<0.001	0.003
Monthly headache days (1-day increase)^a^	0.97 (0.95–1.00)	0.034	0.067

^a^Estimates and 95% CIs derived from refitting model, with ‘Chronic migraine’ replaced by either ‘Daily headache’ or ‘Monthly headache days’. Monthly migraine days were not significantly associated with the outcome in univariate analysis (*P* = 0.96).

^b^ *P*-value adjusted for multiple testing.

ASC-12, Allodynia Symptom Checklist-12; BMI, body mass index; CI, confidence interval; CM, chronic migraine; EM, episodic migraine; HIT-6, Headache Impact Test-6; MMDs, monthly migraine days; OR, odds ratio.

Replacing age with migraine disease duration yielded comparable results. Similarly, using overweight instead of obesity, and number of failed drug classes instead of the total number of failed preventive medications, produced comparable outcomes. No association was found between the failure of particular drug classes and erenumab response. The inclusion of participants who discontinued erenumab due to adverse events had no material effect on the results (Supplementary Material 4).

#### Secondary outcomes

For a ≥ 50% reduction in MHDs, chronic migraine had a lower likelihood of therapeutic response (OR 0.51; 95% CI 0.35–0.75; *P* = 0.005), as did those with daily headache (OR 0.22; 95% CI, 0.11–0.42; *P* < 0.001), a higher number of MHDs (1-day increase: OR 0.93; 95% CI 0.91–0.96; *P* < 0.001), and ≥3 preventive medication failures (versus <3 failed: OR 0.64; 95% CI 0.44–0.94; *P* = 0.046), while, conversely, greater likelihood of response was found with increasing age (10-year increase: OR 1.20; 95% CI 1.04–1.40; *P* = 0.041) and presence of unilateral headache (versus bilateral: OR 2.31; 95% CI 1.34–4.09; *P* = 0.012).

For a combined outcome of a ≥ 50% reduction in MMDs or moderate-severe MHDs, higher age increased response likelihood (10-year age increase: OR 1.34; 95% CI 1.15–1.57; *P* < 0.001), whereas less likelihood of treatment response was found in those with chronic migraine (OR 0.58; 95% CI 0.39–0.85; *P* = 0.014), daily headache (OR 0.44; 95% CI 0.27–0.71; *P* = 0.002), hypertension (OR 0.52; 95% CI 0.29–0.93; *P* = 0.035) and ≥3 preventive medications failures (versus <3 failed: OR 0.62; 95% CI 0.43–0.90; *P* = 0.020).

#### Exploratory outcomes

Analyses for the exploratory outcomes (≥50% reduction in moderate-to-severe MHDs, MIDAS score reduction and HIT-6 score reduction) overall aligned with findings from primary and secondary outcomes (Supplementary Material 3).

### Performance of the multivariable models and internal validity

The multivariable model predicting a ≥ 50% reduction in MMDs showed an area under the curve of 64.6% (95% CI 60.0–69.2%), indicating possible helpful discrimination.^34^ Internal validity was adequate, with an optimism-corrected area under the curve of 62.6%. At the optimal classification threshold, the model had an accuracy of 62.1% (95% CI 56.8–65.2%), sensitivity of 62.1% (95% CI 56.8–67.7%) and specificity of 60.2% (95% CI 54.2–66.3%). The calibration plot and Hosmer–Lemeshow test (*P* = 0.071) indicated acceptable fit at all but the lowest probabilities (Supplementary Fig. 1). Secondary and exploratory models showed similar performance metrics, with poor fit for ≥50% reduction in MHDs (*P* = 0.004), while others were adequate (Supplementary Table 4).

### Timepoint for assessing response

Using data from different time periods, the accuracy of correctly predicting response status at weeks 13–24 improved from 79.7% (95% CI 76.1–82.9%) using data from weeks 1–12 to 88.0% (95% CI 85.0–90.6%) with week 5–16 data, and 90.5% (95% CI 87.7–92.8%) using week 9–20 data. A significant difference was found in classification between weeks 1–12 and 13–24 (*P* < 0.001; McNemar’s test), but none was found when comparing weeks 5–16 (*P* = 0.11; McNemar’s test) or weeks 9–20 (*P* = 0.34; McNemar’s test) with weeks 13–24.

### Partial responders for each main predictive clinical characteristic

**Figure 2:**
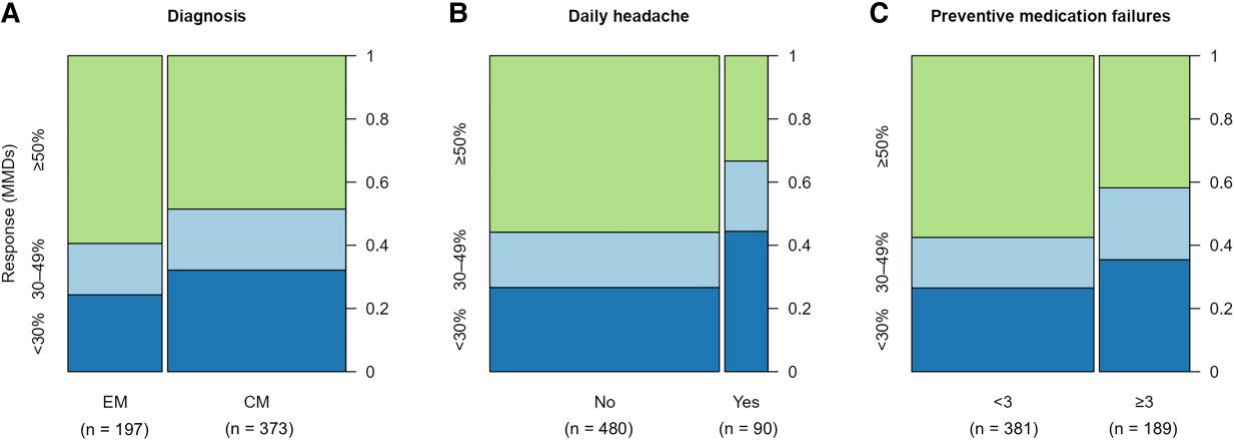
**Distribution of participants by treatment response and key predictors.** Spinograms for the following key binary predictive characteristics: (**A**) chronic migraine, (**B**) daily headache and (**C**) preventive medication failures. Each panel illustrates the distribution of participants across three categories of treatment response: ≥50% (green), 30–49% (light blue) and <30% (dark blue) reduction in monthly migraine days (MMDs). The size of each square corresponds to the proportion of participants in each response category. CM, chronic migraine; EM, episodic migraine; MMDs, monthly migraine days.

Partial responders to erenumab treatment were defined as participants achieving a 30–49% reduction in MMDs from baseline to weeks 13–24. Partial response (30–49% reduction in MMDs) was observed in 19.3% (*n* = 72) of participants with chronic migraine, 22.5% (*n* = 20) with daily headache and 22.8% (*n* = 43) with ≥3 preventive failures (Fig. 2). Across these groups, mean change in MMDs ranged from −5.8 to −6.8 days, while clinically meaningful improvement was observed in 52.6–68.6% measured by MIDAS, and 31.6–45.0% measured by HIT-6 (Supplementary Tables 8 and 9).

### Patterns of treatment response

A total of 557 (99.5%) participants had headache diary data for both the first 12 weeks (weeks 1–12) and the final 12 weeks (weeks 13–24) of erenumab treatment. Of these, 217 (39.0%) showed an early and sustained response, with a ≥ 50% reduction in average MMDs during both periods. In contrast, 79 (14.2%) had a late response only in the final 12 weeks. Additionally, 36 (6.5%) participants responded early but not late, while 234 (42.0%) were non-responders throughout both periods.

**Figure 3:**
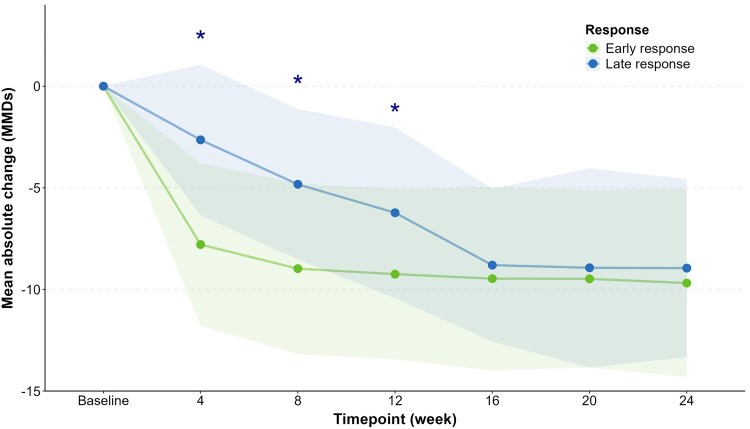
**Early versus late responders.** Plot depicting the mean absolute change in monthly migraine days (MMDs) over time. Data points represent the mean change, while shaded areas indicate the corresponding standard deviations (SDs). A negative value reflects a reduction in MMDs. Participants who achieved an early and sustained response to erenumab (defined as ≥50% reduction in MMDs from week 1 through week 24) are shown in green (*n* = 217), and those who achieved a late response (defined as ≥50% reduction in MMDs from week 13 through week 24) are shown in blue (*n* = 79). Asterisks (*) indicate significant differences (*P* < 0.05) between early and late responders at each time point, as determined by mixed-effects models.

Figure 3 presents the mean (SD) change from baseline in MMDs for each 4-week interval for early and late responders to erenumab. Based on a mixed model for repeated measures, early responders experienced a greater change in MMDs than late responders within weeks 1–4 (−2.6 ± 3.7 versus −7.8 ± 4.0; *P* < 0.001), weeks 5–8 (−4.8 ± 3.7 versus −9.0 ± 4.2; *P* < 0.001) and weeks 9–12 (−6.2 ± 4.2 versus −9.3 ± 4.2; *P* < 0.001). However, no significant differences in change in MMDs were observed within weeks 13–16 (−8.8 ± 3.8 versus −9.5 ± 4.5; *P* = 0.28), weeks 17–20 (−8.9 ± 4.9 versus −9.5 ± 4.4; *P* = 0.33) or weeks 21–24 (−9.0 ± 4.4 versus −9.7 ± 4.6; *P* = 0.19).

Late responders were more likely than early responders to have chronic migraine [61/79 (77.2%) versus 119/217 (57.1%); *P* < 0.001; Pearson’s Chi-squared], reporting more MHDs [median (IQR): 19 (14–26) versus 15 (12–21); *P* = 0.003; Mann–Whitney U-test] and higher MIDAS scores [median (IQR): 65 (35–120) versus 52 (30–85); *P* = 0.029; Mann–Whitney U-test; Supplementary Table 10]. In terms of attack features, late responders less often had unilateral headache [63/79 (79.7%) versus 193 (88.9%); *P* = 0.041; Pearson’s Chi-squared] and higher ASC-12 scores [median (IQR): 6 (2–8) versus 4 (0–8); *P* = 0.024; Mann–Whitney U-test; Supplementary Table 11).

## Discussion

In this prospective, longitudinal study of 570 adults treated with erenumab, daily headache consistent emerged as a negative predictor of response across all efficacy outcomes. Participants with chronic migraine, a higher number of MHDs and ≥3 preventive migraine medication failures were also less likely to respond to erenumab, as measured across several efficacy outcomes. In contrast, older age and the presence of unilateral headache increased the likelihood of response. Lastly, late responders more often had chronic migraine, less often unilateral headache, higher levels of migraine-related disability and higher scores of cutaneous allodynia.

### Predictive clinical characteristics

Our results align well with those from a recently published European multicentre study involving participants with migraine who were treated with various CGRP-targeted mAbs.^7^ Similar findings have also been reported in smaller studies,^8,13,14,17,35-37^ indicating that a higher headache frequency is associated with a worse likelihood of response to CGRP-targeted mAbs. Importantly, although daily headache was a strong negative predictor, one-third of participants with daily headache still responded to erenumab, indicating treatment should still be considered for this subgroup.

Another important aspect for patients with migraine being considered for treatment with CGRP mAbs pertains to previous preventive medication failures—a common criterion for obtaining reimbursement. Our findings align with previous reports, showing that a greater number of prior preventive medication failures reduces the likelihood of a favourable response to mAbs targeting CGRP signaling.^8,10,12,14,35,38^ This pattern of resistance could reflect the cumulative impact of delayed effective treatment as suggested by recent trial evidence.^39^ Delays in initiating appropriate therapy can arise from factors such as misdiagnosis, limited access to specialized care and prolonged reliance on suboptimal treatments. These factors may contribute to maladaptive changes in the trigeminovascular system or migraine-relevant cerebral networks, ultimately reducing treatment responsiveness.^40^ Additionally, disease progression may be influenced by comorbidities, lifestyle factors and disease-specific mechanisms, further complicating treatment outcomes.^40^ A recent genome-wide association study on migraine revealed several risk loci within genes relevant to the therapeutic targets of migraine-specific treatments, such as the CGRP molecule; however, specific genetic variations that directly impact the response to erenumab have yet to be identified.^41^ Notably, a recent study found that while switching among CGRP mAbs provides some benefit in patients who switched, overall response rates were lower compared to non-switchers, with outcomes deteriorating after multiple switches.^42^ Overall, these findings emphasize the link between multiple preventive failures and poorer outcomes, likely due to treatment delays and underlying disease mechanisms.

Previous studies have provided conflicting reports regarding the impact of age on the efficacy of mAbs against CGRP signaling.^7,8,12,43-45^ In our cohort, older age increased the likelihood of responding to erenumab across several efficacy outcomes. The mechanisms behind this association remain elusive; however, older age is generally accompanied by a decrease in migraine prevalence and intensity of migraine pain.^46,47^ While delay to effective treatment has been associated with worse outcomes, as discussed above, it is important to consider that the potential effects of older age may act independently of the influences of treatment delay. Age-related physiological changes, such as alterations in the trigeminovascular and hormonal systems,^46^ may underlie differences in treatment responses, leading to greater efficacy of erenumab in older individuals. Further studies in diverse populations are warranted to elucidate the complex interplay between age, treatment timing, and response to erenumab. Of interest, our findings reveal no association between the age at migraine onset or sex and the efficacy of erenumab, aligning well with existing literature.^7,8,36,37^

Concerning headache characteristics, we observed an independent association between unilateral headache and favourable treatment responses, in line with previous observations for CGRP-monoclonal antibodies and onabotulinumtoxinA.^10,17,48^ In terms of comorbidities, hypertension was associated with a reduced likelihood of achieving a ≥ 50% reduction in MMDs or moderate-severe MHDs. This relationship was observed in only one measured outcome, thus necessitating further investigation to confirm its significance. We observed no association between overweight or obesity and poor or late response, in contrast to some previous reports.^10,49^ Also notably, we observed no association between response to erenumab and symptoms of anxiety or depression.

### Implications for current clinical practice

Setting realistic treatment goals is essential in migraine management,^29,50^ and clinicians should communicate these goals clearly to promote adherence. While a ≥ 50% reduction in MMDs is often the primary goal, this may be unrealistic for patients with chronic migraine, daily headache or those who have failed multiple preventive treatments. For these difficult-to-treat cases, combination therapy might be necessary. If combination therapy is not practical or preferred, a ≥ 30% reduction in MMDs should be considered as a more attainable target. Our results show that about one-fifth of patients with these severe phenotypes achieved a 30–49% reduction in MMDs, suggesting that this may be an acceptable goal. However, further studies with larger sample sizes are needed to confirm whether a 30–49% reduction in MMDs does in fact reflect a clinically meaningful improvement.

Previous real-world studies on CGRP-mAbs suggest that efficacy assessed at 12 weeks may not reliably predict longer-term responses, including those at 24 weeks or 12 months.^14,49^ Our *post hoc* analysis suggests that extending efficacy assessments to week 16 might be sufficient to allow the identification of the majority of responders to erenumab in clinical practice. This may especially be relevant for patients with chronic migraine, high migraine-related disability, or cutaneous allodynia, whom are less likely to experience the full benefits of erenumab within the first 12 weeks of treatment. Thus, a 16-week trial period, assuming no intolerable side effects, may better accommodate individual response patterns and prevent premature treatment discontinuation; however, further studies are needed to validate this extension.

### Limitations

The present study enroled a large sample of participants who all received the same drug and dose. However, certain limitations warrant mention. The sample size was based on REFORM study enrolment, not population-based, which may limit generalizability. Additionally, some relevant clinical predictors may have been missed, and some participant characteristics, such as obesity, were uncommon. Some participants were excluded from the analyses, of which most were considered ineligible due to incomplete headache diary data, for which greater data completion may have been achieved using electronic headache diaries.^51^ Lastly, complete case analysis was used, as multiple imputation was considered inappropriate.^31^

### Future perspectives

Overall, the multivariable models presented were only somewhat helpful at predicting response to erenumab. Enhancing the prediction of therapeutic outcomes might be achieved by integrating clinical data with genetic, biochemical and neuroimaging information.^52^ Use of machine learning could be particularly beneficial for this purpose, offering a promising tool for analysing large complex datasets and advancing personalized medicine.^53^

## Conclusion

In adults with migraines, key clinical factors influencing the response to erenumab treatment include the presence of chronic migraines, daily headache, a higher number of MHDs and prior failure of multiple preventive medications. Tailored approaches, including strategies like combination therapies or adjusted treatment goals, should be considered to optimize outcomes for the most severely affected patients. Moreover, our results highlight that patients with more severe clinical profiles may require longer to respond, underscoring the importance of managing expectations in this population. Further research is needed to establish the optimal timepoint for evaluating efficacy of erenumab, and to integrate clinical and biomarker data to enhance predictive accuracy.

## Supplementary Material

fcaf147_Supplementary_Data

## Data Availability

The data that support the findings of this study are available from the corresponding author, upon reasonable request. The codes generated and used in this work are freely available online at GitHub (https://github.com/WillKarlsson/ClinPredMigraine).
