# Supplementary material for: Clinical predictors for efficacy of erenumab for migraine: a Registry for Migraine (REFORM) study
Source: Brain Commun. 2025 Apr 15;7(2):fcaf147. doi: 10.1093/braincomms/fcaf147 (PMC12015094; doi:10.1093/braincomms/fcaf147)
Supplement: fcaf147_Supplementary_Data [file fcaf147_supplementary_data.docx]

**CLINICAL PREDICTORS FOR EFFICACY OF ERENUMAB FOR MIGRAINE**

**Supplementary Material**

**Table of Contents**

[Supplementary Material 1. Study methodology details 4](#_Toc195133472)

[1.1 Headache diaries 4](#_Toc195133473)

[1.2 Classification of variables 4](#_Toc195133474)

[1.3 Evaluation of model performance 5](#_Toc195133475)

[1.4 Post-hoc analyses 6](#_Toc195133476)

[1.5 Multicollinearity in logistic regression models 6](#_Toc195133477)

[1.6 Additional details 6](#_Toc195133478)

[Supplementary Material 2. Univariate logistic regression models 8](#_Toc195133479)

[2.1 Primary outcome: ≥50% reduction in MMDs 8](#_Toc195133480)

[2.2 Secondary outcomes: ≥50% reduction in MHDs 12](#_Toc195133481)

[2.3 Secondary outcomes: ≥50% reduction in MMDs or moderate-severe MHDs 16](#_Toc195133482)

[2.4 Exploratory outcomes: ≥50% reduction in moderate-severe MHDs 20](#_Toc195133483)

[2.5 Exploratory outcomes: Reduction in MIDAS 24](#_Toc195133484)

[2.6 Exploratory outcomes: ≥5-point reduction in HIT-6 29](#_Toc195133485)

[Supplementary Material 3. Multivariate logistic regression models 33](#_Toc195133486)

[3.1 Primary outcome: ≥50% reduction in MMDs 33](#_Toc195133487)

[3.2 Secondary outcomes: ≥50% reduction in MHDs 34](#_Toc195133488)

[3.3 Secondary outcomes: ≥50% reduction in MMDs or moderate-severe MHDs 35](#_Toc195133489)

[3.4 Exploratory outcomes: ≥50% reduction in moderate-severe MHDs 36](#_Toc195133490)

[3.5 Exploratory outcomes: Reduction in MIDAS 37](#_Toc195133491)

[3.6 Exploratory outcomes: ≥5-point reduction in HIT-6 38](#_Toc195133492)

[Supplementary Material 4. Additional logistic regression analyses 39](#_Toc195133493)

[4.1 Primary outcome: ≥50% reduction in MMDs (disease duration) 39](#_Toc195133494)

[4.2 Primary outcome: ≥50% reduction in MMDs (preventive medication class failures) 40](#_Toc195133495)

[4.3 Primary outcome: ≥50% reduction in MMDs (overweight) 41](#_Toc195133496)

[4.4 Primary outcome: ≥50% reduction in MMDs (inclusion of drop-outs due to adverse events) 42](#_Toc195133497)

[ROC curves 52](#_Toc195133498)

[Supplementary Fig. 1. ROC curve: Primary outcome: ≥50% reduction in MMDs 52](#_Toc195133499)

[Supplementary Fig. 2. ROC curve: Secondary outcomes: ≥50% reduction in MHDs 53](#_Toc195133500)

[Supplementary Fig. 3. ROC curve: Secondary outcomes: ≥50% reduction in MMDs or moderate-severe MHDs 54](#_Toc195133501)

[Supplementary Fig. 4. ROC curve: Exploratory outcomes: ≥50% reduction in moderate-severe MHDs 55](#_Toc195133502)

[Supplementary Fig. 5. ROC curve: Exploratory outcomes: Reduction in MIDAS 56](#_Toc195133503)

[Supplementary Fig. 6. ROC curve: Exploratory outcomes: ≥5-point reduction in HIT-6 57](#_Toc195133504)

[Calibration curves 58](#_Toc195133505)

[Supplementary Fig. 7. Calibration curve: Primary outcome: ≥50% reduction in MMDs 58](#_Toc195133506)

[Supplementary Fig. 8. Calibration curve: Secondary outcomes: ≥50% reduction in MHDs 59](#_Toc195133507)

[Supplementary Fig. 9. Calibration curve: Secondary outcomes: ≥50% reduction in MMDs or moderate-severe MHDs 60](#_Toc195133508)

[Supplementary Fig. 10. Calibration curve: Exploratory outcomes: ≥50% reduction in moderate-severe MHDs 61](#_Toc195133509)

[Supplementary Fig. 11. Calibration curve: Exploratory outcomes: Reduction in MIDAS 62](#_Toc195133510)

[Supplementary Fig. 12. Calibration curve: Exploratory outcomes: ≥5-point reduction in HIT-6 63](#_Toc195133511)

[Supplementary Tables. 64](#_Toc195133512)

[Supplementary Table 1. Low- and high-frequency episodic migraine (baseline characteristics) 64](#_Toc195133513)

[Supplementary Table 2. Low- and high-frequency episodic migraine (attack features) 65](#_Toc195133514)

[Supplementary Table 3. Preventive drug class failures 66](#_Toc195133515)

[Supplementary Table 4. Performance metrics for the multivariable models 67](#_Toc195133516)

[Supplementary Table 5. Participants with outcome data 68](#_Toc195133517)

[Supplementary Table 6. Participants with missing baseline data 69](#_Toc195133518)

[Supplementary Table 7. Participants with missing headache data 70](#_Toc195133519)

[Supplementary Table 8. Key predictive characteristics according to response 71](#_Toc195133520)

[Supplementary Table 9. Absolute response in partial responders 72](#_Toc195133521)

[Supplementary Table 10. Early and late responders (baseline characteristics) 73](#_Toc195133522)

[Supplementary Table 11. Early and late responders (attack features) 74](#_Toc195133523)

Supplementary Material 1. Study methodology details

1.1 Headache diaries

During the baseline period, participants kept a detailed diary that allowed classification of migraines and tension-type headaches based on the ICHD-3 criteria. This included information on headache presence, duration, location, severity, associated symptoms (such as nausea, vomiting, light and sound sensitivity), worsening with physical activity, medication use, and presence of aura. During the 24-week treatment period, a simplified diary was provided, capturing the presence of headache and migraine, aura, and medication use. Oral and written instructions were provided to help participants distinguish between migraine and tension-type headaches.

1.2 Classification of variables

We defined a month as 4 weeks (i.e., 28 days) and a migraine day as any day with self-reported migraine or use of acute migraine medication, such as ergotamine derivatives, lasmiditan, gepants, or triptans. The diagnoses of migraine without aura, migraine with aura, and chronic migraine (episodic in the absence thereof) were ascertained at enrollment according to the ICHD-3 criteria (2018, Headache Classification Committee of the International Headache Society).

The presence of daily headache was defined as participants who recorded having headache every day during the 4-week baseline period. Identification of somatic comorbidities was based on participant self-report of a physician diagnosis and review of medical records. In terms of psychiatric comorbidities, a possible or probable risk of anxiety was determined by a HADS anxiety score of ≥8 (2003, Snaith *et al*). Likewise, a possible or probable risk of depression was defined using a HADS depression score of ≥8 (2003, Snaith *et al*).

Medication-overuse was defined as ≥15 days per month using simple analgesics, or ≥10 days per month using combination-analgesics, ergotamine, opioids, or triptans, within the last 3 months prior to enrollment (2018, Headache Classification Committee of the International Headache Society). Failure of preventive migraine medication(s) was determined based on lack of efficacy, assuming the treatment met the minimum effective doses and durations as per European Headache Federation (EHF) guidelines (2020, Sacco *et al*). Failure due to lack of efficacy or tolerability for each drug class was also noted (2020, Sacco *et al*). Triptan resistance was defined as the failure of at least two triptans, owing to inefficacy. Moreover, unilateral cranial autonomic features were assessed and included conjunctival injection, lacrimation, nasal congestion, rhinorrhea, eyelid oedema, facial or eyelid redness, forehead and facial sweating, miosis, or ptosis.

1.3 Evaluation of model performance

We used both discriminative ability and calibration to evaluate the predictive performance of the multivariable models for each outcome. The discriminative ability was measured using receiver operating characteristic (ROC) curves to compute the area under the curve (AUC). Youden’s J statistic was used to identify the optimal threshold, from which we derived key diagnostic measures, including accuracy, sensitivity, and specificity for each outcome. Confidence intervals (95% CIs) were calculated using bootstrapping with 2,000 resamples, stratified by the ratio of responders to non-responders. To assess calibration of our predictive models, we examined the concordance between predicted and observed outcomes through visual inspection of calibration plots and the Hosmer-Lemeshow test. Models yielding a *p*-value >0.05 on this test were considered adequately calibrated. To evaluate the stability of our prediction models, internal validation was performed using bootstrap resampling. Specifically, 2,000 bootstrap samples were generated by drawing with replacement. Each of these samples was used to fit the model anew. The AUC (*C*-statistics) were then adjusted for optimism by comparing the bootstrap AUC to the original AUC. This adjustment provides a more accurate measure of the models’ predictive performance by accounting for any overfitting that might have occurred in the initial analysis.

For binary clinical predictors significantly associated with the primary outcome, we post-hoc analyzed participant proportions stratified by treatment response: ≥50%, 30–49%, and <30%. For those with a partial response (30–49%), we provided detailed descriptions of the absolute change in monthly migraine days (MMDs), monthly headache days (MHDs), moderate-to-severe MHDs, and days with acute headache medication use. Furthermore, we detailed the proportions of partial responders achieving clinically meaningful improvements in Migraine Disability Assessment Score (MIDAS) and Headache Impact Test-6 (HIT-6) scores.

1.4 Post-hoc analyses

Post-hoc analyses were also performed to compare early responders and late responders in terms of patterns of response and differences in clinical characteristics. Early responders were defined as participants who achieved a reduction from baseline of ≥50% in average MMDs across both weeks 1 through 12 and weeks 13 through 24. Late responders were classified as participants who achieved this reduction only in the final 12 weeks of treatment, i.e., weeks 13 through 24. Descriptive statistics were used to compare these two groups. Furthermore, a mixed model for repeated measure was applied to assess the mean absolute change in MMDs between groups. An autoregressive first-order structure was specified for the correlation between repeated measures over time, with subject-level variability modeled through a random intercept. Further, we compared confusion matrices for response during weeks 13 to 24 with those from weeks 1 to 12, 5 to 16, and 9 to 20. For each comparison, we used weeks 13 to 24 as the reference period and performed McNemar’s test to assess statistical significance.

1.5 Multicollinearity in logistic regression models

Multicollinearity was assessed using variance inflation factors (values < 5 were accepted). Given the overlap between commonly used covariates describing headache and migraine frequency, we considered the multivariate model including chronic migraine our main model, reporting estimates with 95% CIs of the covariates from this model in the manuscript. We ran separate multivariable models for (I) daily headache, and (II) MHD and MMD, replacing chronic migraine in these models and extracted only estimates with 95% CIs specifically for these variables. To further account for collinearity, separate models were run for age and migraine duration, as well as for the number of preventive medications failed and preventive drug class failures.

1.6 Additional details

For age, we reported the OR per 10-year increase to facilitate interpretation of the effect size.

Differences in baseline clinical characteristics were compared between participants with chronic migraine, high-frequency episodic migraine, and low-frequency episodic migraine using analysis of variance (ANOVA) or Kruskal-Wallis’ test according to distribution for continuous variables, and Fisher’s exact test or Chi-square test as appropriate for categorical variables.

**Supplementary references**

Headache Classification Committee of the International Headache Society (IHS). The International Classification of Headache Disorders, 3rd edition. *Cephalalgia*. 2018;38(1):1-211. doi:10.1177/0333102417738202

Sacco S, Braschinsky M, Ducros A, et al. European headache federation consensus on the definition of resistant and refractory migraine. *Journal of Headache and Pain*. 2020;21(1). doi:10.1186/s10194-020-01130-5

Snaith RP. The Hospital Anxiety And Depression Scale. *Health and Quality of Life Outcomes*. 2003;1(29). doi:10.1186/1477-7525-1-29

Supplementary Material 2. Univariate logistic regression models

2.1 Primary outcome: ≥50% reduction in MMDs

| **Characteristic** | **N** | **Event N** | **OR***^1^* | **95% CI***^1^* | **p-value** |
| --- | --- | --- | --- | --- | --- |
| **Age, 10-year increase** | 570 | 298 | 1.21 | 1.06, 1.39 | 0.006 |
| **Sex** | 570 | 298 |  |  | 0.230 |
| Male |  |  | — | — |  |
| Female |  |  | 0.71 | 0.40, 1.24 |  |
| **Obesity (≥30 kg/m2)** | 570 | 298 |  |  | 0.052 |
| No |  |  | — | — |  |
| Yes |  |  | 0.64 | 0.41, 1.00 |  |
| **Age at onset, years** | 570 | 298 | 0.99 | 0.98, 1.01 | 0.298 |
| **Migraine disease duration, 1-year increase** | 570 | 298 | 1.02 | 1.01, 1.03 | <0.001 |
| **First degree relative with migraine** | 570 | 298 |  |  | 0.634 |
| No |  |  | — | — |  |
| Yes |  |  | 0.92 | 0.64, 1.31 |  |
| **Migraine with aura** | 570 | 298 |  |  | 0.878 |
| No |  |  | — | — |  |
| Yes |  |  | 0.97 | 0.68, 1.40 |  |
| **Chronic migraine** | 570 | 298 |  |  | 0.014 |
| EM |  |  | — | — |  |
| CM |  |  | 0.64 | 0.45, 0.91 |  |
| **Daily headache (28-day baseline)** | 570 | 298 |  |  | <0.001 |
| No |  |  | — | — |  |
| Yes |  |  | 0.40 | 0.24, 0.63 |  |
| **Medication-overuse** | 570 | 298 |  |  | 0.541 |
| No |  |  | — | — |  |
| Yes |  |  | 1.11 | 0.80, 1.55 |  |
| **Monthly headache days (baseline), 1-day increase** | 570 | 298 | 0.97 | 0.94, 0.99 | 0.006 |
| **Monthly migraine days (baseline), 1-day increase** | 570 | 298 | 1.00 | 0.97, 1.03 | 0.963 |
| **Monthly days with use of acute medications (baseline), 1-day increase** | 570 | 298 | 0.99 | 0.96, 1.02 | 0.424 |
| **Ongoing use of preventive medications** | 570 | 298 |  |  | 0.389 |
| No |  |  | — | — |  |
| Yes |  |  | 1.16 | 0.83, 1.61 |  |
| **≥2 or more triptans discontinued due to lack of efficacy (triptan resistance)** | 543 | 282 |  |  | 0.130 |
| No |  |  | — | — |  |
| Yes |  |  | 0.73 | 0.48, 1.10 |  |
| **No. of preventive medications discontinued due to lack of efficacy** | 570 | 298 |  |  | <0.001 |
| <3 |  |  | — | — |  |
| ≥3 |  |  | 0.53 | 0.37, 0.76 |  |
| **No. of preventive drug class failures** | 570 | 298 |  |  | 0.006 |
| <3 |  |  | — | — |  |
| ≥3 |  |  | 0.63 | 0.45, 0.87 |  |
| **MIDAS score (baseline), 1-point increase** | 552 | 287 | 1.00 | 1.00, 1.00 | 0.623 |
| **HIT6 score (baseline), 1-point increase** | 555 | 289 | 1.03 | 1.0, 1.08 | 0.091 |
| **WHODAS score (baseline), 1-point increase** | 547 | 284 | 1.01 | 0.99, 1.03 | 0.421 |
| **Asthma** | 570 | 298 |  |  | 0.458 |
| No |  |  | — | — |  |
| Yes |  |  | 1.23 | 0.71, 2.15 |  |
| **Autoimmunologic conditions** | 570 | 298 |  |  | 0.272 |
| No |  |  | — | — |  |
| Yes |  |  | 1.32 | 0.81, 2.20 |  |
| **Constipation** | 570 | 298 | 1.23 | 0.80, 1.90 | 0.357 |
| **Chronic neck pain (≥3 months)** | 570 | 298 |  |  | 0.118 |
| No |  |  | — | — |  |
| Yes |  |  | 1.46 | 0.91, 2.36 |  |
| **Chronic back pain (≥3 months)** | 570 | 298 |  |  | 0.343 |
| No |  |  | — | — |  |
| Yes |  |  | 1.32 | 0.75, 2.37 |  |
| **Hypertension** | 570 | 298 |  |  | 0.186 |
| No |  |  | — | — |  |
| Yes |  |  | 0.70 | 0.41, 1.19 |  |
| **HADS-D ≥8 (possible or probable risk of depression)** | 552 | 287 |  |  | 0.307 |
| Normal |  |  | — | — |  |
| Possible or probable depression |  |  | 0.81 | 0.55, 1.21 |  |
| **HADS-A ≥8 (possible or probable risk of anxiety)** | 552 | 287 |  |  | 0.796 |
| Normal |  |  | — | — |  |
| Possible or probable anxiety |  |  | 1.05 | 0.73, 1.52 |  |
| **Unilateral headache** | 570 | 298 |  |  | 0.420 |
| Bilateral |  |  | — | — |  |
| Unilateral |  |  | 1.21 | 0.76, 1.93 |  |
| **Side-locked headache** | 570 | 298 |  |  | 0.741 |
| Not side-locked |  |  | — | — |  |
| Side-locked |  |  | 0.92 | 0.58, 1.47 |  |
| **Headache intensity (4-point scale), 1-point increase** | 570 | 298 |  |  | 0.682 |
| Mild |  |  | — | — |  |
| Moderate |  |  | 2.04 | 0.19, 44.5 |  |
| Severe |  |  | 2.28 | 0.22, 49.2 |  |
| **Pain quality: Pulsating** | 570 | 298 |  |  | 0.929 |
| No |  |  | — | — |  |
| Yes |  |  | 1.02 | 0.70, 1.47 |  |
| **Pain aggravation upon physical exercise** | 570 | 298 |  |  | 0.130 |
| No |  |  | — | — |  |
| Yes |  |  | 1.57 | 0.88, 2.86 |  |
| **Photophobia** | 570 | 298 |  |  | 0.812 |
| No |  |  | — | — |  |
| Yes |  |  | 1.10 | 0.50, 2.44 |  |
| **Phonophobia** | 570 | 298 |  |  | 0.967 |
| No |  |  | — | — |  |
| Yes |  |  | 1.01 | 0.56, 1.81 |  |
| **Nausea** | 570 | 298 |  |  | 0.127 |
| No |  |  | — | — |  |
| Yes |  |  | 1.60 | 0.88, 2.94 |  |
| **Vomiting** | 570 | 298 |  |  | 0.269 |
| No |  |  | — | — |  |
| Yes |  |  | 1.21 | 0.87, 1.68 |  |
| **Unilateral autonomic symptoms** | 566 | 294 |  |  | 0.631 |
| None |  |  | — | — |  |
| Present |  |  | 0.91 | 0.60, 1.36 |  |
| **ASC-12 score (baseline), 1-point increase** | 549 | 285 | 1.03 | 1.0, 1.07 | 0.096 |
| *^1^* OR = Odds Ratio, CI = Confidence Interval | | | | | |

2.2 Secondary outcomes: ≥50% reduction in MHDs

| **Characteristic** | **N** | **Event N** | **OR***^1^* | **95% CI***^1^* | **p-value** |
| --- | --- | --- | --- | --- | --- |
| **Age, 10-year increase** | 570 | 239 | 1.23 | 1.07, 1.41 | 0.004 |
| **Sex** | 570 | 239 |  |  | 0.578 |
| Male |  |  | — | — |  |
| Female |  |  | 0.85 | 0.49, 1.50 |  |
| **Obesity (≥30 kg/m2)** | 570 | 239 |  |  | 0.647 |
| No |  |  | — | — |  |
| Yes |  |  | 0.90 | 0.57, 1.41 |  |
| **Age at onset, years** | 570 | 239 | 1.00 | 0.99, 1.02 | 0.805 |
| **Migraine disease duration, 1-year increase** | 570 | 239 | 1.02 | 1.00, 1.03 | 0.017 |
| **First degree relative with migraine** | 570 | 239 |  |  | 0.810 |
| No |  |  | — | — |  |
| Yes |  |  | 0.96 | 0.67, 1.38 |  |
| **Migraine with aura** | 570 | 239 |  |  | 0.408 |
| No |  |  | — | — |  |
| Yes |  |  | 0.86 | 0.59, 1.23 |  |
| **Chronic migraine** | 570 | 239 |  |  | <0.001 |
| EM |  |  | — | — |  |
| CM |  |  | 0.45 | 0.31, 0.63 |  |
| **Daily headache (28-day baseline)** | 570 | 239 |  |  | <0.001 |
| No |  |  | — | — |  |
| Yes |  |  | 0.19 | 0.10, 0.34 |  |
| **Medication-overuse** | 570 | 239 |  |  | 0.980 |
| No |  |  | — | — |  |
| Yes |  |  | 1.00 | 0.72, 1.41 |  |
| **Monthly headache days (baseline), 1-day increase** | 570 | 239 | 0.92 | 0.90, 0.95 | <0.001 |
| **Monthly migraine days (baseline), 1-day increase** | 570 | 239 | 0.98 | 0.95, 1.01 | 0.196 |
| **Monthly days with use of acute medications (baseline), 1-day increase** | 570 | 239 | 0.97 | 0.94, 1.00 | 0.098 |
| **Ongoing use of preventive medications** | 570 | 239 |  |  | 0.732 |
| No |  |  | — | — |  |
| Yes |  |  | 1.06 | 0.76, 1.48 |  |
| **≥2 or more triptans discontinued due to lack of efficacy (triptan resistance)** | 543 | 226 |  |  | 0.134 |
| No |  |  | — | — |  |
| Yes |  |  | 0.72 | 0.47, 1.10 |  |
| **No. of preventive medications discontinued due to lack of efficacy** | 570 | 239 |  |  | 0.010 |
| <3 |  |  | — | — |  |
| ≥3 |  |  | 0.62 | 0.43, 0.89 |  |
| **No. of preventive drug class failures** | 570 | 239 |  |  | 0.004 |
| <3 |  |  | — | — |  |
| ≥3 |  |  | 0.61 | 0.43, 0.85 |  |
| **MIDAS score (baseline), 1-point increase** | 552 | 229 | 1.00 | 0.99, 1.00 | 0.035 |
| **HIT6 score (baseline), 1-point increase** | 555 | 231 | 1.02 | 0.98, 1.06 | 0.333 |
| **WHODAS score (baseline), 1-point increase** | 547 | 226 | 0.99 | 0.97, 1.01 | 0.363 |
| **Asthma** | 570 | 239 |  |  | 0.929 |
| No |  |  | — | — |  |
| Yes |  |  | 0.98 | 0.56, 1.68 |  |
| **Autoimmunologic conditions** | 570 | 239 |  |  | 0.961 |
| No |  |  | — | — |  |
| Yes |  |  | 0.99 | 0.59, 1.62 |  |
| **Constipation** | 570 | 239 | 1.03 | 0.67, 1.59 | 0.885 |
| **Chronic neck pain (≥3 months)** | 570 | 239 |  |  | 0.103 |
| No |  |  | — | — |  |
| Yes |  |  | 0.67 | 0.40, 1.08 |  |
| **Chronic back pain (≥3 months)** | 570 | 239 |  |  | 0.219 |
| No |  |  | — | — |  |
| Yes |  |  | 0.69 | 0.37, 1.23 |  |
| **Hypertension** | 570 | 239 |  |  | 0.211 |
| No |  |  | — | — |  |
| Yes |  |  | 0.70 | 0.40, 1.21 |  |
| **HADS-D ≥8 (possible or probable risk of depression)** | 552 | 229 |  |  | 0.091 |
| Normal |  |  | — | — |  |
| Possible or probable depression |  |  | 0.70 | 0.47, 1.05 |  |
| **HADS-A ≥8 (possible or probable risk of anxiety)** | 552 | 229 |  |  | 0.683 |
| Normal |  |  | — | — |  |
| Possible or probable anxiety |  |  | 0.92 | 0.63, 1.35 |  |
| **Unilateral headache** | 570 | 239 |  |  | 0.005 |
| Bilateral |  |  | — | — |  |
| Unilateral |  |  | 2.08 | 1.26, 3.53 |  |
| **Side-locked headache** | 570 | 239 |  |  | 0.665 |
| Not side-locked |  |  | — | — |  |
| Side-locked |  |  | 0.90 | 0.56, 1.44 |  |
| **Headache intensity (4-point scale), 1-point increase** | 570 | 239 |  |  | 0.951 |
| Mild |  |  | — | — |  |
| Moderate |  |  | 1.43 | 0.13, 31.1 |  |
| Severe |  |  | 1.46 | 0.14, 31.5 |  |
| **Pain quality: Pulsating** | 570 | 239 |  |  | 0.959 |
| No |  |  | — | — |  |
| Yes |  |  | 0.99 | 0.68, 1.45 |  |
| **Pain aggravation upon physical exercise** | 570 | 239 |  |  | 0.992 |
| No |  |  | — | — |  |
| Yes |  |  | 1.00 | 0.56, 1.81 |  |
| **Photophobia** | 570 | 239 |  |  | 0.714 |
| No |  |  | — | — |  |
| Yes |  |  | 1.16 | 0.53, 2.70 |  |
| **Phonophobia** | 570 | 239 |  |  | 0.756 |
| No |  |  | — | — |  |
| Yes |  |  | 0.91 | 0.51, 1.65 |  |
| **Nausea** | 570 | 239 |  |  | 0.032 |
| No |  |  | — | — |  |
| Yes |  |  | 2.06 | 1.09, 4.12 |  |
| **Vomiting** | 570 | 239 |  |  | 0.146 |
| No |  |  | — | — |  |
| Yes |  |  | 1.29 | 0.92, 1.81 |  |
| **Unilateral autonomic symptoms** | 566 | 238 |  |  | 0.748 |
| None |  |  | — | — |  |
| Present |  |  | 1.07 | 0.71, 1.60 |  |
| **ASC-12 score (baseline), 1-point increase** | 549 | 227 | 1.02 | 0.98, 1.06 | 0.346 |
| *^1^* OR = Odds Ratio, CI = Confidence Interval | | | | | |

2.3 Secondary outcomes: ≥50% reduction in MMDs or moderate-severe MHDs

| **Characteristic** | **N** | **Event N** | **OR***^1^* | **95% CI***^1^* | **p-value** |
| --- | --- | --- | --- | --- | --- |
| **Age, 10-year increase** | 568 | 358 | 1.34 | 1.16, 1.55 | <0.001 |
| **Sex** | 568 | 358 |  |  | 0.205 |
| Male |  |  | — | — |  |
| Female |  |  | 0.67 | 0.36, 1.22 |  |
| **Obese (≥30 kg/m2)** | 568 | 358 |  |  | 0.061 |
| Not obese |  |  | — | — |  |
| Obese |  |  | 0.65 | 0.41, 1.02 |  |
| **Age at onset, years** | 568 | 358 | 1.00 | 0.98, 1.01 | 0.839 |
| **Migraine disease duration, 1-year increase** | 568 | 358 | 1.03 | 1.01, 1.04 | <0.001 |
| **First degree relative with migraine** | 568 | 358 |  |  | 0.155 |
| No |  |  | — | — |  |
| Yes |  |  | 0.76 | 0.52, 1.11 |  |
| **Migraine with aura** | 568 | 358 |  |  | 0.793 |
| No |  |  | — | — |  |
| Yes |  |  | 1.05 | 0.72, 1.54 |  |
| **Chronic migraine** | 568 | 358 |  |  | <0.001 |
| EM |  |  | — | — |  |
| CM |  |  | 0.52 | 0.36, 0.75 |  |
| **Daily headache (28-day baseline)** | 567 | 357 |  |  | <0.001 |
| No |  |  | — | — |  |
| Yes |  |  | 0.27 | 0.17, 0.43 |  |
| **Medication-overuse headache** | 568 | 358 |  |  | 0.593 |
| No |  |  | — | — |  |
| Yes |  |  | 0.91 | 0.64, 1.30 |  |
| **Monthly headache days (baseline), 1-day increase** | 568 | 358 | 0.94 | 0.92, 0.97 | <0.001 |
| **Monthly migraine days (baseline), 1-day increase** | 568 | 358 | 0.98 | 0.96, 1.01 | 0.297 |
| **Monthly days with use of acute medications (baseline), 1-day increase** | 568 | 358 | 1.00 | 0.97, 1.04 | 0.830 |
| **Ongoing use of preventive medications** | 568 | 358 |  |  | 0.892 |
| No |  |  | — | — |  |
| Yes |  |  | 1.02 | 0.73, 1.44 |  |
| **≥2 or more triptans discontinued due to lack of efficacy (triptan resistance)** | 541 | 341 |  |  | 0.553 |
| No |  |  | — | — |  |
| Yes |  |  | 0.88 | 0.58, 1.35 |  |
| **No. of preventive medications discontinued due to lack of efficacy** | 568 | 358 |  |  | 0.003 |
| <3 |  |  | — | — |  |
| ≥3 |  |  | 0.58 | 0.41, 0.83 |  |
| **No. of preventive drug class failures** | 568 | 358 |  |  | 0.006 |
| <3 |  |  | — | — |  |
| ≥3 |  |  | 0.62 | 0.44, 0.87 |  |
| **MIDAS score (baseline), 1-point increase** | 550 | 346 | 1.00 | 1.00, 1.00 | 0.077 |
| **HIT6 score (baseline), 1-point increase** | 553 | 348 | 1.01 | 0.97, 1.05 | 0.600 |
| **WHODAS score (baseline), 1-point increase** | 545 | 342 | 0.99 | 0.97, 1.02 | 0.564 |
| **Asthma** | 568 | 358 |  |  | 0.899 |
| No |  |  | — | — |  |
| Yes |  |  | 1.04 | 0.60, 1.85 |  |
| **Autoimmunologic conditions** | 568 | 358 |  |  | 0.672 |
| No |  |  | — | — |  |
| Yes |  |  | 1.12 | 0.67, 1.90 |  |
| **Constipation** | 568 | 358 | 1.07 | 0.69, 1.69 | 0.760 |
| **Chronic neck pain (≥3 months)** | 568 | 358 |  |  | 0.509 |
| No |  |  | — | — |  |
| Yes |  |  | 1.18 | 0.73, 1.95 |  |
| **Chronic back pain (≥3 months)** | 568 | 358 |  |  | 0.859 |
| No |  |  | — | — |  |
| Yes |  |  | 1.05 | 0.59, 1.93 |  |
| **Hypertension** | 568 | 358 |  |  | 0.128 |
| No |  |  | — | — |  |
| Yes |  |  | 0.66 | 0.39, 1.13 |  |
| **HADS-D ≥8 (possible or probable risk of depression)** | 550 | 346 |  |  | 0.159 |
| Not possible depression |  |  | — | — |  |
| Possible or probable depression |  |  | 0.75 | 0.50, 1.12 |  |
| **HADS-A ≥8 (possible or probable risk of anxiety)** | 550 | 346 |  |  | 0.315 |
| Not possible anxiety |  |  | — | — |  |
| Possible or probable anxiety |  |  | 0.82 | 0.56, 1.21 |  |
| **Unilateral headache** | 568 | 358 |  |  | 0.415 |
| Bilateral |  |  | — | — |  |
| Unilateral |  |  | 1.22 | 0.75, 1.95 |  |
| **Side-locked headache** | 568 | 358 |  |  | 0.509 |
| Not side-locked |  |  | — | — |  |
| Side-locked |  |  | 1.18 | 0.73, 1.95 |  |
| **Headache intensity (4-point scale), 1-point increase** | 568 | 358 |  |  | 0.501 |
| Mild |  |  | — | — |  |
| Moderate |  |  | 3.71 | 0.35, 80.8 |  |
| Severe |  |  | 3.31 | 0.31, 71.6 |  |
| **Pain quality: Pulsating** | 568 | 358 |  |  | 0.875 |
| No |  |  | — | — |  |
| Yes |  |  | 1.03 | 0.70, 1.51 |  |
| **Pain aggravation upon physical exercise** | 568 | 358 |  |  | 0.168 |
| No |  |  | — | — |  |
| Yes |  |  | 1.51 | 0.83, 2.70 |  |
| **Photophobia** | 568 | 358 |  |  | 0.872 |
| No |  |  | — | — |  |
| Yes |  |  | 1.07 | 0.46, 2.37 |  |
| **Phonophobia** | 568 | 358 |  |  | 0.441 |
| No |  |  | — | — |  |
| Yes |  |  | 1.26 | 0.69, 2.26 |  |
| **Nausea** | 568 | 358 |  |  | 0.103 |
| No |  |  | — | — |  |
| Yes |  |  | 1.64 | 0.90, 2.97 |  |
| **Vomiting** | 568 | 358 |  |  | 0.251 |
| No |  |  | — | — |  |
| Yes |  |  | 1.22 | 0.87, 1.72 |  |
| **Unilateral autonomic symptoms** | 564 | 354 |  |  | 0.480 |
| None |  |  | — | — |  |
| Present |  |  | 1.16 | 0.77, 1.79 |  |
| **ASC-12 score (baseline), 1-point increase** | 547 | 343 | 1.02 | 0.98, 1.06 | 0.433 |

2.4 Exploratory outcomes: ≥50% reduction in moderate-severe MHDs

| **Characteristic** | **N** | **Event N** | **OR***^1^* | **95% CI***^1^* | **p-value** |
| --- | --- | --- | --- | --- | --- |
| **Age, 10-year increase** | 570 | 291 | 1.31 | 1.14, 1.51 | <0.001 |
| **Sex** | 570 | 291 |  |  | 0.165 |
| Male |  |  | — | — |  |
| Female |  |  | 0.67 | 0.38, 1.17 |  |
| **Obesity (≥30 kg/m2)** | 570 | 291 |  |  | 0.143 |
| No |  |  | — | — |  |
| Yes |  |  | 0.72 | 0.46, 1.12 |  |
| **Age at onset, years** | 570 | 291 | 1.00 | 0.98, 1.01 | 0.742 |
| **Migraine disease duration, 1-year increase** | 570 | 291 | 1.02 | 1.01, 1.04 | <0.001 |
| **First degree relative with migraine** | 570 | 291 |  |  | 0.956 |
| No |  |  | — | — |  |
| Yes |  |  | 1.01 | 0.71, 1.45 |  |
| **Migraine with aura** | 570 | 291 |  |  | 0.553 |
| No |  |  | — | — |  |
| Yes |  |  | 1.12 | 0.78, 1.60 |  |
| **Chronic migraine** | 570 | 291 |  |  | <0.001 |
| EM |  |  | — | — |  |
| CM |  |  | 0.45 | 0.31, 0.64 |  |
| **Daily headache (28-day baseline)** | 570 | 291 |  |  | <0.001 |
| No |  |  | — | — |  |
| Yes |  |  | 0.29 | 0.17, 0.47 |  |
| **Medication-overuse** | 570 | 291 |  |  | 0.945 |
| No |  |  | — | — |  |
| Yes |  |  | 0.99 | 0.71, 1.38 |  |
| **Monthly headache days (baseline), 1-day increase** | 570 | 291 | 0.94 | 0.91, 0.96 | <0.001 |
| **Monthly migraine days (baseline), 1-day increase** | 570 | 291 | 0.98 | 0.95, 1.00 | 0.083 |
| **Monthly days with use of acute medications (baseline), 1-day increase** | 570 | 291 | 0.98 | 0.95, 1.01 | 0.275 |
| **Ongoing use of preventive medications** | 570 | 291 |  |  | 0.793 |
| No |  |  | — | — |  |
| Yes |  |  | 1.04 | 0.75, 1.45 |  |
| **≥2 or more triptans discontinued due to lack of efficacy (triptan resistance)** | 543 | 278 |  |  | 0.171 |
| No |  |  | — | — |  |
| Yes |  |  | 1.33 | 0.88, 2.02 |  |
| **No. of preventive medications discontinued due to lack of efficacy** | 570 | 291 |  |  | 0.027 |
| <3 |  |  | — | — |  |
| ≥3 |  |  | 0.67 | 0.47, 0.95 |  |
| **No. of preventive drug class failures** | 570 | 291 |  |  | 0.017 |
| <3 |  |  | — | — |  |
| ≥3 |  |  | 0.67 | 0.48, 0.93 |  |
| **MIDAS score (baseline), 1-point increase** | 552 | 281 | 1.00 | 0.99, 1.00 | 0.005 |
| **HIT6 score (baseline), 1-point increase** | 555 | 283 | 0.99 | 0.95, 1.03 | 0.567 |
| **WHODAS score (baseline), 1-point increase** | 547 | 278 | 0.98 | 0.96, 1.00 | 0.114 |
| **Asthma** | 570 | 291 |  |  | 0.914 |
| No |  |  | — | — |  |
| Yes |  |  | 1.03 | 0.60, 1.78 |  |
| **Autoimmunologic conditions** | 570 | 291 |  |  | 0.286 |
| No |  |  | — | — |  |
| Yes |  |  | 1.31 | 0.80, 2.17 |  |
| **Constipation** | 570 | 291 | 0.80 | 0.52, 1.23 | 0.317 |
| **Chronic neck pain (≥3 months)** | 570 | 291 |  |  | 0.573 |
| No |  |  | — | — |  |
| Yes |  |  | 0.87 | 0.55, 1.39 |  |
| **Chronic back pain (≥3 months)** | 570 | 291 |  |  | 0.379 |
| No |  |  | — | — |  |
| Yes |  |  | 0.77 | 0.44, 1.37 |  |
| **Hypertension** | 570 | 291 |  |  | 0.165 |
| No |  |  | — | — |  |
| Yes |  |  | 0.68 | 0.40, 1.17 |  |
| **HADS-D ≥8 (possible or probable risk of depression)** | 552 | 281 |  |  | 0.125 |
| Normal |  |  | — | — |  |
| Possible or probable depression |  |  | 0.73 | 0.49, 1.09 |  |
| **HADS-A ≥8 (possible or probable risk of anxiety)** | 552 | 281 |  |  | 0.264 |
| Normal |  |  | — | — |  |
| Possible or probable anxiety |  |  | 0.81 | 0.56, 1.17 |  |
| **Unilateral headache** | 570 | 291 |  |  | 0.015 |
| Bilateral |  |  | — | — |  |
| Unilateral |  |  | 1.81 | 1.13, 2.94 |  |
| **Side-locked headache** | 570 | 291 |  |  | 0.744 |
| Not side-locked |  |  | — | — |  |
| Side-locked |  |  | 0.93 | 0.58, 1.48 |  |
| **Headache intensity (4-point scale), 1-point increase** | 570 | 291 |  |  | 0.638 |
| Mild |  |  | — | — |  |
| Moderate |  |  | 1.91 | 0.18, 41.6 |  |
| Severe |  |  | 2.18 | 0.21, 47.2 |  |
| **Pain quality: Pulsating** | 570 | 291 |  |  | 0.226 |
| No |  |  | — | — |  |
| Yes |  |  | 0.79 | 0.55, 1.15 |  |
| **Pain aggravation upon physical exercise** | 570 | 291 |  |  | 0.651 |
| No |  |  | — | — |  |
| Yes |  |  | 1.14 | 0.64, 2.05 |  |
| **Photophobia** | 570 | 291 |  |  | 0.277 |
| No |  |  | — | — |  |
| Yes |  |  | 0.64 | 0.28, 1.41 |  |
| **Phonophobia** | 570 | 291 |  |  | 0.058 |
| No |  |  | — | — |  |
| Yes |  |  | 0.56 | 0.30, 1.01 |  |
| **Nausea** | 570 | 291 |  |  | 0.292 |
| No |  |  | — | — |  |
| Yes |  |  | 1.38 | 0.76, 2.52 |  |
| **Vomiting** | 570 | 291 |  |  | 0.201 |
| No |  |  | — | — |  |
| Yes |  |  | 1.24 | 0.89, 1.73 |  |
| **Unilateral autonomic symptoms** | 566 | 289 |  |  | 0.575 |
| None |  |  | — | — |  |
| Present |  |  | 1.12 | 0.75, 1.68 |  |
| **ASC-12 score (baseline), 1-point increase** | 549 | 279 | 1.01 | 0.97, 1.05 | 0.642 |
| *^1^* OR = Odds Ratio, CI = Confidence Interval | | | | | |

2.5 Exploratory outcomes: Reduction in MIDAS

| **Characteristic** | **N** | **Event N** | **OR***^1^* | **95% CI***^1^* | **p-value** |
| --- | --- | --- | --- | --- | --- |
| **Age, 10-year increase** | 487 | 351 | 1.07 | 0.91, 1.26 | 0.423 |
| **Sex** | 487 | 351 |  |  | 0.802 |
| Male |  |  | — | — |  |
| Female |  |  | 1.09 | 0.54, 2.11 |  |
| **Obesity (≥30 kg/m2)** | 487 | 351 |  |  | 0.177 |
| No |  |  | — | — |  |
| Yes |  |  | 0.70 | 0.42, 1.19 |  |
| **Age at onset, years** | 487 | 351 | 0.99 | 0.97, 1.01 | 0.437 |
| **Migraine disease duration, 1-year increase** | 487 | 351 | 1.01 | 1.0, 1.03 | 0.209 |
| **First degree relative with migraine** | 487 | 351 |  |  | 0.913 |
| No |  |  | — | — |  |
| Yes |  |  | 0.98 | 0.63, 1.50 |  |
| **Migraine with aura** | 487 | 351 |  |  | 0.713 |
| No |  |  | — | — |  |
| Yes |  |  | 1.09 | 0.70, 1.70 |  |
| **Chronic migraine** | 487 | 351 |  |  | 0.315 |
| EM |  |  | — | — |  |
| CM |  |  | 0.80 | 0.52, 1.23 |  |
| **Daily headache (28-day baseline)** | 487 | 351 |  |  | <0.001 |
| No |  |  | — | — |  |
| Yes |  |  | 0.34 | 0.21, 0.55 |  |
| **Medication-overuse** | 487 | 351 |  |  | 0.733 |
| No |  |  | — | — |  |
| Yes |  |  | 1.07 | 0.72, 1.59 |  |
| **Monthly headache days (baseline), 1-day increase** | 487 | 351 | 0.94 | 0.91, 0.97 | <0.001 |
| **Monthly migraine days (baseline), 1-day increase** | 487 | 351 | 0.97 | 0.94, 1.00 | 0.069 |
| **Monthly days with use of acute medications (baseline), 1-day increase** | 487 | 351 | 1.00 | 0.96, 1.04 | 0.881 |
| **Ongoing use of preventive medications** | 487 | 351 |  |  | 0.947 |
| No |  |  | — | — |  |
| Yes |  |  | 0.99 | 0.66, 1.47 |  |
| **≥2 or more triptans discontinued due to lack of efficacy (triptan resistance)** | 465 | 335 |  |  | 0.130 |
| No |  |  | — | — |  |
| Yes |  |  | 0.70 | 0.44, 1.12 |  |
| **No. of preventive medications discontinued due to lack of efficacy** | 487 | 351 |  |  | 0.092 |
| <3 |  |  | — | — |  |
| ≥3 |  |  | 0.70 | 0.47, 1.06 |  |
| **No. of preventive drug class failures** | 487 | 351 |  |  | 0.111 |
| <3 |  |  | — | — |  |
| ≥3 |  |  | 0.72 | 0.49, 1.08 |  |
| **MIDAS score (baseline), 1-point increase** | 487 | 351 | 1.00 | 1.00, 1.00 | 0.322 |
| **HIT6 score (baseline), 1-point increase** | 487 | 351 | 0.99 | 0.94, 1.04 | 0.572 |
| **WHODAS score (baseline), 1-point increase** | 484 | 350 | 0.96 | 0.94, 0.99 | 0.004 |
| **Asthma** | 487 | 351 |  |  | 0.182 |
| No |  |  | — | — |  |
| Yes |  |  | 0.66 | 0.36, 1.24 |  |
| **Autoimmunologic conditions** | 487 | 351 |  |  | 0.111 |
| No |  |  | — | — |  |
| Yes |  |  | 1.72 | 0.91, 3.48 |  |
| **Constipation** | 487 | 351 | 1.09 | 0.66, 1.83 | 0.749 |
| **Chronic neck pain (≥3 months)** | 487 | 351 |  |  | 0.565 |
| No |  |  | — | — |  |
| Yes |  |  | 0.85 | 0.50, 1.48 |  |
| **Chronic back pain (≥3 months)** | 487 | 351 |  |  | 0.765 |
| No |  |  | — | — |  |
| Yes |  |  | 0.90 | 0.48, 1.80 |  |
| **Hypertension** | 487 | 351 |  |  | 0.537 |
| No |  |  | — | — |  |
| Yes |  |  | 0.82 | 0.45, 1.55 |  |
| **HADS-D ≥8 (possible or probable risk of depression)** | 487 | 351 |  |  | 0.013 |
| Normal |  |  | — | — |  |
| Possible or probable depression |  |  | 0.58 | 0.37, 0.89 |  |
| **HADS-A ≥8 (possible or probable risk of anxiety)** | 487 | 351 |  |  | 0.894 |
| Normal |  |  | — | — |  |
| Possible or probable anxiety |  |  | 1.03 | 0.67, 1.59 |  |
| **Unilateral headache** | 487 | 351 |  |  | 0.281 |
| Bilateral |  |  | — | — |  |
| Unilateral |  |  | 1.35 | 0.77, 2.31 |  |
| **Side-locked headache** | 487 | 351 |  |  | 0.411 |
| Not side-locked |  |  | — | — |  |
| Side-locked |  |  | 0.80 | 0.47, 1.39 |  |
| **Headache intensity (4-point scale), 1-point increase** | 487 | 351 |  |  | 0.993 |
| Mild |  |  | — | — |  |
| Moderate |  |  | 0.00 |  |  |
| Severe |  |  | 0.00 |  |  |
| **Pain quality: Pulsating** | 487 | 351 |  |  | 0.902 |
| No |  |  | — | — |  |
| Yes |  |  | 1.03 | 0.65, 1.60 |  |
| **Pain aggravation upon physical exercise** | 487 | 351 |  |  | 0.818 |
| No |  |  | — | — |  |
| Yes |  |  | 0.92 | 0.41, 1.88 |  |
| **Photophobia** | 487 | 351 |  |  | 0.180 |
| No |  |  | — | — |  |
| Yes |  |  | 0.36 | 0.06, 1.31 |  |
| **Phonophobia** | 487 | 351 |  |  | 0.243 |
| No |  |  | — | — |  |
| Yes |  |  | 0.60 | 0.24, 1.34 |  |
| **Nausea** | 487 | 351 |  |  | 0.680 |
| No |  |  | — | — |  |
| Yes |  |  | 1.16 | 0.55, 2.31 |  |
| **Vomiting** | 487 | 351 |  |  | 0.914 |
| No |  |  | — | — |  |
| Yes |  |  | 0.98 | 0.65, 1.46 |  |
| **Unilateral autonomic symptoms** | 483 | 347 |  |  | 0.459 |
| None |  |  | — | — |  |
| Present |  |  | 0.84 | 0.52, 1.36 |  |
| **ASC-12 score (baseline), 1-point increase** | 486 | 351 | 1.03 | 0.99, 1.08 | 0.191 |
| *^1^* OR = Odds Ratio, CI = Confidence Interval | | | | | |

2.6 Exploratory outcomes: ≥5-point reduction in HIT-6

| **Characteristic** | **N** | **Event N** | **OR***^1^* | **95% CI***^1^* | **p-value** |
| --- | --- | --- | --- | --- | --- |
| **Age, 10-year increase** | 532 | 267 | 1.02 | 0.89, 1.17 | 0.774 |
| **Sex** | 532 | 267 |  |  | 0.055 |
| Male |  |  | — | — |  |
| Female |  |  | 1.79 | 1.00, 3.30 |  |
| **Obesity (≥30 kg/m2)** | 532 | 267 |  |  | 0.608 |
| No |  |  | — | — |  |
| Yes |  |  | 0.89 | 0.55, 1.41 |  |
| **Age at onset, years** | 532 | 267 | 1.00 | 0.98, 1.02 | 0.979 |
| **Migraine disease duration, 1-year increase** | 532 | 267 | 1.00 | 0.99, 1.01 | 0.845 |
| **First degree relative with migraine** | 532 | 267 |  |  | 0.806 |
| No |  |  | — | — |  |
| Yes |  |  | 1.05 | 0.72, 1.52 |  |
| **Migraine with aura** | 532 | 267 |  |  | 0.323 |
| No |  |  | — | — |  |
| Yes |  |  | 1.21 | 0.83, 1.76 |  |
| **Chronic migraine** | 532 | 267 |  |  | 0.114 |
| EM |  |  | — | — |  |
| CM |  |  | 0.75 | 0.52, 1.07 |  |
| **Daily headache (28-day baseline)** | 532 | 267 |  |  | 0.003 |
| No |  |  | — | — |  |
| Yes |  |  | 0.48 | 0.30, 0.78 |  |
| **Medication-overuse** | 532 | 267 |  |  | 0.194 |
| No |  |  | — | — |  |
| Yes |  |  | 1.25 | 0.89, 1.77 |  |
| **Monthly headache days (baseline), 1-day increase** | 532 | 267 | 0.95 | 0.92, 0.97 | <0.001 |
| **Monthly migraine days (baseline), 1-day increase** | 532 | 267 | 0.99 | 0.96, 1.02 | 0.657 |
| **Monthly days with use of acute medications (baseline), 1-day increase** | 532 | 267 | 0.98 | 0.95, 1.02 | 0.317 |
| **Ongoing use of preventive medications** | 532 | 267 |  |  | 0.792 |
| No |  |  | — | — |  |
| Yes |  |  | 1.05 | 0.74, 1.47 |  |
| **≥2 or more triptans discontinued due to lack of efficacy (triptan resistance)** | 509 | 252 |  |  | 0.586 |
| No |  |  | — | — |  |
| Yes |  |  | 1.12 | 0.74, 1.71 |  |
| **No. of preventive medications discontinued due to lack of efficacy** | 532 | 267 |  |  | 0.030 |
| <3 |  |  | — | — |  |
| ≥3 |  |  | 0.67 | 0.47, 0.96 |  |
| **No. of preventive drug class failures** | 532 | 267 |  |  | 0.030 |
| <3 |  |  | — | — |  |
| ≥3 |  |  | 0.68 | 0.49, 0.96 |  |
| **MIDAS score (baseline), 1-point increase** | 530 | 266 | 1.00 | 1.00, 1.00 | 0.804 |
| **HIT6 score (baseline), 1-point increase** | 532 | 267 | 1.10 | 1.06, 1.15 | <0.001 |
| **WHODAS score (baseline), 1-point increase** | 526 | 265 | 1.00 | 0.98, 1.02 | 0.909 |
| **Asthma** | 532 | 267 |  |  | 0.291 |
| No |  |  | — | — |  |
| Yes |  |  | 0.73 | 0.40, 1.30 |  |
| **Autoimmunologic conditions** | 532 | 267 |  |  | 0.870 |
| No |  |  | — | — |  |
| Yes |  |  | 0.96 | 0.57, 1.60 |  |
| **Constipation** | 532 | 267 | 1.41 | 0.91, 2.20 | 0.128 |
| **Chronic neck pain (≥3 months)** | 532 | 267 |  |  | 0.932 |
| No |  |  | — | — |  |
| Yes |  |  | 1.02 | 0.63, 1.65 |  |
| **Chronic back pain (≥3 months)** | 532 | 267 |  |  | 0.905 |
| No |  |  | — | — |  |
| Yes |  |  | 1.04 | 0.58, 1.85 |  |
| **Hypertension** | 532 | 267 |  |  | 0.198 |
| No |  |  | — | — |  |
| Yes |  |  | 0.69 | 0.39, 1.21 |  |
| **HADS-D ≥8 (possible or probable risk of depression)** | 530 | 266 |  |  | 0.798 |
| Normal |  |  | — | — |  |
| Possible or probable depression |  |  | 1.05 | 0.71, 1.57 |  |
| **HADS-A ≥8 (possible or probable risk of anxiety)** | 530 | 266 |  |  | 0.366 |
| Normal |  |  | — | — |  |
| Possible or probable anxiety |  |  | 1.19 | 0.82, 1.74 |  |
| **Unilateral headache** | 532 | 267 |  |  | 0.043 |
| Bilateral |  |  | — | — |  |
| Unilateral |  |  | 1.68 | 1.02, 2.80 |  |
| **Side-locked headache** | 532 | 267 |  |  | 0.779 |
| Not side-locked |  |  | — | — |  |
| Side-locked |  |  | 0.93 | 0.58, 1.51 |  |
| **Headache intensity (4-point scale), 1-point increase** | 532 | 267 |  |  | 0.677 |
| Mild |  |  | — | — |  |
| Moderate |  |  | 0.46 | 0.02, 4.89 |  |
| Severe |  |  | 0.52 | 0.02, 5.50 |  |
| **Pain quality: Pulsating** | 532 | 267 |  |  | 0.403 |
| No |  |  | — | — |  |
| Yes |  |  | 0.85 | 0.57, 1.25 |  |
| **Pain aggravation upon physical exercise** | 532 | 267 |  |  | 0.622 |
| No |  |  | — | — |  |
| Yes |  |  | 1.17 | 0.63, 2.17 |  |
| **Photophobia** | 532 | 267 |  |  | 0.298 |
| No |  |  | — | — |  |
| Yes |  |  | 0.64 | 0.26, 1.47 |  |
| **Phonophobia** | 532 | 267 |  |  | 0.555 |
| No |  |  | — | — |  |
| Yes |  |  | 0.83 | 0.45, 1.53 |  |
| **Nausea** | 532 | 267 |  |  | 0.079 |
| No |  |  | — | — |  |
| Yes |  |  | 1.78 | 0.95, 3.45 |  |
| **Vomiting** | 532 | 267 |  |  | 0.652 |
| No |  |  | — | — |  |
| Yes |  |  | 0.92 | 0.66, 1.30 |  |
| **Unilateral autonomic symptoms** | 528 | 266 |  |  | 0.682 |
| None |  |  | — | — |  |
| Present |  |  | 0.92 | 0.60, 1.39 |  |
| **ASC-12 score (baseline), 1-point increase** | 528 | 265 | 1.00 | 0.97, 1.04 | 0.868 |
| *^1^* OR = Odds Ratio, CI = Confidence Interval | | | | | |

Supplementary Material 3. Multivariate logistic regression models

3.1 Primary outcome: ≥50% reduction in MMDs

| **Characteristic** | **N** | **Event N** | **OR***^1^* | **95% CI***^1^* | **p-value** | **q-value***^2^* |
| --- | --- | --- | --- | --- | --- | --- |
| **Age (10-year increase)** | 549 | 285 | 1.22 | 1.06, 1.41 | 0.006 | 0.017 |
| **Obesity (≥30 kg/m2)** | 549 | 285 |  |  | 0.060 | 0.085 |
| No |  |  | — | — |  |  |
| Yes |  |  | 0.63 | 0.39, 1.02 |  |  |
| **≥3 preventive medications discontinued due to lack of efficacy** | 549 | 285 |  |  | <0.001 | 0.005 |
| <3 |  |  | — | — |  |  |
| ≥3 |  |  | 0.54 | 0.37, 0.77 |  |  |
| **HIT-6 score (baseline)** | 549 | 285 | 1.04 | 1.00, 1.09 | 0.071 | 0.085 |
| **ASC-12 score (baseline)** | 549 | 285 | 1.03 | 0.99, 1.08 | 0.125 | 0.125 |
| **Chronic migraine** | 549 | 285 |  |  | 0.015 | 0.030 |
| EM |  |  | — | — |  |  |
| CM |  |  | 0.63 | 0.43, 0.91 |  |  |
| *^1^* OR = Odds Ratio, CI = Confidence Interval | | | | | | |
| *^2^* False discovery rate correction for multiple testing | | | | | | |

3.2 Secondary outcomes: ≥50% reduction in MHDs

| **Characteristic** | **N** | **Event N** | **OR***^1^* | **95% CI***^1^* | **p-value** | **q-value***^2^* |
| --- | --- | --- | --- | --- | --- | --- |
| **Age (10-year increase)** | 552 | 229 | 1.20 | 1.04, 1.40 | 0.015 | 0.041 |
| **Days with acute medication use (1-day increase)** | 552 | 229 | 0.97 | 0.94, 1.01 | 0.105 | 0.140 |
| **≥3 preventive medications discontinued due to lack of efficacy** | 552 | 229 |  |  | 0.023 | 0.046 |
| <3 |  |  | — | — |  |  |
| ≥3 |  |  | 0.64 | 0.44, 0.94 |  |  |
| **MIDAS score (1-point increase)** | 552 | 229 | 1.00 | 1.00, 1.00 | 0.411 | 0.469 |
| **HADS-D ≥8 (possible or probable depression)** | 552 | 229 |  |  | 0.654 | 0.654 |
| Normal |  |  | — | — |  |  |
| Possible or probable depression |  |  | 0.90 | 0.57, 1.42 |  |  |
| **Unilateral headache** | 552 | 229 |  |  | 0.003 | 0.012 |
| Bilateral |  |  | — | — |  |  |
| Unilateral |  |  | 2.31 | 1.34, 4.09 |  |  |
| **Nausea (presence or absence)** | 552 | 229 |  |  | 0.102 | 0.140 |
| No |  |  | — | — |  |  |
| Yes |  |  | 1.78 | 0.91, 3.65 |  |  |
| **Chronic migraine** | 552 | 229 |  |  | <0.001 | 0.005 |
| EM |  |  | — | — |  |  |
| CM |  |  | 0.51 | 0.35, 0.75 |  |  |
| *^1^* OR = Odds Ratio, CI = Confidence Interval | | | | | | |
| *^2^* False discovery rate correction for multiple testing | | | | | | |

3.3 Secondary outcomes: ≥50% reduction in MMDs or moderate-severe MHDs

| **Characteristic** | **N** | **Event N** | **OR***^1^* | **95% CI***^1^* | **p-value** | **q-value***^2^* |
| --- | --- | --- | --- | --- | --- | --- |
| **Age (10-year increase)** | 570 | 359 | 1.34 | 1.15, 1.57 | <0.001 | <0.001 |
| **Obesity (≥30 kg/m2)** | 570 | 359 |  |  | 0.224 | 0.224 |
| No |  |  | — | — |  |  |
| Yes |  |  | 0.75 | 0.47, 1.20 |  |  |
| **≥3 preventive medications discontinued due to lack of efficacy** | 570 | 359 |  |  | 0.012 | 0.020 |
| <3 |  |  | — | — |  |  |
| ≥3 |  |  | 0.62 | 0.43, 0.90 |  |  |
| **Hypertension** | 570 | 359 |  |  | 0.028 | 0.035 |
| No |  |  | — | — |  |  |
| Yes |  |  | 0.52 | 0.29, 0.93 |  |  |
| **Chronic migraine** | 570 | 359 |  |  | 0.006 | 0.014 |
| EM |  |  | — | — |  |  |
| CM |  |  | 0.58 | 0.39, 0.85 |  |  |
| *^1^* OR = Odds Ratio, CI = Confidence Interval | | | | | | |
| *^2^* False discovery rate correction for multiple testing | | | | | | |

3.4 Exploratory outcomes: ≥50% reduction in moderate-severe MHDs

| **Characteristic** | **N** | **Event N** | **OR***^1^* | **95% CI***^1^* | **p-value** | **q-value***^2^* |
| --- | --- | --- | --- | --- | --- | --- |
| **Age (10-year increase)** | 552 | 281 | 1.23 | 1.07, 1.43 | 0.004 | 0.013 |
| **≥3 preventive medications discontinued due to lack of efficacy** | 552 | 281 |  |  | 0.024 | 0.036 |
| <3 |  |  | — | — |  |  |
| ≥3 |  |  | 0.65 | 0.45, 0.94 |  |  |
| **MIDAS score (1-point increase)** | 552 | 281 | 1.00 | 1.0, 1.00 | 0.100 | 0.120 |
| **Unilateral headache** | 552 | 281 |  |  | 0.009 | 0.018 |
| Bilateral |  |  | — | — |  |  |
| Unilateral |  |  | 1.97 | 1.19, 3.31 |  |  |
| **Phonophobia (presence or absence)** | 552 | 281 |  |  | 0.186 | 0.186 |
| No |  |  | — | — |  |  |
| Yes |  |  | 0.65 | 0.34, 1.22 |  |  |
| **Chronic migraine** | 552 | 281 |  |  | <0.001 | 0.002 |
| EM |  |  | — | — |  |  |
| CM |  |  | 0.49 | 0.34, 0.72 |  |  |
| *^1^* OR = Odds Ratio, CI = Confidence Interval | | | | | | |
| *^2^* False discovery rate correction for multiple testing | | | | | | |

3.5 Exploratory outcomes: Reduction in MIDAS

| **Characteristic** | **N** | **Event N** | **OR***^1^* | **95% CI***^1^* | **p-value** | **q-value***^2^* |
| --- | --- | --- | --- | --- | --- | --- |
| **≥3 preventive medications discontinued due to lack of efficacy** | 484 | 350 |  |  | 0.152 | 0.297 |
| <3 |  |  | — | — |  |  |
| ≥3 |  |  | 0.73 | 0.48, 1.12 |  |  |
| **WHODAS 2.0 score (1-point increase)** | 484 | 350 | 0.98 | 0.95, 1.01 | 0.228 | 0.297 |
| **HADS-D ≥8 (possible or probable depression)** | 484 | 350 |  |  | 0.297 | 0.297 |
| Normal |  |  | — | — |  |  |
| Possible or probable depression |  |  | 0.76 | 0.45, 1.28 |  |  |
| **Daily headache (28-day baseline diary)** | 484 | 350 |  |  | <0.001 | <0.001 |
| No |  |  | — | — |  |  |
| Yes |  |  | 0.38 | 0.23, 0.63 |  |  |
| *^1^* OR = Odds Ratio, CI = Confidence Interval | | | | | | |
| *^2^* False discovery rate correction for multiple testing | | | | | | |

3.6 Exploratory outcomes: ≥5-point reduction in HIT-6

| **Characteristic** | **N** | **Event N** | **OR***^1^* | **95% CI***^1^* | **p-value** | **q-value***^2^* |
| --- | --- | --- | --- | --- | --- | --- |
| **Sex** | 532 | 267 |  |  | 0.384 | 0.384 |
| Male |  |  | — | — |  |  |
| Female |  |  | 1.32 | 0.71, 2.52 |  |  |
| **≥3 preventive medications discontinued due to lack of efficacy** | 532 | 267 |  |  | 0.061 | 0.123 |
| <3 |  |  | — | — |  |  |
| ≥3 |  |  | 0.70 | 0.48, 1.02 |  |  |
| **HIT-6 score (1-point increase)** | 532 | 267 | 1.11 | 1.06, 1.17 | <0.001 | <0.001 |
| **Unilateral headache** | 532 | 267 |  |  | 0.219 | 0.263 |
| Bilateral |  |  | — | — |  |  |
| Unilateral |  |  | 1.40 | 0.82, 2.40 |  |  |
| **Nausea (presence or absence)** | 532 | 267 |  |  | 0.114 | 0.170 |
| No |  |  | — | — |  |  |
| Yes |  |  | 1.74 | 0.88, 3.53 |  |  |
| **Daily headache (28-day baseline diary)** | 532 | 267 |  |  | 0.003 | 0.009 |
| No |  |  | — | — |  |  |
| Yes |  |  | 0.46 | 0.27, 0.76 |  |  |
| *^1^* OR = Odds Ratio, CI = Confidence Interval | | | | | | |
| *^2^* False discovery rate correction for multiple testing | | | | | | |

Supplementary Material 4. Additional logistic regression analyses

4.1 Primary outcome: ≥50% reduction in MMDs (disease duration)

This multivariable logistic regression analysis was conducted using disease duration instead of age.

| **Characteristic** | **N** | **Event N** | **OR***^1^* | **95% CI***^1^* | **p-value** | **q-value***^2^* |
| --- | --- | --- | --- | --- | --- | --- |
| **Migraine disease duration, 1-year increase** | 549 | 285 | 1.02 | 1.01, 1.04 | <0.001 | 0.002 |
| **Obesity (≥30 kg/m2)** | 549 | 285 |  |  | 0.051 | 0.077 |
| Not obese |  |  | — | — |  |  |
| Obese |  |  | 0.62 | 0.38, 1.00 |  |  |
| **No. of preventive medications discontinued** | 549 | 285 |  |  | <0.001 | 0.002 |
| <3 |  |  | — | — |  |  |
| ≥3 |  |  | 0.52 | 0.36, 0.75 |  |  |
| **HIT-6 score (baseline)** | 549 | 285 | 1.04 | 1.00, 1.09 | 0.083 | 0.099 |
| **ASC-12 score (baseline)** | 549 | 285 | 1.03 | 0.99, 1.08 | 0.139 | 0.139 |
| **Chronic migraine** | 549 | 285 |  |  | 0.008 | 0.015 |
| EM |  |  | — | — |  |  |
| CM |  |  | 0.60 | 0.41, 0.87 |  |  |
| *^1^* OR = Odds Ratio, CI = Confidence Interval | | | | | | |
| *^2^* False discovery rate correction for multiple testing | | | | | | |

4.2 Primary outcome: ≥50% reduction in MMDs (preventive medication class failures)

This multivariable logistic regression analysis was conducted using preventive medication class failures instead of medications failed due to lack of efficacy.

| **Characteristic** | **N** | **Event N** | **OR*^1^*** | **95% CI*^1^*** | **p-value** | **q-value*^2^*** |
| --- | --- | --- | --- | --- | --- | --- |
| **Age (10-year increase)** | 549 | 285 | 1.25 | 1.08, 1.45 | 0.002 | 0.012 |
| **Obesity (≥30 kg/m2)** | 549 | 285 |  |  | 0.043 | 0.064 |
| Not overweight |  |  | — | — |  |  |
| Overweight |  |  | 0.61 | 0.37, 0.98 |  |  |
| **No. of preventive drug classes discontinued** | 549 | 285 |  |  | 0.004 | 0.012 |
| <3 |  |  | — | — |  |  |
| ≥3 |  |  | 0.60 | 0.42, 0.85 |  |  |
| **HIT-6 score (baseline)** | 549 | 285 | 1.04 | 1.00, 1.09 | 0.063 | 0.076 |
| **ASC-12 score (baseline)** | 549 | 285 | 1.04 | 0.99, 1.08 | 0.102 | 0.102 |
| **Chronic migraine** | 549 | 285 |  |  | 0.023 | 0.047 |
| EM |  |  | — | — |  |  |
| CM |  |  | 0.65 | 0.45, 0.94 |  |  |
| *^1^* OR = Odds Ratio, CI = Confidence Interval | | | | | | |
| *^2^* False discovery rate correction for multiple testing | | | | | | |

4.3 Primary outcome: ≥50% reduction in MMDs (overweight)

This multivariable logistic regression analysis was conducted using overweight (≥25 kg/m^2^) instead of obesity (≥30 kg/m^2^)

| **Characteristic** | **N** | **Event N** | **OR*^1^*** | **95% CI*^1^*** | **p-value** | **q-value*^2^*** |
| --- | --- | --- | --- | --- | --- | --- |
| **Age (10-year increase)** | 549 | 285 | 1.23 | 1.07, 1.43 | 0.005 | 0.014 |
| **Overweight (≥25 kg/m2)** | 549 | 285 |  |  | 0.125 | 0.150 |
| Not overweight |  |  | — | — |  |  |
| Overweight |  |  | 0.76 | 0.53, 1.08 |  |  |
| **No. of preventive medications discontinued** | 549 | 285 |  |  | 0.001 | 0.006 |
| <3 |  |  | — | — |  |  |
| ≥3 |  |  | 0.54 | 0.37, 0.78 |  |  |
| **HIT-6 score (baseline)** | 549 | 285 | 1.04 | 1.00, 1.09 | 0.068 | 0.102 |
| **ASC-12 score (baseline)** | 549 | 285 | 1.03 | 0.99, 1.07 | 0.167 | 0.167 |
| **Chronic migraine** | 549 | 285 |  |  | 0.012 | 0.025 |
| EM |  |  | — | — |  |  |
| CM |  |  | 0.62 | 0.43, 0.90 |  |  |
| *^1^* OR = Odds Ratio, CI = Confidence Interval | | | | | | |
| *^2^* False discovery rate correction for multiple testing | | | | | | |

4.4 Primary outcome: ≥50% reduction in MMDs (inclusion of drop-outs due to adverse events)

The univariate and multivariable regression analyses presented below were conducted with the additional inclusion of participants who discontinued treatment with erenumab due to adverse events and had sufficient baseline diary data to ascertain ≥4 monthly migraine days at baseline (n = 17).

Univariate:

| **Characteristic** | **N** | **Event N** | **OR***^1^* | **95% CI***^1^* | **p-value** |
| --- | --- | --- | --- | --- | --- |
| **Age, 10-year increase** | 587 | 298 | 1.22 | 1.07, 1.40 | 0.003 |
| **Sex** | 587 | 298 |  |  | 0.201 |
| Male |  |  | — | — |  |
| Female |  |  | 0.69 | 0.39, 1.21 |  |
| **Overweight (≥25 kg/m2)** | 587 | 298 |  |  | 0.097 |
| Not overweight |  |  | — | — |  |
| Overweight |  |  | 0.76 | 0.55, 1.05 |  |
| **Obese (≥30 kg/m2)** | 587 | 298 |  |  | 0.032 |
| Not obese |  |  | — | — |  |
| Obese |  |  | 0.62 | 0.40, 0.96 |  |
| **Age at onset, years** | 587 | 298 | 0.99 | 0.98, 1.01 | 0.357 |
| **Migraine disease duration, 1-year increase** | 587 | 298 | 1.02 | 1.01, 1.03 | <0.001 |
| **First degree relative with migraine** | 587 | 298 |  |  | 0.569 |
| No |  |  | — | — |  |
| Yes |  |  | 0.90 | 0.63, 1.29 |  |
| **Migraine with aura** | 587 | 298 |  |  | 0.954 |
| No |  |  | — | — |  |
| Yes |  |  | 0.99 | 0.69, 1.41 |  |
| **Chronic migraine** | 587 | 298 |  |  | 0.016 |
| EM |  |  | — | — |  |
| CM |  |  | 0.66 | 0.46, 0.92 |  |
| **Daily headache (28-day baseline)** | 587 | 298 |  |  | <0.001 |
| No |  |  | — | — |  |
| Yes |  |  | 0.42 | 0.26, 0.67 |  |
| **Medication-overuse** | 587 | 298 |  |  | 0.409 |
| No |  |  | — | — |  |
| Yes |  |  | 1.15 | 0.83, 1.59 |  |
| **Monthly headache days (baseline), 1-day increase** | 587 | 298 | 0.97 | 0.94, 0.99 | 0.007 |
| **Monthly migraine days (baseline), 1-day increase** | 587 | 298 | 1.00 | 0.97, 1.03 | 0.994 |
| **Monthly days with use of acute medications (baseline), 1-day increase** | 587 | 298 | 0.99 | 0.96, 1.02 | 0.431 |
| **Ongoing use of preventive medications** | 587 | 298 |  |  | 0.382 |
| No |  |  | — | — |  |
| Yes |  |  | 1.16 | 0.84, 1.60 |  |
| **≥2 or more triptans discontinued due to lack of efficacy (triptan resistance)** | 560 | 282 |  |  | 0.106 |
| No |  |  | — | — |  |
| Yes |  |  | 0.72 | 0.48, 1.07 |  |
| **No. of preventive medications discontinued due to lack of efficacy** | 587 | 298 |  |  | <0.001 |
| <3 |  |  | — | — |  |
| ≥3 |  |  | 0.53 | 0.37, 0.75 |  |
| **No. of preventive drug class failures** | 587 | 298 |  |  | 0.010 |
| <3 |  |  | — | — |  |
| ≥3 |  |  | 0.65 | 0.47, 0.90 |  |
| **Botox discontinued due to lack of efficacy** | 122 | 59 |  |  | 0.551 |
| No |  |  | — | — |  |
| Yes |  |  | 0.80 | 0.39, 1.65 |  |
| **MIDAS score (baseline), 1-point increase** | 569 | 287 | 1.00 | 1.00, 1.00 | 0.729 |
| **HIT6 score (baseline), 1-point increase** | 572 | 289 | 1.03 | 1.0, 1.08 | 0.090 |
| **WHODAS score (baseline), 1-point increase** | 564 | 284 | 1.01 | 0.99, 1.03 | 0.448 |
| **Asthma** | 587 | 298 |  |  | 0.583 |
| No |  |  | — | — |  |
| Yes |  |  | 1.16 | 0.68, 1.99 |  |
| **Autoimmunologic conditions** | 587 | 298 |  |  | 0.332 |
| No |  |  | — | — |  |
| Yes |  |  | 1.27 | 0.78, 2.08 |  |
| **Constipation** | 587 | 298 | 1.10 | 0.73, 1.68 | 0.644 |
| **Chronic neck pain (≥3 months)** | 587 | 298 |  |  | 0.109 |
| No |  |  | — | — |  |
| Yes |  |  | 1.46 | 0.92, 2.35 |  |
| **Chronic back pain (≥3 months)** | 587 | 298 |  |  | 0.471 |
| No |  |  | — | — |  |
| Yes |  |  | 1.23 | 0.71, 2.15 |  |
| **Hypertension** | 587 | 298 |  |  | 0.284 |
| No |  |  | — | — |  |
| Yes |  |  | 0.75 | 0.44, 1.27 |  |
| **HADS-D ≥8 (possible or probable risk of depression)** | 569 | 287 |  |  | 0.419 |
| Not possible depression |  |  | — | — |  |
| Possible or probable depression |  |  | 0.85 | 0.58, 1.26 |  |
| **HADS-A ≥8 (possible or probable risk of anxiety)** | 569 | 287 |  |  | 0.739 |
| Not possible anxiety |  |  | — | — |  |
| Possible or probable anxiety |  |  | 1.06 | 0.74, 1.53 |  |
| **Unilateral headache** | 587 | 298 |  |  | 0.280 |
| Bilateral |  |  | — | — |  |
| Unilateral |  |  | 1.28 | 0.82, 2.03 |  |
| **Side-locked headache** | 587 | 298 |  |  | 0.787 |
| Not side-locked |  |  | — | — |  |
| Side-locked |  |  | 0.94 | 0.59, 1.49 |  |
| **Headache intensity (4-point scale), 1-point increase** | 587 | 298 |  |  | 0.512 |
| Mild |  |  | — | — |  |
| Moderate |  |  | 1.84 | 0.17, 40.0 |  |
| Severe |  |  | 2.19 | 0.21, 47.4 |  |
| **Pain quality: Pulsating** | 587 | 298 |  |  | 0.953 |
| No |  |  | — | — |  |
| Yes |  |  | 0.99 | 0.68, 1.43 |  |
| **Pain aggravation upon physical exercise** | 587 | 298 |  |  | 0.119 |
| No |  |  | — | — |  |
| Yes |  |  | 1.58 | 0.89, 2.86 |  |
| **Photophobia** | 587 | 298 |  |  | 0.936 |
| No |  |  | — | — |  |
| Yes |  |  | 1.03 | 0.47, 2.29 |  |
| **Phonophobia** | 587 | 298 |  |  | 0.975 |
| No |  |  | — | — |  |
| Yes |  |  | 0.99 | 0.56, 1.76 |  |
| **Nausea** | 587 | 298 |  |  | 0.190 |
| No |  |  | — | — |  |
| Yes |  |  | 1.49 | 0.82, 2.75 |  |
| **Vomiting** | 587 | 298 |  |  | 0.324 |
| No |  |  | — | — |  |
| Yes |  |  | 1.18 | 0.85, 1.64 |  |
| **Unilateral autonomic symptoms** | 582 | 294 |  |  | 0.593 |
| None |  |  | — | — |  |
| Present |  |  | 0.90 | 0.60, 1.33 |  |
| **ASC-12 score (baseline), 1-point increase** | 566 | 285 | 1.03 | 1.00, 1.07 | 0.088 |
| *^1^* OR = Odds Ratio, CI = Confidence Interval | | | | | |

Multivariable (primary outcome ≥50% reduction in MMDs, chronic migraine):

| **Characteristic** | **N** | **Event N** | **OR*^1^*** | **95% CI*^1^*** | **p-value** | **q-value*^2^*** |
| --- | --- | --- | --- | --- | --- | --- |
| **Age (10-year increase)** | 566 | 285 | 1.24 | 1.08, 1.43 | 0.003 | 0.009 |
| **Obesity (≥30 kg/m2)** | 566 | 285 |  |  | 0.038 | 0.058 |
| Not obese |  |  | — | — |  |  |
| Obese |  |  | 0.61 | 0.38, 0.97 |  |  |
| **No. of preventive medications discontinued** | 566 | 285 |  |  | <0.001 | 0.004 |
| <3 |  |  | — | — |  |  |
| ≥3 |  |  | 0.53 | 0.37, 0.76 |  |  |
| **HIT-6 score (baseline)** | 566 | 285 | 1.04 | 1.00, 1.09 | 0.059 | 0.071 |
| **ASC-12 score (baseline)** | 566 | 285 | 1.03 | 0.99, 1.08 | 0.132 | 0.132 |
| **Chronic migraine** | 566 | 285 |  |  | 0.015 | 0.030 |
| EM |  |  | — | — |  |  |
| CM |  |  | 0.63 | 0.44, 0.91 |  |  |
| *^1^* OR = Odds Ratio, CI = Confidence Interval | | | | | | |
| *^2^* False discovery rate correction for multiple testing | | | | | | |

Multivariable (primary outcome ≥50% reduction in MMDs, chronic migraine; overweight):

| **Characteristic** | **N** | **Event N** | **OR***^1^* | **95% CI***^1^* | **p-value** | **q-value***^2^* |
| --- | --- | --- | --- | --- | --- | --- |
| **Age (10-year increase)** | 566 | 285 | 1.25 | 1.08, 1.44 | 0.002 | 0.007 |
| **Overweight (≥25 kg/m2)** | 566 | 285 |  |  | 0.083 | 0.100 |
| Not overweight |  |  | — | — |  |  |
| Overweight |  |  | 0.74 | 0.52, 1.04 |  |  |
| **No. of preventive medications discontinued** | 566 | 285 |  |  | <0.001 | 0.005 |
| <3 |  |  | — | — |  |  |
| ≥3 |  |  | 0.54 | 0.37, 0.77 |  |  |
| **HIT-6 score (baseline)** | 566 | 285 | 1.04 | 1.00, 1.09 | 0.055 | 0.082 |
| **ASC-12 score (baseline)** | 566 | 285 | 1.03 | 0.99, 1.07 | 0.179 | 0.179 |
| **Chronic migraine** | 566 | 285 |  |  | 0.012 | 0.025 |
| EM |  |  | — | — |  |  |
| CM |  |  | 0.63 | 0.43, 0.90 |  |  |
| *^1^* OR = Odds Ratio, CI = Confidence Interval | | | | | | |
| *^2^* False discovery rate correction for multiple testing | | | | | | |

Multivariable (primary outcome ≥50% reduction in MMDs, chronic migraine; migraine disease duration):

| **Characteristic** | **N** | **Event N** | **OR***^1^* | **95% CI***^1^* | **p-value** | **q-value***^2^* |
| --- | --- | --- | --- | --- | --- | --- |
| **Migraine disease duration, 1-year increase** | 566 | 285 | 1.02 | 1.01, 1.04 | <0.001 | 0.001 |
| **Obesity (≥30 kg/m2)** | 566 | 285 |  |  | 0.033 | 0.050 |
| Not obese |  |  | — | — |  |  |
| Obese |  |  | 0.60 | 0.37, 0.96 |  |  |
| **No. of preventive medications discontinued** | 566 | 285 |  |  | <0.001 | 0.001 |
| <3 |  |  | — | — |  |  |
| ≥3 |  |  | 0.52 | 0.36, 0.75 |  |  |
| **HIT-6 score (baseline)** | 566 | 285 | 1.04 | 1.00, 1.09 | 0.071 | 0.086 |
| **ASC-12 score (baseline)** | 566 | 285 | 1.03 | 0.99, 1.07 | 0.150 | 0.150 |
| **Chronic migraine** | 566 | 285 |  |  | 0.007 | 0.014 |
| EM |  |  | — | — |  |  |
| CM |  |  | 0.60 | 0.42, 0.87 |  |  |
| *^1^* OR = Odds Ratio, CI = Confidence Interval | | | | | | |
| *^2^* False discovery rate correction for multiple testing | | | | | | |

Multivariable (primary outcome ≥50% reduction in MMDs, chronic migraine; preventive classes):

| **Characteristic** | **N** | **Event N** | **OR***^1^* | **95% CI***^1^* | **p-value** | **q-value***^2^* |
| --- | --- | --- | --- | --- | --- | --- |
| **Age (10-year increase)** | 566 | 285 | 1.02 | 1.01, 1.04 | <0.001 | 0.001 |
| **Obesity (≥30 kg/m2)** | 566 | 285 |  |  | 0.033 | 0.050 |
| Not obese |  |  | — | — |  |  |
| Obese |  |  | 0.60 | 0.37, 0.96 |  |  |
| **No. of preventive drug classes discontinued** | 566 | 285 |  |  | <0.001 | 0.001 |
| <3 |  |  | — | — |  |  |
| ≥3 |  |  | 0.52 | 0.36, 0.75 |  |  |
| **HIT-6 score (baseline)** | 566 | 285 | 1.04 | 1.00, 1.09 | 0.071 | 0.086 |
| **ASC-12 score (baseline)** | 566 | 285 | 1.03 | 0.99, 1.07 | 0.150 | 0.150 |
| **Chronic migraine** | 566 | 285 |  |  | 0.007 | 0.014 |
| EM |  |  | — | — |  |  |
| CM |  |  | 0.60 | 0.42, 0.87 |  |  |
| *^1^* OR = Odds Ratio, CI = Confidence Interval | | | | | | |
| *^2^* False discovery rate correction for multiple testing | | | | | | |

Multivariable (primary outcome ≥50% reduction in MMDs, daily headache):

| **Characteristic** | **N** | **Event N** | **OR***^1^* | **95% CI***^1^* | **p-value** | **q-value***^2^* |
| --- | --- | --- | --- | --- | --- | --- |
| **Age (10-year increase)** | 566 | 285 | 1.21 | 1.05, 1.40 | 0.010 | 0.019 |
| **Obesity (≥30 kg/m2)** | 566 | 285 |  |  | 0.080 | 0.096 |
| Not obese |  |  | — | — |  |  |
| Obese |  |  | 0.65 | 0.40, 1.05 |  |  |
| **No. of preventive medications discontinued** | 566 | 285 |  |  | 0.001 | 0.004 |
| <3 |  |  | — | — |  |  |
| ≥3 |  |  | 0.55 | 0.38, 0.78 |  |  |
| **HIT-6 score (baseline)** | 566 | 285 | 1.04 | 1.00, 1.09 | 0.071 | 0.096 |
| **ASC-12 score (baseline)** | 566 | 285 | 1.03 | 0.99, 1.07 | 0.167 | 0.167 |
| **Daily headache (baseline diary)** | 566 | 285 |  |  | 0.001 | 0.004 |
| No |  |  | — | — |  |  |
| Yes |  |  | 0.44 | 0.26, 0.72 |  |  |
| *^1^* OR = Odds Ratio, CI = Confidence Interval | | | | | | |
| *^2^* False discovery rate correction for multiple testing | | | | | | |

Multivariable (primary outcome ≥50% reduction in MMDs, MHDs):

| **Characteristic** | **N** | **Event N** | **OR***^1^* | **95% CI***^1^* | **p-value** | **q-value***^2^* |
| --- | --- | --- | --- | --- | --- | --- |
| **Age (10-year increase)** | 566 | 285 | 1.22 | 1.06, 1.41 | 0.006 | 0.018 |
| **Obesity (≥30 kg/m2)** | 566 | 285 |  |  | 0.064 | 0.080 |
| Not obese |  |  | — | — |  |  |
| Obese |  |  | 0.64 | 0.39, 1.02 |  |  |
| **No. of preventive medications discontinued** | 566 | 285 |  |  | 0.001 | 0.007 |
| <3 |  |  | — | — |  |  |
| ≥3 |  |  | 0.55 | 0.38, 0.79 |  |  |
| **HIT-6 score (baseline)** | 566 | 285 | 1.04 | 1.00, 1.09 | 0.067 | 0.080 |
| **ASC-12 score (baseline)** | 566 | 285 | 1.03 | 0.99, 1.07 | 0.164 | 0.164 |
| **Monthly headache days (baseline), 1-day increase** | 566 | 285 | 0.97 | 0.95, 1.00 | 0.044 | 0.080 |
| *^1^* OR = Odds Ratio, CI = Confidence Interval | | | | | | |
| *^2^* False discovery rate correction for multiple testing | | | | | | |

ROC curves

Supplementary Fig. 1. ROC curve: Primary outcome: ≥50% reduction in MMDs

**
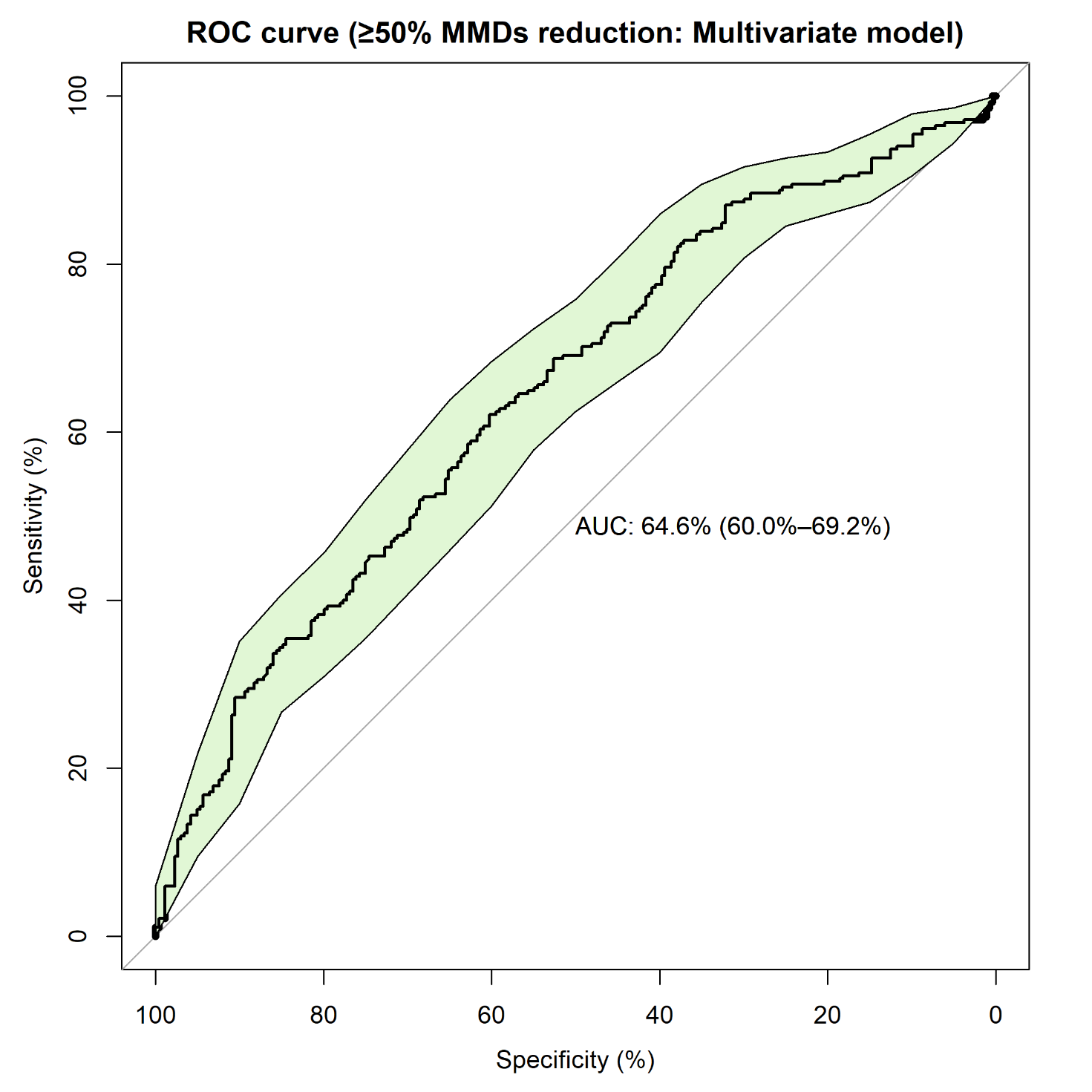
**

**Receiver Operating Characteristic (ROC) curve for the multivariate model predicting ≥50% reduction in Monthly Migraine Days (MMDs).** The x-axis represents specificity (%) and the y-axis represents sensitivity (%). The black line indicates the ROC curve, with the shaded green area representing the 95% confidence interval. The diagonal gray line represents the line of no discrimination (random chance). The area under the curve (AUC) is 64.6% (95% CI: 60.0%–69.2%), suggesting moderate discriminatory ability of the model. The sample size was 549.

Supplementary Fig. 2. ROC curve: Secondary outcomes: ≥50% reduction in MHDs

**
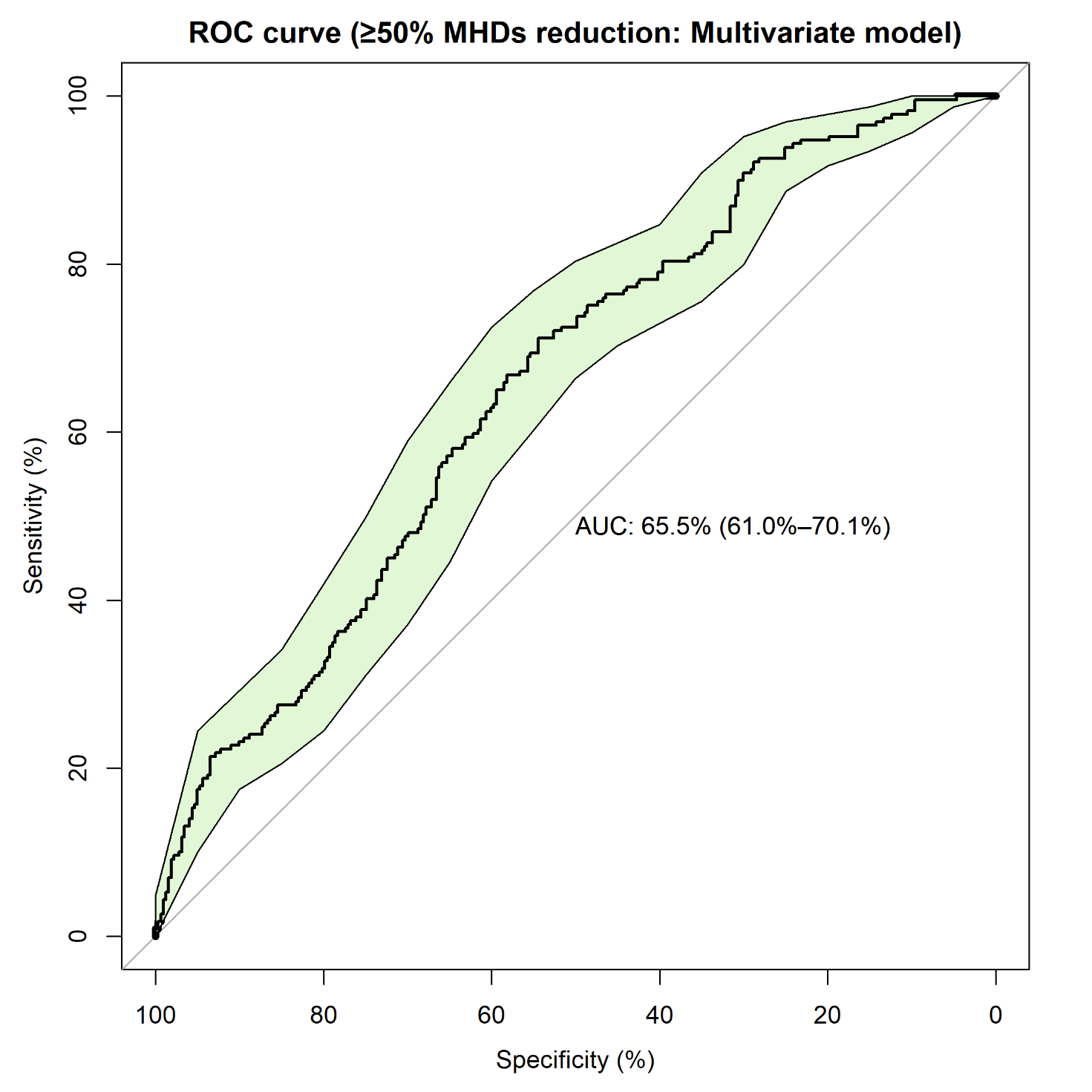
**

**Receiver Operating Characteristic (ROC) curve for the multivariate model predicting ≥50% reduction in Monthly Headache Days (MHDs).** The x-axis represents specificity (%) and the y-axis represents sensitivity (%). The black line indicates the ROC curve, with the shaded green area representing the 95% confidence interval. The diagonal gray line represents the line of no discrimination (random chance). The area under the curve (AUC) is 65.5% (95% CI: 61.0%–70.1%), suggesting moderate discriminatory ability of the model. The sample size was 552.

Supplementary Fig. 3. ROC curve: Secondary outcomes: ≥50% reduction in MMDs or moderate-severe MHDs

**
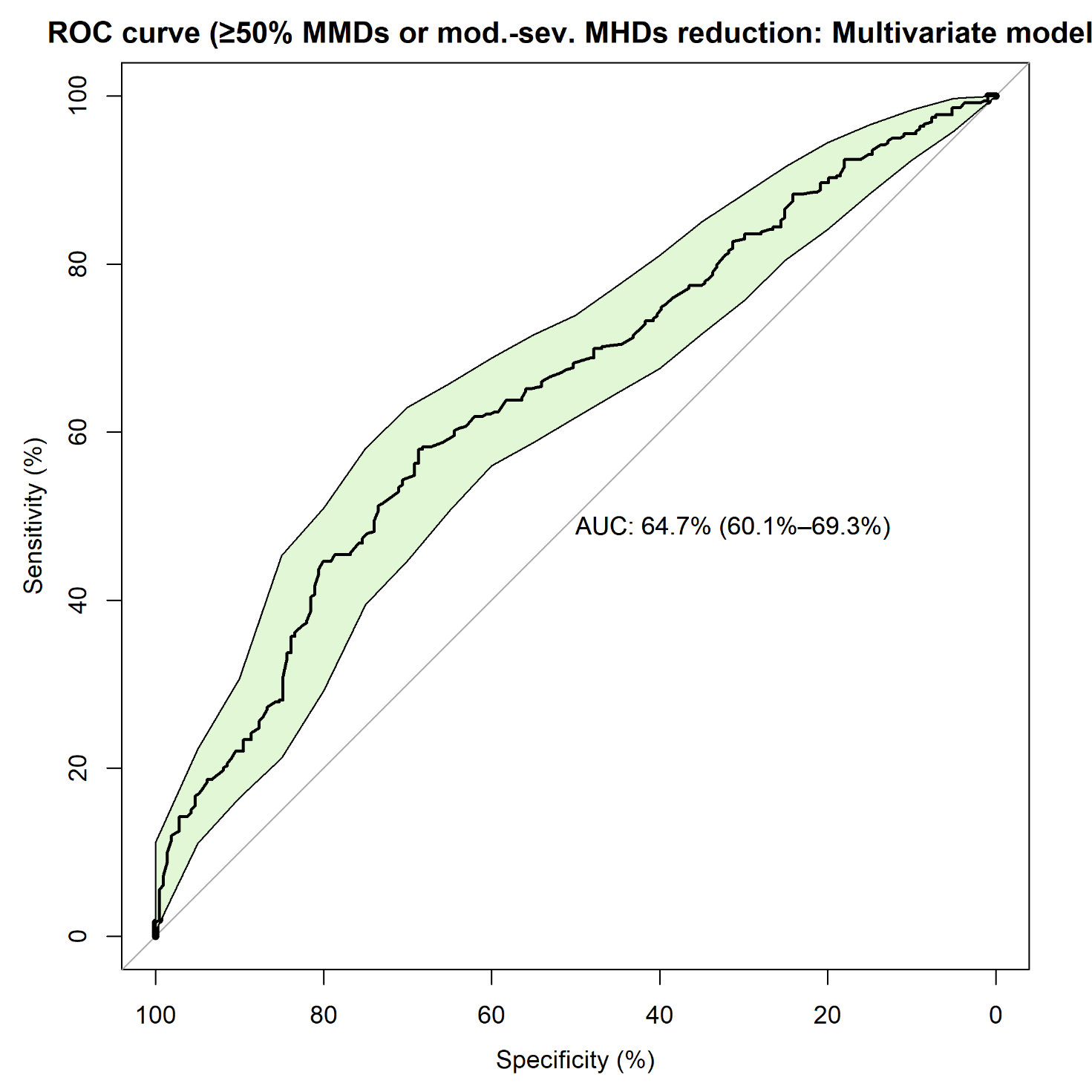
**

**Receiver Operating Characteristic (ROC) curve for the multivariate model predicting ≥50% reduction in Monthly Migraine Days (MMDs) or moderate-to-severe Monthly Headache Days (MHDs).** The x-axis represents specificity (%) and the y-axis represents sensitivity (%). The black line indicates the ROC curve, with the shaded green area representing the 95% confidence interval. The diagonal gray line represents the line of no discrimination (random chance). The area under the curve (AUC) is 64.7% (95% CI: 60.1%–69.3%), suggesting moderate discriminatory ability of the model. The sample size was 570.

Supplementary Fig. 4. ROC curve: Exploratory outcomes: ≥50% reduction in moderate-severe MHDs

**
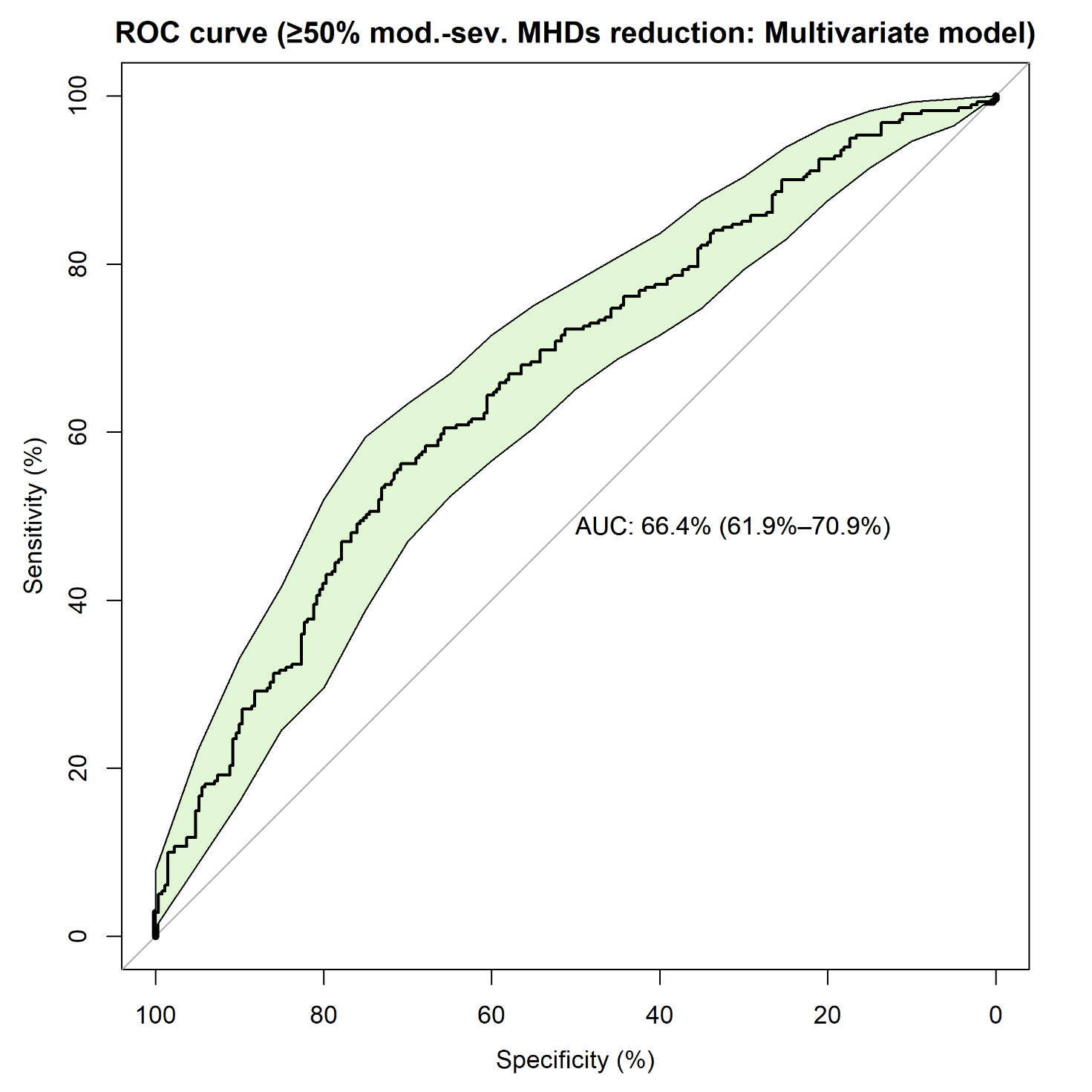
**

**Receiver Operating Characteristic (ROC) curve for the multivariate model predicting ≥50% reduction in moderate-to-severe Monthly Headache Days (MHDs).** The x-axis represents specificity (%) and the y-axis represents sensitivity (%). The black line indicates the ROC curve, with the shaded green area representing the 95% confidence interval. The diagonal gray line represents the line of no discrimination (random chance). The area under the curve (AUC) is 66.4% (95% CI: 61.9%–70.9%), suggesting moderate discriminatory ability of the model. The sample size was 552.

Supplementary Fig. 5. ROC curve: Exploratory outcomes: Reduction in MIDAS

**
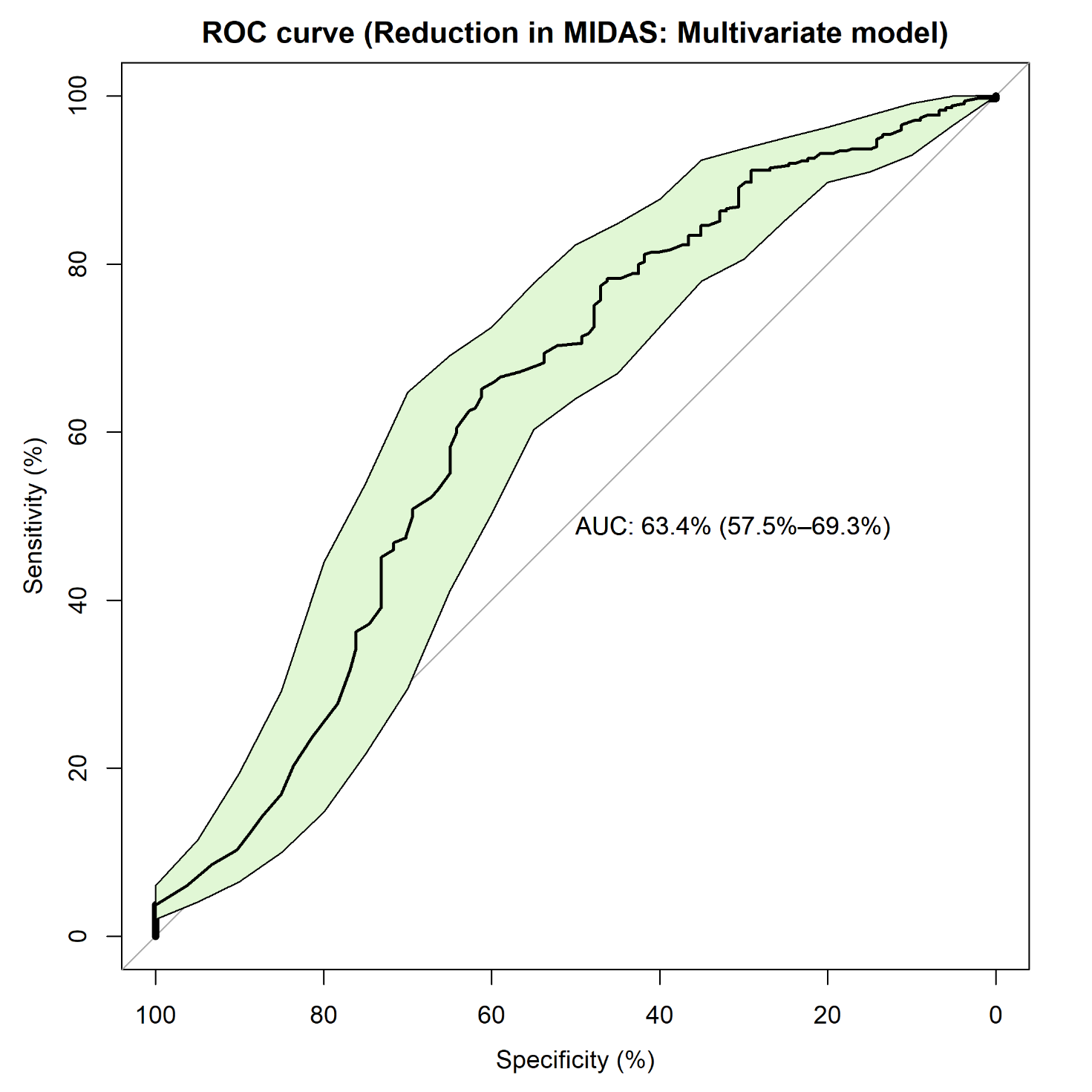
**

**Receiver Operating Characteristic (ROC) curve for the multivariate model predicting ≥5-point reduction in Migraine Disability Assessment Score (MIDAS) scores for participants with baseline scores between 11–20 points or a ≥30% reduction for those with baseline scores ≥21.** The x-axis represents specificity (%) and the y-axis represents sensitivity (%). The black line indicates the ROC curve, with the shaded green area representing the 95% confidence interval. The diagonal gray line represents the line of no discrimination (random chance). The area under the curve (AUC) is 63.4% (95% CI: 57.5%–69.3%), suggesting moderate discriminatory ability of the model. The sample size was 484.

Supplementary Fig. 6. ROC curve: Exploratory outcomes: ≥5-point reduction in HIT-6

**
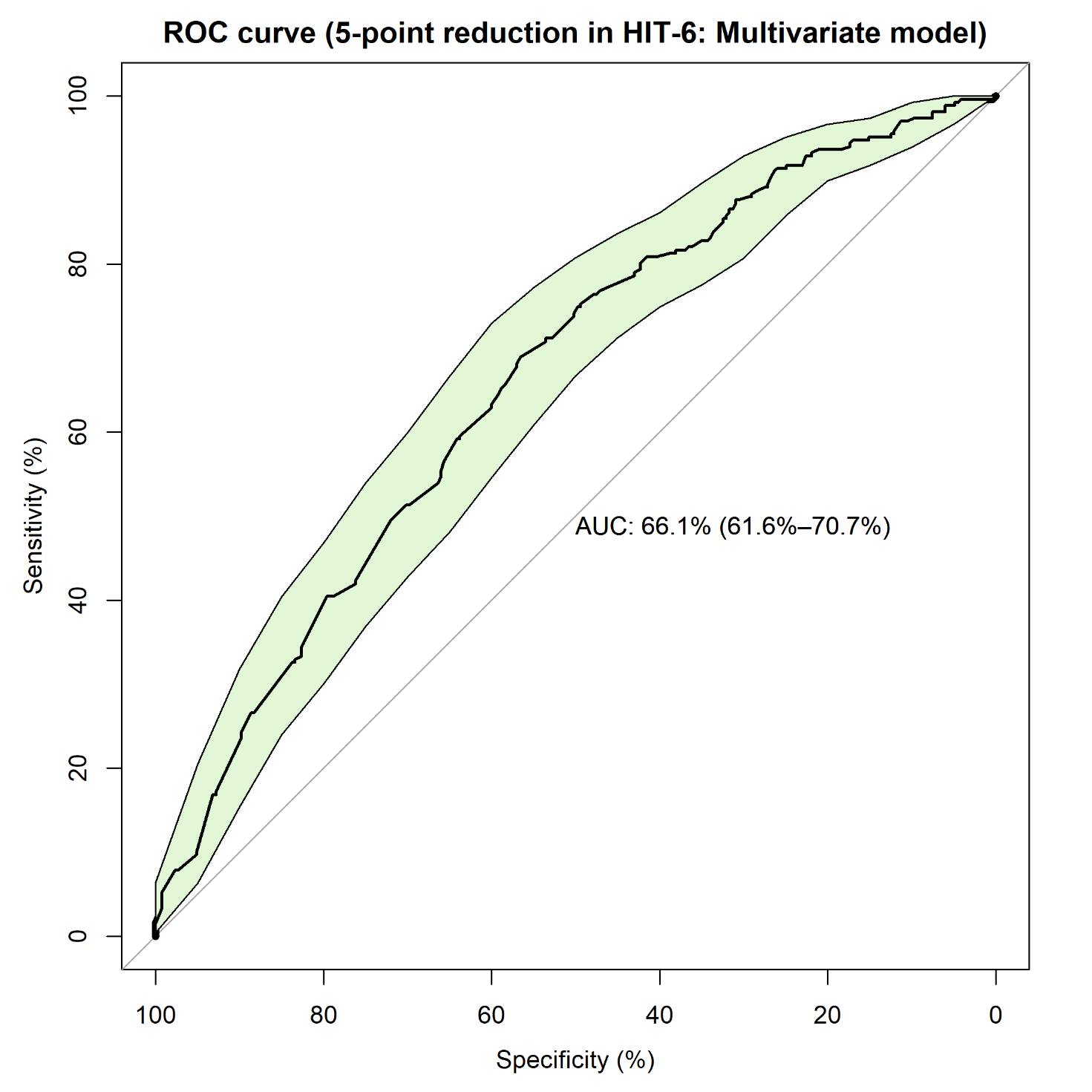
**

**Receiver Operating Characteristic (ROC) curve for the multivariate model predicting ≥5-point reduction in Headache Impact Test-6 (HIT-6) scores.** The x-axis represents specificity (%) and the y-axis represents sensitivity (%). The black line indicates the ROC curve, with the shaded green area representing the 95% confidence interval. The diagonal gray line represents the line of no discrimination (random chance). The area under the curve (AUC) is 66.1% (95% CI: 61.6%–70.7%), suggesting moderate discriminatory ability of the model. The sample size was 532.

Calibration curves

Supplementary Fig. 7. Calibration curve: Primary outcome: ≥50% reduction in MMDs

**
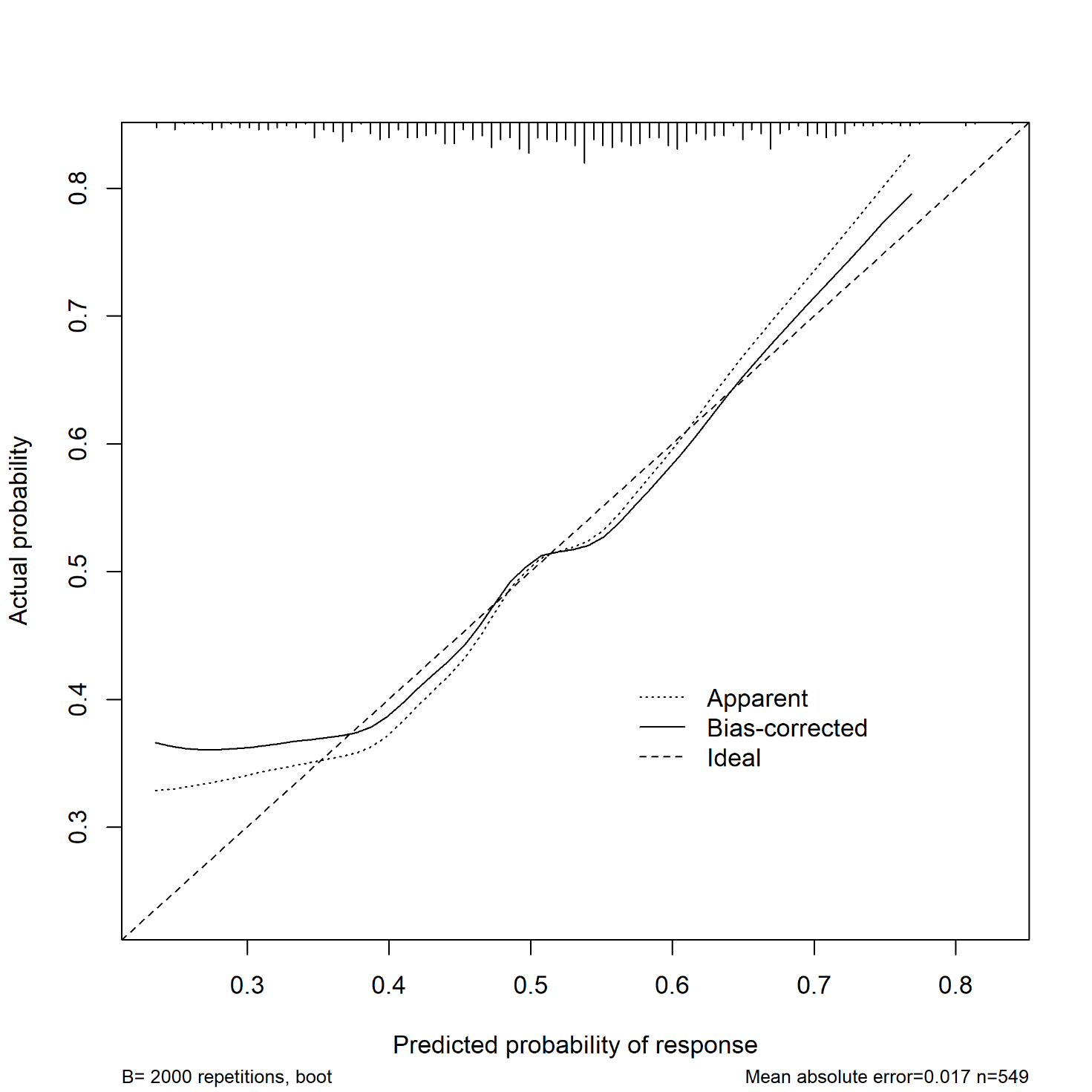
**

**Calibration plot for the prediction of ≥50% reduction in Monthly Migraine Days (MMDs).** The x-axis represents the predicted probability of response, and the y-axis represents the actual observed probability. The dashed line indicates the ideal calibration (perfect agreement between predicted and observed probabilities). The solid line represents the bias-corrected calibration curve, and the dotted line shows the apparent calibration without correction for overfitting. Calibration was assessed using 2000 bootstrap resampling iterations. The mean absolute error is 0.017, based on a sample size of 549.

Supplementary Fig. 8. Calibration curve: Secondary outcomes: ≥50% reduction in MHDs

**
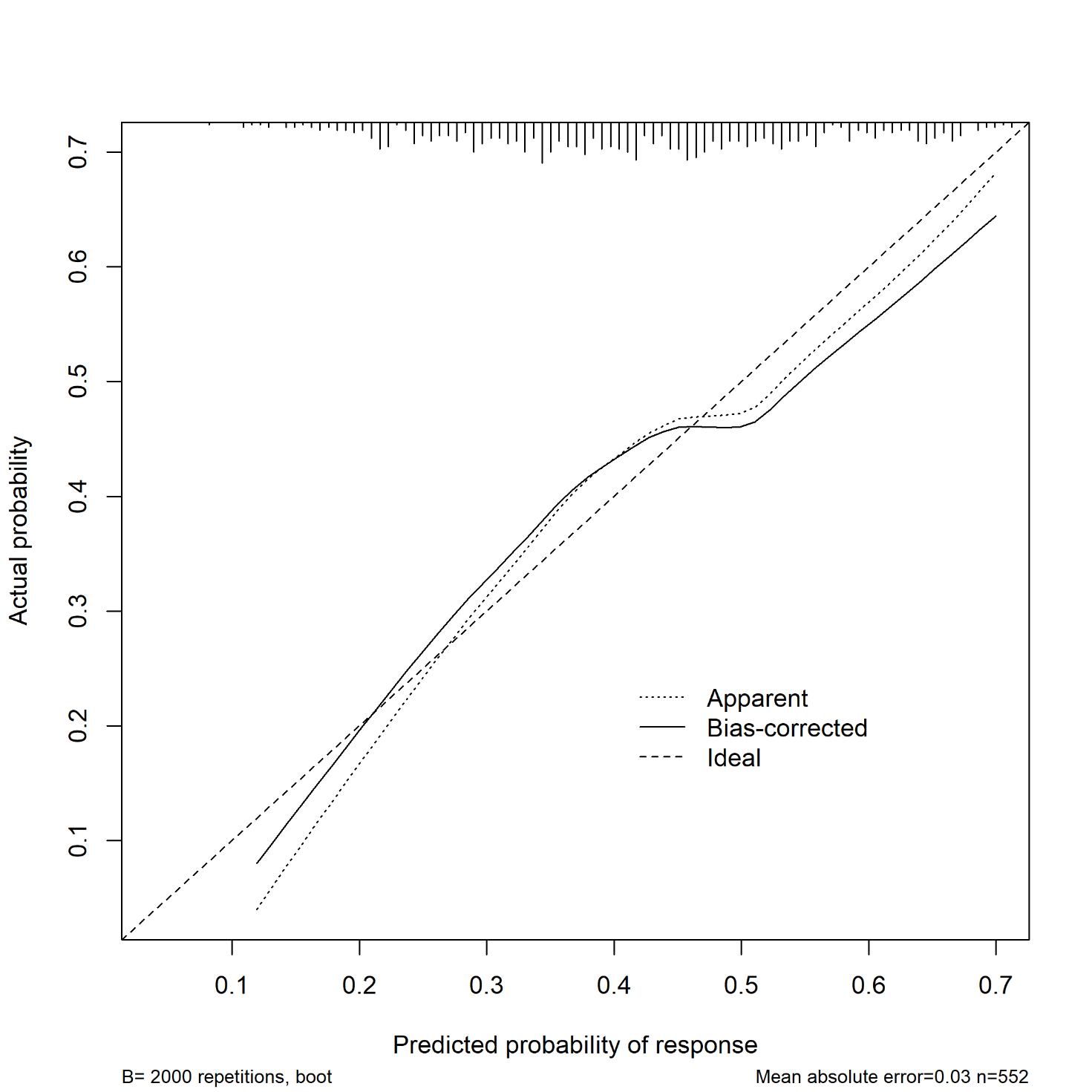
**

**Calibration plot for the prediction of ≥50% reduction in Monthly Headache Days (MHDs).** The x-axis represents the predicted probability of response, and the y-axis represents the actual observed probability. The dashed line indicates the ideal calibration (perfect agreement between predicted and observed probabilities). The solid line represents the bias-corrected calibration curve, and the dotted line shows the apparent calibration without correction for overfitting. Calibration was assessed using 2000 bootstrap resampling iterations. The mean absolute error is 0.030, based on a sample size of 552.

Supplementary Fig. 9. Calibration curve: Secondary outcomes: ≥50% reduction in MMDs or moderate-severe MHDs

**
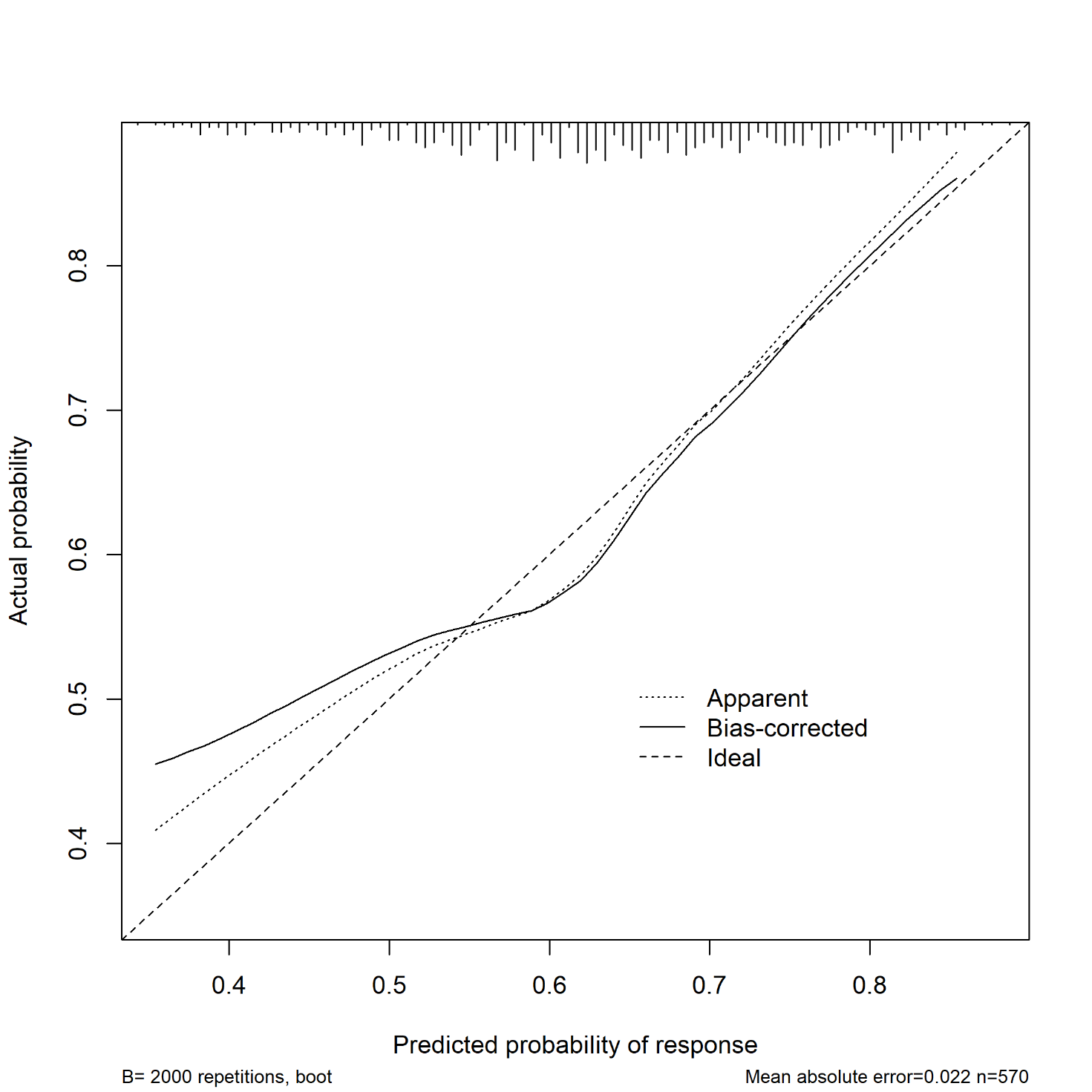
**

**Calibration plot for the prediction of ≥50% reduction in Monthly Migraine Days or moderate-to-severe Monthly Headache Days (MHDs).** The x-axis represents the predicted probability of response, and the y-axis represents the actual observed probability. The dashed line indicates the ideal calibration (perfect agreement between predicted and observed probabilities). The solid line represents the bias-corrected calibration curve, and the dotted line shows the apparent calibration without correction for overfitting. Calibration was assessed using 2000 bootstrap resampling iterations. The mean absolute error is 0.022, based on a sample size of 570.

Supplementary Fig. 10. Calibration curve: Exploratory outcomes: ≥50% reduction in moderate-severe MHDs

**
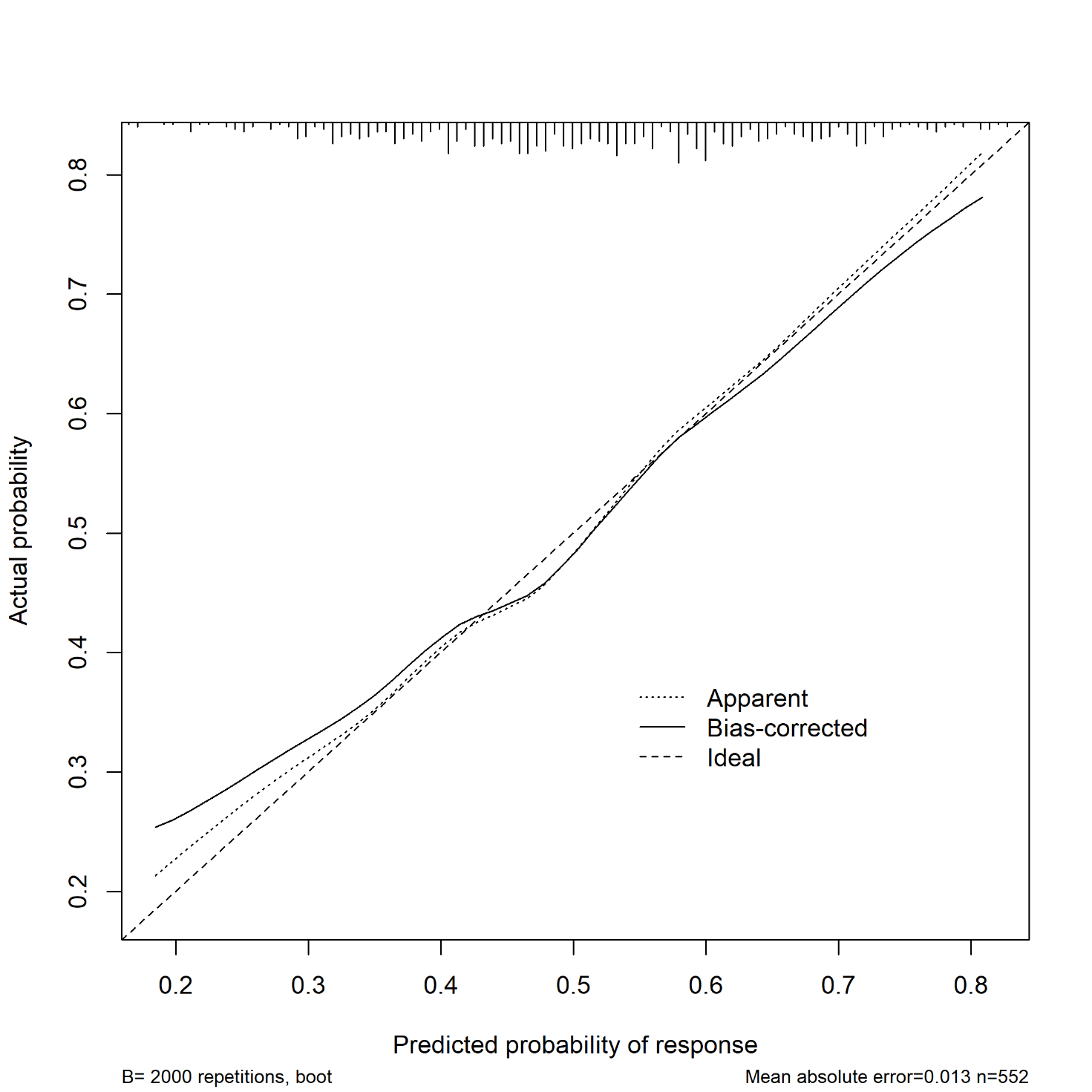
**

**Calibration plot for the prediction of ≥50% reduction in moderate-to-severe Monthly Headache Days (MHDs).** The x-axis represents the predicted probability of response, and the y-axis represents the actual observed probability. The dashed line indicates the ideal calibration (perfect agreement between predicted and observed probabilities). The solid line represents the bias-corrected calibration curve, and the dotted line shows the apparent calibration without correction for overfitting. Calibration was assessed using 2000 bootstrap resampling iterations. The mean absolute error is 0.013, based on a sample size of 552.

Supplementary Fig. 11. Calibration curve: Exploratory outcomes: Reduction in MIDAS

**
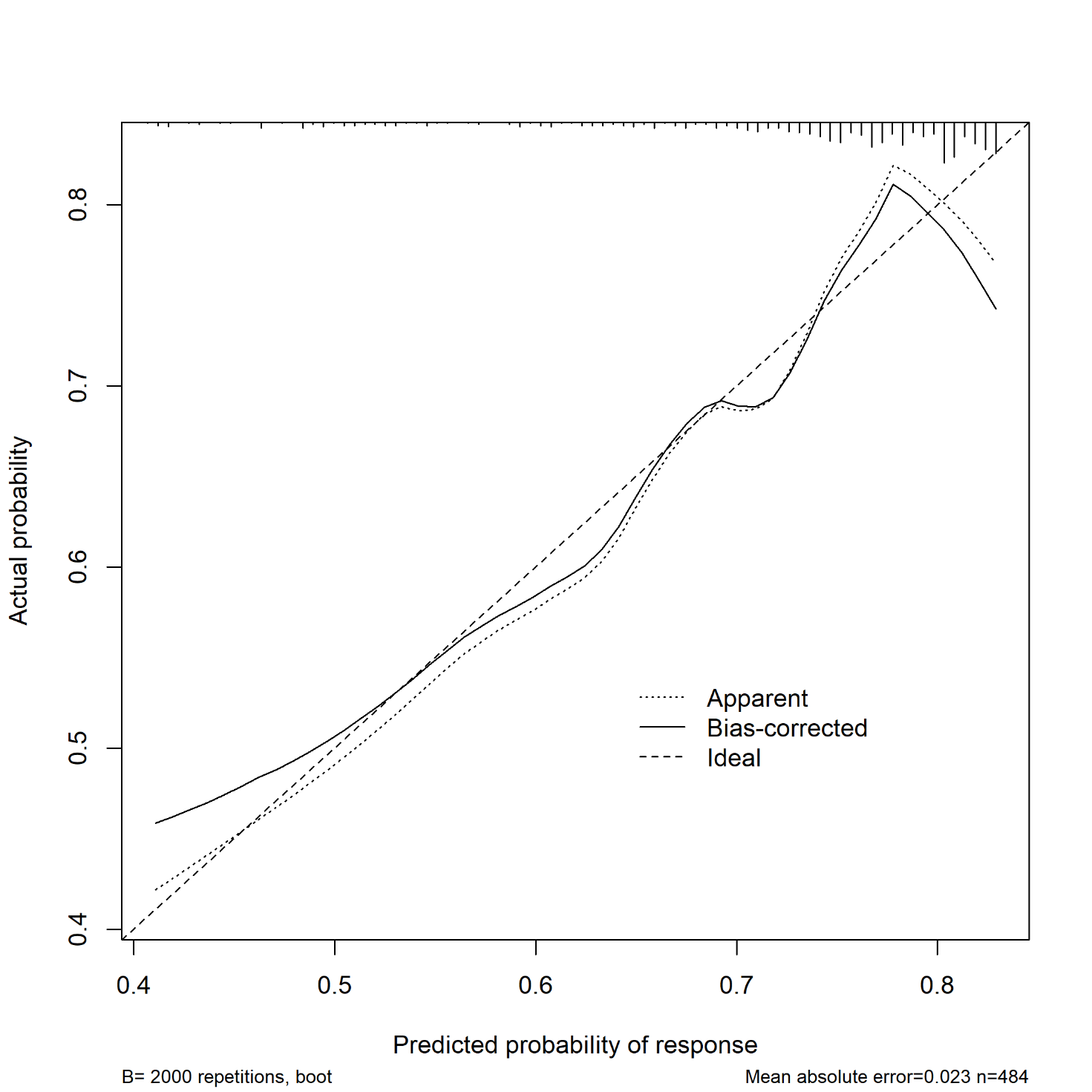
**

**Calibration plot for the prediction of ≥5-point reduction in Migraine Disability Assessment Score (MIDAS) scores for participants with baseline scores between 11–20 points or a ≥30% reduction for those with baseline scores ≥21.** The x-axis represents the predicted probability of response, and the y-axis represents the actual observed probability. The dashed line indicates the ideal calibration (perfect agreement between predicted and observed probabilities). The solid line represents the bias-corrected calibration curve, and the dotted line shows the apparent calibration without correction for overfitting. Calibration was assessed using 2000 bootstrap resampling iterations. The mean absolute error is 0.023, based on a sample size of 484.

Supplementary Fig. 12. Calibration curve: Exploratory outcomes: ≥5-point reduction in HIT-6

**
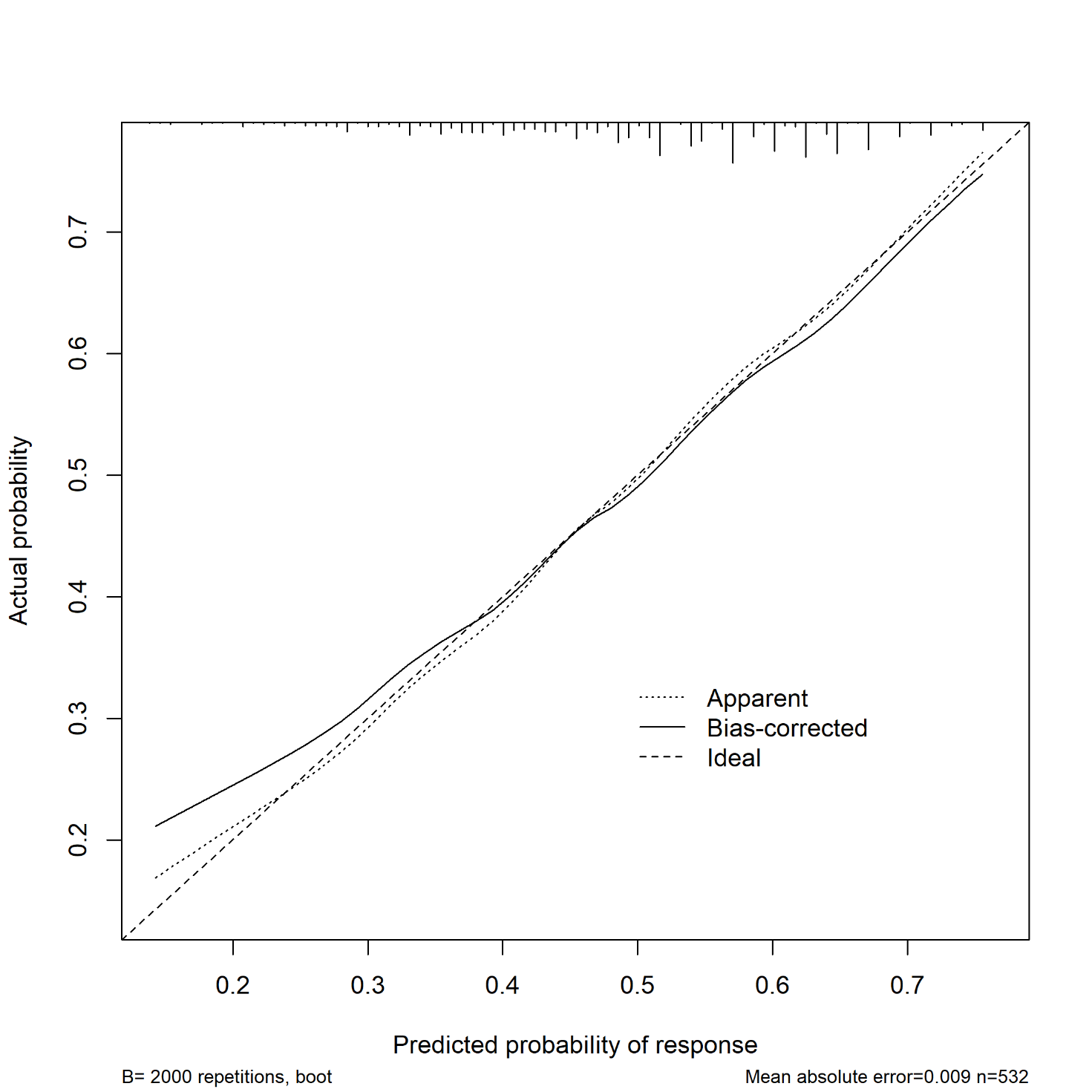
**

**Calibration plot for the prediction of ≥5-point reduction in Headache Impact Test-6 (HIT-6) scores.** The x-axis represents the predicted probability of response, and the y-axis represents the actual observed probability. The dashed line indicates the ideal calibration (perfect agreement between predicted and observed probabilities). The solid line represents the bias-corrected calibration curve, and the dotted line shows the apparent calibration without correction for overfitting. Calibration was assessed using 2000 bootstrap resampling iterations. The mean absolute error is 0.009, based on a sample size of 532.

Supplementary Tables.

Supplementary Table 1. Low- and high-frequency episodic migraine (baseline characteristics)

|  | **CM**  **(n = 373)** | **High-frequency EM**  **(n = 149)** | **Low-frequency EM**  **(n = 48)** | ***P^b^*** |
| --- | --- | --- | --- | --- |
| **Treatment response** |  |  |  |  |
| Response to erenumab^a^, n (%) | 181 (48.5%) | 84 (56.4%) | 33 (68.8%) | 0.016^b^ |
| Absolute change in MMDs, mean ± SD | -6.3 ± 5.9 | -5.6 ± 4.9 | -3.5 ± 2.7 | 0.004^c^ |
| **Demographic characteristics** |  |  |  |  |
| Age, mean ± SD, years | 43.9 ± 12.2 | 46.4 ± 12.1 | 45.9 ± 12.8 | 0.076^c^ |
| Female sex, n (%) | 344 (92.2%) | 130 (87.2%) | 41 (85.4%) | 0.11^b^ |
| Racial identity, White, n (%) | 368 (98.7%) | 149 (100.0%) | 47 (97.9%) | 0.23^d^ |
| Overweight (BMI ≥ 25 kg/m^2^), n (%) | 161 (43.5%) | 71 (47.7%) | 12 (25.5%) | 0.025^b^ |
| Obesity (BMI ≥ 30 kg/m^2^), n (%) | 67 (18.0%) | 22 (14.8%) | 4 (8.3%) | 0.20^d^ |
| **Migraine history** |  |  |  |  |
| Age at onset, median (IQR), years | 17 (12–25) | 20 (13–28) | 18 (14–29) | 0.050^e^ |
| Migraine disease duration, median (IQR), years | 24 (15–33) | 23 (15–33) | 24 (14–32) | 0.99^e^ |
| First degree relative with migraine, n (%) | 257 (68.9%) | 110 (73.8%) | 32 (66.7%) | 0.47^b^ |
| Migraine with aura, n (%) | 120 (32.2%) | 37 (24.8%) | 11 (22.9%) | 0.15^b^ |
| Daily headache (28-day baseline), n (%) | 84 (22.5%) | 6 (4.0%) | 0 (0.0%) | <0.001^d^ |
| Medication-overuse, n (%) | 231 (61.9%) | 73 (49.0%) | 20 (41.7%) | 0.002^b^ |
| Headache frequency (28-day baseline), median (IQR) |  |  |  |  |
| MHDs | 21 (15–27) | 14 (11–17) | 8 (7–8) | <0.001^e^ |
| MMDs | 14 (11–18) | 10 (9–13) | 7 (6–8) | <0.001^e^ |
| Monthly days with use of acute medications | 9 (7–13) | 10 (7–12) | 7 (6–8) | <0.001^e^ |
| Use of preventive migraine medication, n (%) | 169 (45.3%) | 83 (55.7) | 27 (56.3%) | 0.057^b^ |
| Preventive medication failures (lack of efficacy), n (%) |  |  |  | 0.27^b^ |
| <3 | 244 (65.4%) | 100 (67.1%) | 37 (77.1%) |  |
| ≥3 | 129 (34.6%) | 49 (32.9%) | 11 (22.9%) |  |
| Triptan resistance (lack of efficacy), n (%) | 76 (21.4%) | 32 (22.5%) | 10 (21.7%) | 0.96^b^ |
| **Disability** |  |  |  |  |
| MIDAS score, median (IQR) | 65 (37–110) | 42 (21–71) | 33 (12–51) | <0.001^e^ |
| HIT-6 score, median (IQR) | 64 (61–66) | 63 (60–65) | 63 (59–64) | 0.003^e^ |
| WHODAS 2.0, median (IQR) | 23 (18–29) | 20 (15–24) | 18 (14–22) | <0.001^e^ |
| **Comorbidities** |  |  |  |  |
| Somatic comorbidities, n (%) |  |  |  |  |
| Autoimmune disorders | 50 (13.4%) | 18 (12.1%) | 4 (8.3%) | 0.69^d^ |
| Asthma | 45 (12.1%) | 7 (4.7%) | 6 (12.5%) | 0.036^b^ |
| Constipation | 73 (19.6%) | 19 (12.8%) | 9 (18.8%) | 0.18^b^ |
| Daily low back pain | 40 (10.7%) | 12 (8.1%) | 1 (2.1%) | 0.12^d^ |
| Daily neck pain | 72 (19.3%) | 8 (5.4%) | 3 (6.3%) | <0.001^d^ |
| Hypertension | 47 (12.6%) | 10 (6.7%) | 4 (8.3%) | 0.13^d^ |
| Psychiatric comorbidities, n (%) |  |  |  |  |
| HADS anxiety score ≥8 | 116 (32.3%) | 31 (21.1%) | 10 (21.7%) | 0.023^b^ |
| HADS depression score ≥8 | 103 (28.7%) | 24 (16.3%) | 4 (8.7%) | <0.001^d^ |
| **Abbreviations:** BMI, body mass index; CM, chronic migraine; EM, episodic migraine; IQR, interquartile range; HADS, Hospital Anxiety and Depression Scale; HIT-6, Headache Impact Test-6; IQR, interquartile range; MIDAS, Migraine Disability Assessment Test; MHDs, monthly headache days; MMDs, monthly migraine days; WHODAS, World Health Organization Disability Assessment Schedule.  a: Participants were classified according to their reduction in MMDs as responders (≥50%) or non-responders (<50%).  b: Pearson’s Chi squared test  c: Analysis of variance (ANOVA)  d: Fisher’s exact test  e: Kruskal-Wallis’ test | | | | |

Supplementary Table 2. Low- and high-frequency episodic migraine (attack features)

|  | **CM**  **(n = 373)** | **High-frequency EM**  **(n = 149)** | **Low-frequency EM**  **(n = 48)** | ***P*^b^** |
| --- | --- | --- | --- | --- |
| **Migraine attack features** |  |  |  |  |
| Unilateral headache, *n* (%) | 321 (86.1%) | 130 (87.2%) | 36 (75.0%) | 0.095^c^ |
| Side-locked | 58 (15.5%) | 19 (12.8%) | 6 (12.5%) | 0.65^c^ |
| Pulsating pain quality, *n* (%) | 288 (77.2%) | 96 (64.4%) | 34 (70.8%) | 0.011^c^ |
| Pain intensity (4-point scale), *n* (%) |  |  |  | 0.27^d^ |
| Mild | 1 (0.3%) | 1 (0.7%) | 1 (2.1%) |  |
| Moderate | 112 (30.0%) | 52 (34.9%) | 16 (33.3%) |  |
| Severe | 260 (69.7%) | 96 (64.4%) | 31 (64.6%) |  |
| Headache aggravated by physical activity, *n* (%) | 342 (91.7%) | 134 (89.9%) | 44 (91.7%) | 0.77^d^ |
| Associated symptoms, *n* (%) |  |  |  |  |
| Photophobia | 356 (95.4%) | 140 (94.0%) | 48 (100.0%) | 0.23^d^ |
| Phonophobia | 345 (92.5%) | 134 (89.9%) | 41 (85.4%) | 0.21^c^ |
| Nausea | 337 (90.3%) | 140 (94.0%) | 45 (93.8%) | 0.39^d^ |
| Vomiting | 208 (55.8%) | 88 (59.1%) | 32 (66.7%) | 0.32^c^ |
| Unilateral cranial autonomic symptoms, *n* (%) | 88 (23.8%) | 25 (16.8%) | 7 (14.6%) | 0.10^c^ |
| Ictal allodynia; ASC-12, median (IQR) | 4 (1–8) | 3 (0–6) | 2 (0–4) | 0.001^e^ |
| **Abbreviations:** ASC-12, Allodynia Symptom Checklist-12; IQR, interquartile range.  a: Participants were classified according to their reduction in MMDs as responders (≥50%) or non-responders (<50%).  b: *P*-values reported for the comparison between responders and non-responders.  c: Pearson Chi-squared test  d: Fisher’s exact test  e: Kruskal-Wallis’ test | | | | |

Supplementary Table 3. Preventive drug class failures

|  | **Total**  **(n = 570)** | | **Responders^a^**  **(n = 298)** | | **Non-responders^a^**  **(n = 272)** | | ***P^b^*** |
| --- | --- | --- | --- | --- | --- | --- | --- |
| **Drug classes** | Tried, N | Failed,  n (%) | Tried, N | Failed,  n (%) | Tried,  N | Failed,  n (%) |  |
| ACE-inhibitors/ARB-II | 486 | 360 (74.1%) | 239 | 171 (71.5%) | 247 | 189 (76.5%) | 0.21^c^ |
| Anti-depressives | 184 | 160 (87.0%) | 81 | 68 (84.0%) | 103 | 92 (89.3%) | 0.28 ^c^ |
| Anti-seizure medications | 383 | 350 (91.4%) | 190 | 176 (92.6%) | 193 | 174 (90.2%) | 0.39 ^c^ |
| Beta-blockers | 393 | 339 (86.3%) | 202 | 173 (85.6%) | 191 | 166 (86.9%) | 0.72 ^c^ |
| Calcium channel blockers | 49 | 48 (98.0%) | 23 | 23 (100.0%) | 26 | 25 (96.2%) | >0.99 ^c^ |
| CGRP-monoclonal antibodies | 1 | 1 (100.0%) | 0 | 0  (0.0%) | 1 | 1 (100.0%) | - |
| Gepants | 0 | 0  (0.0%) | 0 | 0  (0.0%) | 0 | 0  (0.0%) | - |
| OnabotulinumtoxinA | 123 | 55 (44.7%) | 59 | 24 (40.7%) | 64 | 31 (48.4%) | 0.39 ^c^ |
| **Abbreviations:** ACE, angiotensin-converting enzyme; ARB, angiotensin-II receptor blockers; CGRP, calcitonin gene-related peptide.  **Definitions:** Drug class failure was defined as lack of efficacy of tolerability to one or more drugs belonging to the given class, in accordance with the definitions provided by the European Headache Federation guidelines (Sacco *et al*, 2020).  -: not applicable.  a: Participants were classified according to their reduction in MMDs as responders (≥50%) or non-responders (<50%).  b: *P*-values reported for the comparison between responders and non-responders.  c: Pearson’s chi-squared | | | | | | | |

Supplementary Table 4. Performance metrics for the multivariable models

| **Outcome** | **AUC, %**  **(95% CI)** | **Optimism-corrected AUC, %** | **Accuracy^a^,**  **% (95% CI)** | **Sensitivity^a^,**  **% (95% CI)** | **Specificity^a^, % (95% CI)** | **Hosmer-Lemeshow,**  ***p*-value** |
| --- | --- | --- | --- | --- | --- | --- |
| *Primary outcome* |  |  |  |  |  |  |
| ≥50% reduction in MMDs | 64.6  (60.0–69.2) | 62.6 | 62.1  (56.8–65.2) | 62.1  (56.8–67.7) | 60.2  (54.2–66.3) | 0.071 |
| *Secondary outcomes* |  |  |  |  |  |  |
| ≥50% reduction in MHDs | 65.5  (61.0–70.1) | 63.3 | 61.4  (57.4–65.2) | 71.2  (65.1–76.9) | 54.5  (48.9–60.4) | 0.004 |
| ≥50% reduction in MMDs or moderate-to-severe MHDs | 64.7  (60.1–69.3) | 63.1 | 61.9  (58.2–65.8) | 57.9  (52.6–63.2) | 68.7  (62.6–74.9) | 0.18 |
| *Exploratory outcomes* |  |  |  |  |  |  |
| ≥50% reduction in moderate-to-severe MHDs | 66.4  (61.9–70.9) | 64.7 | 63.4  (59.2–67.2) | 56.2  (50.5–61.9) | 70.8  (65.7–76.4) | 0.49 |
| MIDAS reduction:  ≥5 points (baseline score 11–20 points) or ≥30% (baseline score ≥21) | 63.4  (57.5–69.3) | 61.8 | 64.0  (59.7–68.0) | 65.1  (60.0–70.0) | 61.2  (53.0–69.4) | 0.25 |
| HIT-6 reduction: ≥5 points | 66.1  (61.6–70.7) | 64.6 | 62.8  (58.6–66.7) | 68.9  (62.9–74.2) | 56.6  (50.6–62.6) | 0.98 |
| **Abbreviations:** CI, confidence interval; HIT-6, Headache Impact Test-6; MIDAS, Migraine Disability Assessment; MHD, monthly headache days; MMDs, monthly migraine days.  a: optimal threshold determined by Youden’s J statistic. | | | | | | |

Supplementary Table 5. Participants with outcome data

|  | **Responders^a^,**  **n (% of N)** | **Participants with outcome data,**  **N** | **Participants included in multivariate model,**  **n** |
| --- | --- | --- | --- |
| *Primary outcome* |  |  |  |
| ≥50% reduction in MMDs | 298 (52.3%) | 570 | 549 |
| *Secondary outcomes* |  |  |  |
| ≥50% reduction in MHDs | 239 (41.9%) | 570 | 552 |
| ≥50% reduction in MMDs or moderate-to-severe MHDs | 359 (63.0%) | 570 | 570 |
| *Exploratory outcomes* |  |  |  |
| ≥50% reduction in moderate-to-severe MHDs | 291 (51.1%) | 570 | 552 |
| MIDAS reduction:  ≥5 points (baseline score 11–20 points) or  ≥30% (baseline score ≥21) | 351 (72.1%) | 487^b^ | 484 |
| HIT-6 reduction: ≥5 points | 267 (50.2%) | 532 | 532 |
| **Abbreviations:** HIT-6, Headache Impact Test-6; MIDAS, Migraine Disability Assessment; MHD, monthly headache days; MMDs, monthly migraine days.  a: Participants were classified according to their reduction in MMDs as responders (≥50%) or non-responders (<50%).  b: 43 of 530 participants with MIDAS data were not eligible for analysis due to baseline MIDAS score <11 points. | | | |

Supplementary Table 6. Participants with missing baseline data

|  | **Total**  **(N = 570)** | **Responders^a^**  **(N = 298)** | **Non-responders^a^**  **(N = 272)** |
| --- | --- | --- | --- |
| **Demographic characteristics** |  |  |  |
| Age, n/N (%) | 0/570 (0.0) | 0/298 (0.0) | 0/272 (0.0) |
| Female sex, n/N (%) | 0/570 (0.0) | 0/298 (0.0) | 0/272 (0.0) |
| Racial identity, White, n/N (%) | 0/570 (0.0) | 0/298 (0.0) | 0/272 (0.0) |
| Overweight (BMI ≥ 25 kg/m^2^), n/N (%) | 0/570 (0.0) | 0/298 (0.0) | 0/272 (0.0) |
| Obesity (BMI ≥ 30 kg/m^2^), n/N (%) | 0/570 (0.0) | 0/298 (0.0) | 0/272 (0.0) |
| **Migraine history** |  |  |  |
| Age at onset, n/N (%) | 0/570 (0.0) | 0/298 (0.0) | 0/272 (0.0) |
| Migraine disease duration, n/N (%) | 0/570 (0.0) | 0/298 (0.0) | 0/272 (0.0) |
| First degree relative with migraine, n/N (%) | 0/570 (0.0) | 0/298 (0.0) | 0/272 (0.0) |
| Migraine with aura, n/N (%) | 0/570 (0.0) | 0/298 (0.0) | 0/272 (0.0) |
| Chronic migraine, n/N (%) | 0/570 (0.0) | 0/298 (0.0) | 0/272 (0.0) |
| Daily headache (28-day baseline), n/N (%) | 0/570 (0.0) | 0/298 (0.0) | 0/272 (0.0) |
| Medication-overuse, n/N (%) | 0/570 (0.0) | 0/298 (0.0) | 0/272 (0.0) |
| Headache frequency (28-day baseline), n/N (%) |  |  |  |
| MHDs | 0/570 (0.0) | 0/298 (0.0) | 0/272 (0.0) |
| MMDs | 0/570 (0.0) | 0/298 (0.0) | 0/272 (0.0) |
| Monthly days with use of acute medications | 0/570 (0.0) | 0/298 (0.0) | 0/272 (0.0) |
| Use of preventive migraine medication, n/N (%) | 0/570 (0.0) | 0/298 (0.0) | 0/272 (0.0) |
| Preventive medication failures (lack of efficacy), n/N (%) |  |  |  |
| <3 | 0/570 (0.0) | 0/298 (0.0) | 0/272 (0.0) |
| ≥3 | 0/570 (0.0) | 0/298 (0.0) | 0/272 (0.0) |
| Triptan resistance, n/N (%) | 27/570 (4.7) | 16/298 (5.4) | 11/272 (4.0) |
| **Disability** |  |  |  |
| MIDAS score, n/N (%) | 18/570 (3.2) | 11/298 (3.7) | 7/272 (2.6) |
| HIT-6 score, n/N (%) | 15/570 (2.6) | 9/298 (3.0) | 6/272 (2.2) |
| WHODAS 2.0, n/N (%) | 23/570 (4.0) | 14/298 (4.7) | 9/272 (3.3) |
| **Comorbidities** |  |  |  |
| Somatic comorbidities, n/N (%) |  |  |  |
| Autoimmune disorders | 0/570 (0.0) | 0/298 (0.0) | 0/272 (0.0) |
| Asthma | 0/570 (0.0) | 0/298 (0.0) | 0/272 (0.0) |
| Constipation | 0/570 (0.0) | 0/298 (0.0) | 0/272 (0.0) |
| Daily back pain (≥ 3 months) | 0/570 (0.0) | 0/298 (0.0) | 0/272 (0.0) |
| Daily neck pain (≥ 3 months) | 0/570 (0.0) | 0/298 (0.0) | 0/272 (0.0) |
| Hypertension | 0/570 (0.0) | 0/298 (0.0) | 0/272 (0.0) |
| Psychiatric comorbidities, n/N (%) |  |  |  |
| HADS anxiety score ≥8 | 18/570 (3.2) | 11/298 (3.7) | 7/272 (2.6) |
| HADS depression score ≥8 | 18/570 (3.2) | 11/298 (3.7) | 7/272 (2.6) |
| **Abbreviations:** BMI, body mass index; IQR, interquartile range; HADS, Hospital Anxiety and Depression Scale; HIT-6, Headache Impact Test-6; IQR, interquartile range; MIDAS, Migraine Disability Assessment Test; MHDs, monthly headache days; MMDs, monthly migraine days; WHODAS, World Health Organization Disability Assessment Schedule.  a: Participants were classified according to their reduction in MMDs as responders (≥50%) or non-responders (<50%). | | | |

Supplementary Table 7. Participants with missing headache data

|  | **Total**  **(N = 570)** | **Responders^a^**  **(N = 298)** | **Non-responders^a^**  **(N = 272)** |
| --- | --- | --- | --- |
| **Migraine attack features** |  |  |  |
| Unilateral headache, n/N (%) | 0/570 (0.0) | 0/298 (0.0) | 0/272 (0.0) |
| Side-locked | 0/570 (0.0) | 0/298 (0.0) | 0/272 (0.0) |
| Pulsating pain quality, n/N (%) | 0/570 (0.0) | 0/298 (0.0) | 0/272 (0.0) |
| Pain intensity (4-point scale), n/N (%) | 0/570 (0.0) | 0/298 (0.0) | 0/272 (0.0) |
| Mild |  |  |  |
| Moderate |  |  |  |
| Severe |  |  |  |
| Headache aggravated by physical activity, n/N (%) | 0/570 (0.0) | 0/298 (0.0) | 0/272 (0.0) |
| Associated symptoms n/N (%) |  |  |  |
| Photophobia | 0/570 (0.0) | 0/298 (0.0) | 0/272 (0.0) |
| Phonophobia | 0/570 (0.0) | 0/298 (0.0) | 0/272 (0.0) |
| Nausea | 0/570 (0.0) | 0/298 (0.0) | 0/272 (0.0) |
| Vomiting | 0/570 (0.0) | 0/298 (0.0) | 0/272 (0.0) |
| Unilateral cranial autonomic symptoms, n/N (%) | 4/570 (0.7) | 4/298 (1.3) | 0/272 (0.0) |
| Ictal cutaneous allodynia; ASC-12, n/N (%) | 21/570 (3.7) | 13/298 (4.4) | 8/272 (2.9) |
| **Abbreviations:** ASC-12, Allodynia Symptom Checklist-12; IQR, interquartile range.  a: Participants were classified according to their reduction in MMDs as responders (≥50%) or non-responders (<50%). | | | |

Supplementary Table 8. Key predictive characteristics according to response

|  |  |  | **Treatment response**  **(reduction in MMDs)** | | |
| --- | --- | --- | --- | --- | --- |
|  |  |  | **≥50%** | **30–49%** | **<30%** |
| **Characteristic** | | n | % of n | % of n | % of n |
| **Total population** | - | 570 | 52.3 | 18.2 | 29.5 |
| **CM** | CM | 373 | 48.5 | 19.3 | 32.2 |
|  | EM | 197 | 59.4 | 16.2 | 24.4 |
| **Daily headache** | Present | 90 | 33.3 | 22.2 | 44.4 |
|  | Absent | 480 | 55.8 | 17.5 | 26.7 |
| **Preventive medication failures (lack of efficacy)** | ≥3 | 189 | 41.8 | 22.8 | 35.4 |
|  | <3 | 381 | 57.5 | 16.0 | 26.5 |
| **Abbreviations:** CM, chronic migraine; EM, episodic migraine; MMDs, monthly migraine days. | | | | | |

Supplementary Table 9. Absolute response in partial responders

|  | **Participants with partial response**  **(30–49% reduction in MMDs; n = 104):** | | |
| --- | --- | --- | --- |
| **Efficacy outcomes** | CM  (n = 72) | Daily headache  (n = 20) | ≥3 preventive medication failures  (n = 43) |
| Absolute change in MMDs, mean (SD) | -5.8 (2.4) | -6.8 (3.6) | -5.8 (2.9) |
| Absolute change in MHDs, mean (SD) | -5.5 (4.3) | -3.5 (4.6) | -5.9 (4.3) |
| Absolute change in moderate-to-severe MHDs, mean (SD) | -5.1 (4.1) | -5.5 (4.0) | -6.0 (3.9) |
| Absolute change in acute medication days, mean (SD) | -4.0 (3.9) | -2.7 (5.0) | -3.6 (3.4) |
| Clinical meaningful response in MIDAS, % (n) | 68.4% (39)^a^ | 52.6% (n = 10)^a^ | 68.6% (n = 24)^a^ |
| Clinical meaningful response in HIT-6, % (n) | 43.9% (29)^b^ | 31.6% (n = 6)^b^ | 45.0% (n = 18)^b^ |
| **Abbreviations:** CM, chronic migraine; HIT-6, Headache Impact Test-6; MIDAS, Migraine Disability Assessment score; MMDs, monthly migraine days.  a: missing data in 15 partial responders with CM, 1 with daily headache, and 8 with ≥3 preventive medication failures  b: missing data in 6 partial responders with CM, 1 with daily headache, and 3 with ≥3 preventive medication failures | | | |

Supplementary Table 10. Early and late responders (baseline characteristics)

|  | **Early responders^a^**  **(n = 217)** | **Late responders^a^**  **(n = 79)** | ***P*^b^** |
| --- | --- | --- | --- |
| **Demographic characteristics** |  |  |  |
| Age, mean ± SD, years | 46.9 ± 11.7 | 43.7 ± 13.0 | 0.053^c^ |
| Female sex, n (%) | 193 (88.9%) | 70 (88.6%) | >0.99^e^ |
| Racial identity, White, n (%) | 216 (99.5%) | 77 (97.5%) | 0.17^f^ |
| Overweight (BMI ≥25 kg/m^2^), n (%) | 85 (39.2%) | 34 (43.0%) | 0.59^e^ |
| Obesity (BMI ≥30 kg/m^2^), n (%) | 28 (12.9%) | 11 (13.9%) | 0.81^e^ |
| **Migraine history** |  |  |  |
| Age at onset, median (IQR), years | 18 (13–27) | 15 (10–23) | 0.061^d^ |
| Migraine disease duration, median (IQR), years | 26 (17–35) | 24 (15–38) | 0.61^d^ |
| First degree relative with migraine, n (%) | 151 (69.6%) | 55 (69.6%) | >0.99^e^ |
| Migraine with aura, n (%) | 61 (28.1%) | 26 (32.9%) | 0.42^e^ |
| Chronic migraine, n (%) | 119 (54.8%) | 61 (77.2%) | <0.001^e^ |
| Daily headache (28-day baseline), n (%) | 19 (8.8%) | 11 (13.9%) | 0.19^e^ |
| Medication-overuse, n (%) | 124 (57.1%) | 48 (60.8%) | 0.58^e^ |
| Headache frequency (28-day baseline), median (IQR) |  |  |  |
| MHDs | 15 (12–21) | 19 (14–26) | 0.003^d^ |
| MMDs | 12 (9–15) | 13 (10–18) | 0.081^d^ |
| Monthly days with use of acute medications | 9 (7–12) | 8 (7–12) | 0.39^d^ |
| Use of preventive migraine medication, n (%) | 110 (50.7%) | 40 (50.6%) | 0.99^e^ |
| Preventive medication failures (lack of efficacy), n (%) |  |  | 0.079^e^ |
| <3 | 165 (76.0%) | 52 (65.8%) |  |
| ≥3 | 52 (24.0%) | 27 (34.2%) |  |
| Triptan resistance (lack of efficacy), n (%) | 39 (18.8%) | 15 (20.5%) | 0.75^e^ |
| **Disability** |  |  |  |
| MIDAS score, median (IQR) | 52 (30–85) | 65 (35–120) | 0.029^d^ |
| HIT-6 score, median (IQR) | 63 (61–65) | 64 (62–66) | 0.16^d^ |
| WHODAS 2.0, median (IQR) | 21 (17–28) | 22 (18–30) | 0.11^d^ |
| **Comorbidities** |  |  |  |
| Somatic comorbidities, n (%) |  |  |  |
| Autoimmune disorders | 31 (14.3%) | 11 (13.9%) | 0.94^e^ |
| Asthma | 24 (11.1%) | 9 (11.4%) | 0.94^e^ |
| Constipation | 42 (19.4%) | 14 (17.7%) | 0.75^e^ |
| Daily low back pain | 21 (9.7%) | 10 (12.7%) | 0.46^e^ |
| Daily neck pain | 33 (15.2%) | 17 (21.5%) | 0.20^e^ |
| Hypertension | 21 (9.1%) | 6 (7.6%) | 0.58^e^ |
| Psychiatric comorbidities, n (%) |  |  |  |
| HADS anxiety score ≥8 | 54 (26.0%) | 29 (37.7%) | 0.054^e^ |
| HADS depression score ≥8 | 42 (20.2%) | 21 (27.3%) | 0.20^e^ |
| **Abbreviations:** BMI, body mass index; IQR, interquartile range; HADS, Hospital Anxiety and Depression Scale; HIT-6, Headache Impact Test-6; IQR, interquartile range; MIDAS, Migraine Disability Assessment Test; MHDs, monthly headache days; MMDs, monthly migraine days; WHODAS, World Health Organization Disability Assessment Schedule.  a: Early responders were defined as participants with a ≥50% reduction in MMDs from baseline during weeks 1 through 12 and weeks 13 through 24. Late responders were participants who did not achieve ≥50% reduction in MMDs before weeks 13 through 24.  b: *P*-values reported for the comparison between responders and non-responders.  c: Unpaired t test  d: Mann–Whitney *U* test  e: Pearson Chi-squared test  f: Fisher’s exact test | | | |

Supplementary Table 11. Early and late responders (attack features)

|  | **Early responders^a^**  **(n = 217)** | **Late responders^a^**  **(n = 79)** | ***P*^b^** |
| --- | --- | --- | --- |
| **Migraine attack features** |  |  |  |
| Unilateral headache, *n* (%) | 193 (88.9%) | 63 (79.7%) | 0.041^c^ |
| Side-locked | 31 (14.3%) | 11 (13.9%) | 0.94 ^c^ |
| Pulsating pain quality, *n* (%) | 153 (70.5%) | 64 (81.0%) | 0.071^c^ |
| Pain intensity (4-point scale), *n* (%) |  |  | 0.32^d^ |
| Mild | 0 (0.0%) | 1 (1.3%) |  |
| Moderate | 66 (30.4%) | 25 (31.6%) |  |
| Severe | 151 (69.6%) | 53 (67.1%) |  |
| Headache aggravated by physical activity, *n* (%) | 198 (91.2%) | 77 (97.5%) | 0.075^d^ |
| Associated symptoms, *n* (%) |  |  |  |
| Photophobia | 208 (95.9%) | 75 (94.9%) | 0.75^d^ |
| Phonophobia | 196 (90.3%) | 74 (93.7%) | 0.49^d^ |
| Nausea | 202 (93.1%) | 74 (93.7%) | >0.99^d^ |
| Vomiting | 128 (59.0%) | 48 (60.8%) | 0.78^c^ |
| Unilateral cranial autonomic symptoms, *n* (%) | 41 (19.1%) | 19 (24.7%) | 0.30^c^ |
| Ictal allodynia; ASC-12, median (IQR) | 4 (0–8) | 6 (2–8) | 0.024^e^ |
| **Abbreviations:** ASC-12, Allodynia Symptom Checklist-12; IQR, interquartile range.  a: Participants were classified according to their reduction in MMDs as responders (≥50%) or non-responders (<50%).  b: *P*-values reported for the comparison between responders and non-responders.  c: Pearson Chi-squared test  d: Fisher’s exact test  e: Mann–Whitney *U* test | | | |
